# HVRLocator: a computationally efficient tool for identifying hypervariable regions in large 16S rRNA datasets

**DOI:** 10.1093/gigascience/giag040

**Published:** 2026-04-08

**Authors:** Clara Arboleda-Baena, Felipe Borim Corrêa, João Pedro Saraiva, Santiago Castillo-Rivadeneira, Jonas Coelho Kasmanas, Antonis Chatzinotas, Stephanie D Jurburg

**Affiliations:** German Centre for Integrative Biodiversity Research (iDiv) Halle-Jena-Leipzig, Puschstraße 4, 04103 Leipzig, Germany; Department of Applied Microbial Ecology, Helmholtz Centre for Environmental Research - UFZ, Permoserstraße 15, 04318 Leipzig, Germany; Department of Applied Microbial Ecology, Helmholtz Centre for Environmental Research - UFZ, Permoserstraße 15, 04318 Leipzig, Germany; Department of Applied Microbial Ecology, Helmholtz Centre for Environmental Research - UFZ, Permoserstraße 15, 04318 Leipzig, Germany; German Centre for Integrative Biodiversity Research (iDiv) Halle-Jena-Leipzig, Puschstraße 4, 04103 Leipzig, Germany; Department of Applied Microbial Ecology, Helmholtz Centre for Environmental Research - UFZ, Permoserstraße 15, 04318 Leipzig, Germany; German Centre for Integrative Biodiversity Research (iDiv) Halle-Jena-Leipzig, Puschstraße 4, 04103 Leipzig, Germany; Department of Applied Microbial Ecology, Helmholtz Centre for Environmental Research - UFZ, Permoserstraße 15, 04318 Leipzig, Germany; Institute of Biology, Leipzig University, Talstraße 33, 04103 Leipzig, Germany; German Centre for Integrative Biodiversity Research (iDiv) Halle-Jena-Leipzig, Puschstraße 4, 04103 Leipzig, Germany; Department of Applied Microbial Ecology, Helmholtz Centre for Environmental Research - UFZ, Permoserstraße 15, 04318 Leipzig, Germany

**Keywords:** big data, 16S rRNA gene, metabarcoding, high throughput sequencing, metadata, microbial ecology

## Abstract

**Background:**

Metabarcoding of the 16S rRNA gene is widely used to assess microbial diversity due to its cost-effectiveness and efficiency. However, publicly available 16S rRNA metabarcoding datasets often lack standardized metadata, particularly information on the sequenced hypervariable regions or primers used, which are critical to their accurate reuse. To address this, we present HVRLocator, a computational tool that (1) identifies the start and end positions of 16S rRNA amplicons, (2) determines their corresponding hypervariable regions, and (3) detects the presence of primer sequences. This tool was validated on four datasets comprising 41,513 samples generated with different primers and sequencing platforms.

**Results:**

HVRLocator can process archived 16S rRNA sequences from NCBI SRA at an average rate of 6.5 samples per minute. Validation showed it reliably detects amplicon start and end positions across datasets sequenced with different primers and platforms, achieving 100% accuracy within single-platform studies and correctly revealing length heterogeneity across platforms. It also flagged misannotated metadata and problematic sequences, underscoring its value as a sequence data curation tool. Finally, HVRLocator can select comparable sequences to build large 16S rRNA amplicon databases spanning the same hypervariable region, facilitating cross-study comparisons.

**Conclusion:**

HVRLocator overcomes unreliable metadata by accurately identifying 16S rRNA amplicon start and end positions, determining hypervariable regions, and detecting primer sequences, enabling accurate curation and large-scale processing of 16S rRNA data for reliable and reproducible microbial studies, syntheses, and meta-analyses.

## Background

While the existence of bacteria has been known for over three centuries, the ability to study all individuals in a bacterial community is relatively novel. By extracting and sequencing nucleic acids from hosts or environmental samples, it is now possible to characterize the taxonomic diversity of a bacterial community without the need to cultivate its members. Metabarcoding, which focuses sequencing efforts on a segment of a universal marker gene, or amplicon (typically the 16S rRNA gene for prokaryotes), has emerged as a dominant technique due to its technical ease and low cost. To date, 16S rRNA gene metabarcoding has uncovered the extreme diversity and ubiquity of microbes [1], while revealing avenues for improving human health, agricultural productivity, and sustainability [2]. At the same time, metabarcoding datasets archived in public repositories have grown exponentially [3]. These data are uniform in format, are routinely archived with technical and experimental metadata, and are a rich and growing resource for synthetic and large-scale research. However, metabarcoding data are archived in their raw format, and the metadata needed for bioinformatics processing is often unavailable or not curated, creating barriers to data reuse [3, 4].

Technical metadata are central to sequence data harmonization and reuse as they provide context for the data [5] and directly inform bioinformatics processing. Technical choices preceding sequencing, most notably the DNA extraction kit [6], sequencing platform [7], and target amplicon [8, 9] have been shown to affect microbial diversity assessments [10]; however, the integration of these data *in light* of Big Data processing and synthesis has received less attention. For example, Abdill and colleagues [11] restricted their synthesis to sequences obtained from Illumina technologies to use a unified processing pipeline but did not enforce consistent amplicon sequence lengths, even though detected bacterial diversity increases linearly with amplicon length [12].

The 16S rRNA gene contains both highly conserved regions that are essential for primer design, and hypervariable regions that allow for the phylogenetic identification of microorganisms [13]. Full-length 16S rRNA gene sequences (~1,500 bp) comprise nine hypervariable regions interspersed with nine highly conserved regions [14, 15]. Identifying which 16S rRNA gene segment was targeted for amplification is crucial to fully leverage sequence length and coverage, which ultimately determines the efficiency and accuracy of downstream processing and taxonomic classification pipelines. In the case of paired-end reads (e.g., Illumina technologies), the length of the target region further informs the minimum read length needed to achieve a successful merger of the pair [16].

At the same time, the choice of the sequenced region can significantly affect the relative abundances of detected organisms. For example, Wasimuddin et al. [8] found that compared to three other primer sets targeting different regions, the primer pair targeting the V4 hypervariable region of the 16S rRNA gene produced the highest estimates of amplicon sequence variants (ASVs) richness and diversity across various sample types (including soil, maize roots, cattle rumen, and cattle and human feces). However, different primers targeting the same variable region can still generate different numbers of ASVs [17].

Crucially, as novel sequencing technologies and platforms have emerged, the length of the target amplicons has also varied extensively, ranging from 150 base pair segments (e.g., Illumina HiSeq single-end sequencing) to whole genes (e.g., nanopore sequencing), resulting in massive heterogeneity in the length and location of the sequenced regions. Variation in amplicon length complicates the reuse of 16S rRNA metabarcoding data, since longer sequences tend to detect greater bacterial diversity and taxonomic resolution [12].

Considering technical information about the sequenced region during downstream bioinformatics processing is essential to metabarcoding data reuse, but this information is often missing from the metadata, vague, or incorrect [18]. Standardized metadata is structured information that follows agreed-upon standards, ensuring consistency and comparability across studies and databases through defined fields, controlled vocabularies, or ontologies, and standardized formats. The lack of standardized metadata significantly slows down the compilation of large datasets, making it difficult to reprocess metabarcoding sequences collectively. Here, we present HVRLocator, a computational tool designed to efficiently identify hypervariable regions of the 16S rRNA gene sequenced for a set of metabarcoding samples. By optimizing computational resources, our approach enables rapid and accurate screening of large datasets, facilitating more comprehensive and scalable microbial diversity analyses.

## Materials and methods

### Design

HVRLocator identifies which segment of the 16S rRNA gene was sequenced for a given set of metabarcoding samples. The full pipeline uses Python programming language, and users can access it either via a singularity container or by installing it locally on their computer. For further details on installation and usage please see: HVRLocator GitHub web page [19].

As input, HVRLocator accepts text file (.txt) lists of accession numbers compliant with International Nucleotide Sequence Collaboration (INSDC) databases, including the European Nucleotide Archive (ENA) at the European Bioinformatics Institute, the Sequence Read Archive (SRA) at the National Center for Biotechnology Information, and the DNA Databank of Japan (DDBJ) Sequence Read Archives at the National Institute of Genetics [20], bypassing the need to download the data a priori. HVRLocator also accepts resolved amplicons (e.g., ASV) or raw sequencing data in. FASTA or. FASTQ file formats of the 16S rRNA gene.

For INSDC data, the tool employs a multi-step process. First, it retrieves 1,000 reads using fastq-dump from the SRA Toolkit [21] and performs quality control and trimming with fastp [22]. Then, for each sample, the HVRLocator identifies the data type (single- or paired-end DNA). For paired-end reads, it merges the processed reads using VSEARCH [23], while for single-end reads, it directly converts the trimmed FASTQ to FASTA format. The tool then aligns the processed sequences to a reference 16S rRNA gene sequence from an *Escherichia coli* reference genome (J01859.1) using MAFFT [24]. HVRLocator subsequently analyzes the aligned sequences to determine hypervariable regions by identifying the median start and end alignment positions relative to the *E. coli* 16S rRNA gene reference sequence, using established coverage thresholds (default = 0.6) for the conserved and hypervariable regions of the 16S rRNA gene [13, 25]. To note, the thresholds can be adjusted by adding the “-t” flag. The output is a tab-separated values (TSV) file containing alignment start and end positions, as well as the boundaries (median and average start and end positions, and minimum start and maximum end positions) of the identified hypervariable regions. HVRLocator is currently limited to bacterial 16S rRNA sequences, as it aligns them to the *E. coli* 16S rRNA reference.

Finally, a random forest (RF) model was designed to predict the presence of a primer in a given SRA sequencing dataset by analyzing the quality score distribution of the initial subset of reads. RF performs well under moderate class imbalances when class-aware evaluation and sampling are applied [26, 27]. Additionally, distinct quality score patterns are known to occur in the first few cycles of Illumina sequencing when base diversity is low [28] as is the case of untrimmed primers at the start of reads. Quality score patterns have also been used to detect sequencing bias and artifacts by tools such as DADA2 [29] and Mapinsights [30]. Thus, this metric can serve as a proxy for detecting primer presence. To this end, we selected a curated collection of SRA samples with (882 samples) and without primers (8940 samples, Supplementary Tables S1 and S2). For each sample, the first 1,000 reads from each sample were extracted using fastq-dump (NCBI SRA Toolkit, 3.2.1), and two quality score segments from positions 1–5 and 6–10 were calculated. Eight statistical features were obtained: count, mean, median, standard deviation, minimum, maximum, an estimate of skewness (approximated by the 25th percentile), and kurtosis (approximated by the 75th percentile), resulting in 16 features per sample. The model was trained using scikit-learn’s RandomForestClassifier (v1.2.1) with 100 estimators and a fixed random seed (random_state=42), using an 80/20 stratified train-test split. The RF model yielded a precision of 99.96% for the dataset without primers and 100% for the dataset with primers. Recall of the model using the “no-primer” and “primer” dataset was 100% and 99.55%, respectively. Full details on the model generation including the algorithm, versions, and packages are available in the Supplementary Table S3.

HVRLocator is available as a singularity container [31], and can be executed on high-performance computing (HPC) clusters or cloud computing platforms. Further, Singularity enables the seamless execution of containers without requiring root privileges, maintaining security and reproducibility. HVRLocator will be actively maintained with updates for compatibility and user feedback. Issues or feature requests can be sent to the corresponding author. The HVRLocator output is a text file (.txt) containing the following columns:

**1.Sample_ID:** Identifier of the processed sample (run accession number).
**2.Primer Presence:** Presence or absence of a primer (TRUE/FALSE)
**3.Score Primer Presence:** The associated probability value ranging from 0 to 1.
**4.Min/Max Alignment Start/End:** Minimum (0) and maximum (1540) possible alignment positions along the 16S rRNA gene.
**5.Average Alignment Start/End:** The mean position where reads align to the 16S rRNA gene. The *start* indicates the average starting position, and the *end* indicates the average ending position across all reads in the sample.
**6.Median Alignment Start/End:** The median position where reads align to the 16S rRNA gene. The start indicates the median starting position, and the end indicates the median ending position across all reads in the sample.
**7.Predicted HV region Start/End:** Predicted hypervariable (HV) region based on the median alignment start and end positions across all reads, inferred from literature on conserved and hypervariable regions of the 16S rRNA gene ([25], [13]).
**8.Coverage based HV region Start/End:** Predicted hypervariable region based on coverage at the start and end positions across all reads.
**9.Coverage HV region Start/End:** Coverage values (0–1) for the “*Coverage based HV region*” start or end position across all reads. 0 = no reads cover that position; 1 = all reads cover that position
**10.Warnings:** Alerts about low coverage regions. See possible errors and troubleshooting.
**11–19. Cov_V1 to Cov_V9:** Coverage values (0–1) for each HV region.

Importantly, validation showed that the median alignment position is a more reliable indicator of the sequenced region than the average alignment position, as low-quality sequences within a sample can skew the mean and lead to an incorrect identification of the hypervariable region. For this reason, we report both metrics but recommend prioritizing the median when deciding whether to retain or discard sequences during downstream processing. Additionally, HVRLocator provides the underlying probability scores for primer presence, allowing users to manually adjust filtering based on their own criteria instead of relying solely on the default TRUE/FALSE classification. The default 0.5 threshold was validated using the RF model, and any alternative threshold chosen by the user is applied at their own discretion.

Finally, in addition to identifying the hypervariable region based on the average and median start and end positions of the amplicons within each sample, HVRLocator also reports coverage across all nine hypervariable regions for every individual sample. This allows users to assess the amplicon length, determine its exact start and end positions, and evaluate the coverage of specific hypervariable region(s) within each sample.

### Validation

HVRLocator’s processing stability was calculated by measuring the sample time for 1, 10, 100, 1,000, and 10,000 samples using the same cluster resources: 8 GB of RAM and 4 CPU cores to emulate the standard capabilities of a personal computer.

HVRLocator was validated by analyzing four datasets that contained samples sequenced using (a) same primer and same sequencing platform, (b) different primers and the same platform, (c) the same primer and different sequencing platforms, and (d) different primers and sequencing platforms. Dataset *a* included 17,537 samples from the Earth Microbiome Project [32], in which all samples were sequenced on the Illumina Miseq platform using the 515F-806R primer set targeting the V4 hypervariable region of the 16S rRNA gene [32]. Dataset *b* included 242 samples from two studies that compared different primers but were sequenced on the same platform [8, 33]. Dataset *c* included 18,426 samples from the MiCoDa database Version 1, which were compiled from available literature and data [34], to select samples that were sequenced in the same 515–806 region of the 16S SSU rRNA (small subunit ribosomal RNA) with various sequencing platforms. The primer information for each sample was obtained from the metadata archive in NCBI. For dataset *d*, we selected 5,308 samples compiled during the Datathon project in Latin America [35]. These samples employed various primer sets targeting different regions of the 16S rRNA gene and were sequenced on different platforms, and represent a realistic set of samples that might be encountered during data compilation efforts. The run accession numbers used for all datasets are listed in Supplementary Table S4, 1–4.

All graphics were carried out in R with RStudio interface [36], and all pipelines are available at GitHub [37].

## Results

**Figure 1:**
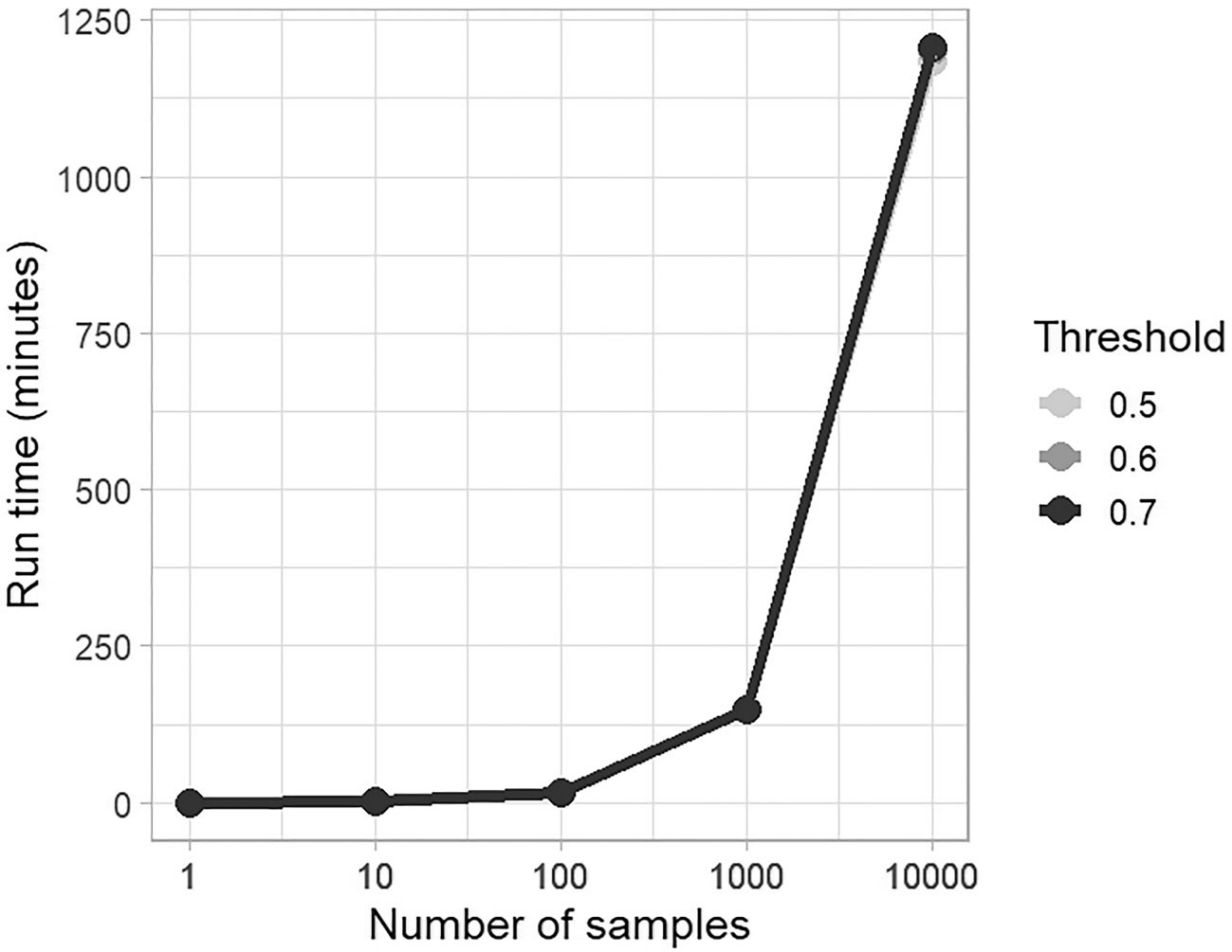
Relationship between the number of samples and run time (in minutes) using 8 GB of RAM and 4 CPU cores. We randomly selected sample numbers from the Earth Microbiome Project (Dataset 1), MiCoDa V1 (Dataset 2), and Datathon activities (Dataset 4). All samples were downloaded from the NCBI.

As the number of samples increased, the running time per sample and computational resource usage remained stable at an average rate of 6.5 samples per minute (Fig. 1 and Supplementary Table S5), highlighting the tool’s processing stability and scalability for the analysis of large datasets. Failures were primarily due to samples with fewer than 500 reads (72%), alignment errors (14%), missing FASTQ files (13%), and NCBI portal-related issues (1%). (See *Possible Errors and Troubleshooting* at the HVRLocator GitHub web page [19]). Although HVRLocator incorporates an automatic retry mechanism to mitigate temporary NCBI portal interruptions, a small number of samples failed due to persistent external server-side issues beyond the control of the tool.

**Figure 2:**
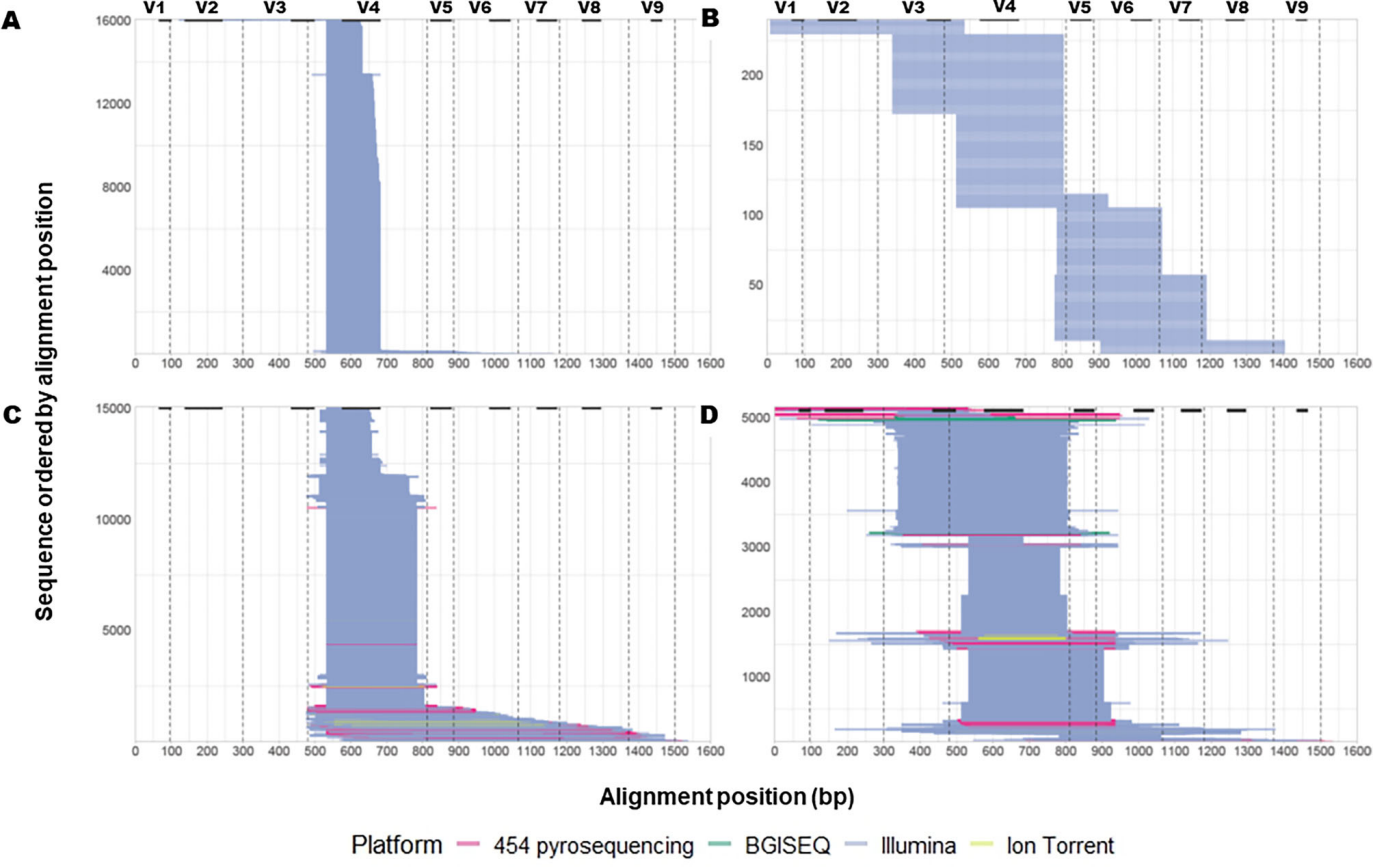
Alignment positions across 16S rRNA regions and sequencing setups. A) Same 16S rRNA region and sequencing setup (*N* = 16059 samples); B) Different 16S rRNA regions, same sequencing setup (*N* = 239 samples); C) Same 16S rRNA region, different sequencing setups (*N* = 15049 samples); D) Different 16S rRNA regions and sequencing setups (*N* = 5113 samples). The upper part of the figure, along with the dashed lines, indicates the start and end positions of the sequencing setups used to assign specific regions of the 16S rRNA gene (modified from [13]). The hypervariable regions corresponding to each setup are highlighted with bold black bars ([25]).

By analyzing the alignment positions across different 16S rRNA hypervariable regions and sequencing setups (Fig. 2), we found that HVRLocator accurately predicted the alignment positions compared to what is expected from the literature associated with each database. For example, sequences in dataset *a*, consistently aligned with the V4 hypervariable region of the 16S rRNA gene and had a median sequence start of 532 bp, as expected from the standardized primer set (515F-806R) used in the Earth Microbiome Project [1]. Sequence lengths were highly homogeneous, also likely due to the use of the same primer set and sequencing machinery (Fig. 2a). For a total of 17,537 samples, 16,059 samples were processed successfully without warnings; common issues included missing FASTQ files, low reads, alignment failures, and NCBI portal-related issues. Out of 16,059 samples processed, 9 did not yield the expected results based on the literature.

**Figure 3:**
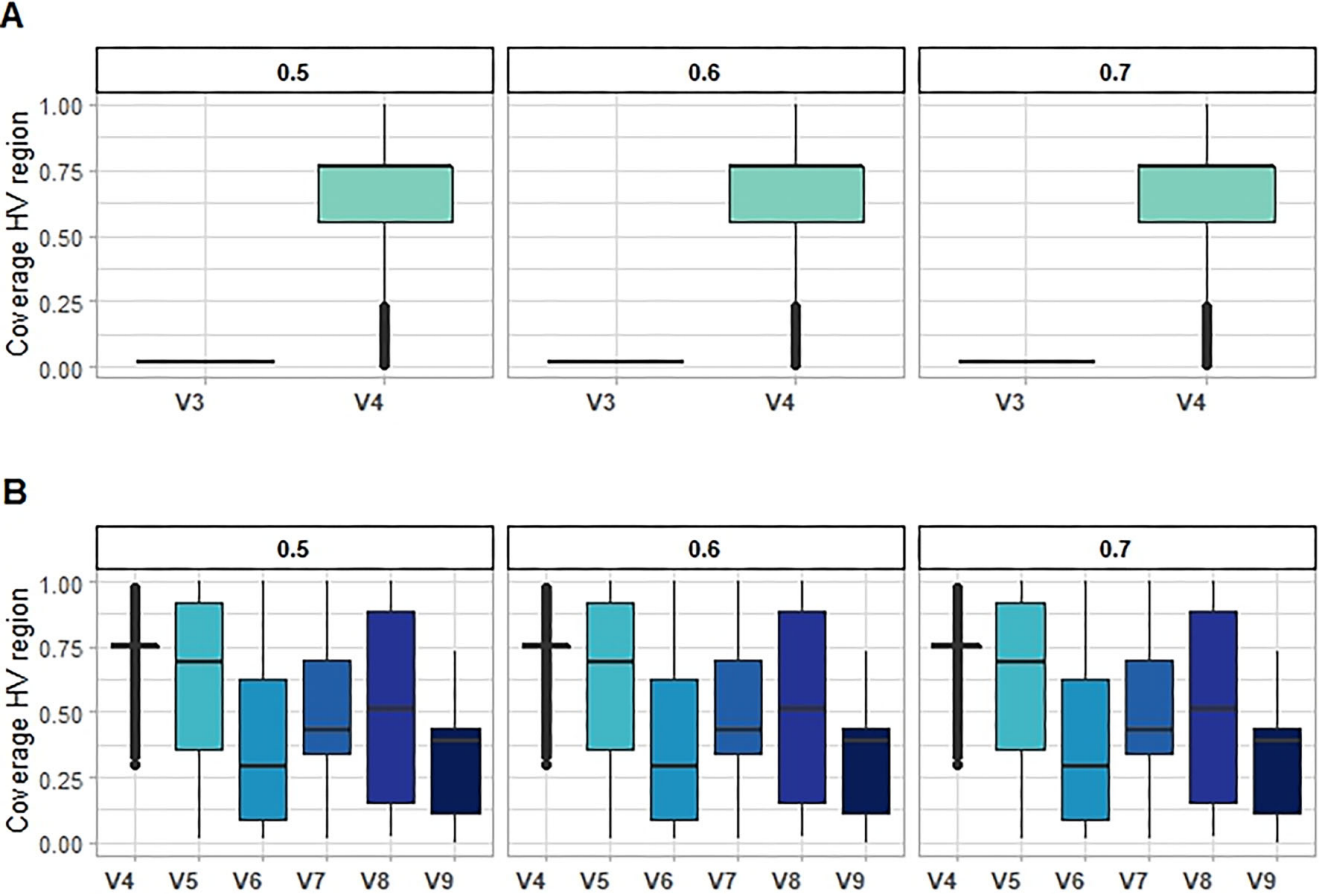
Differences in 16S rRNA gene coverage using the same primer set (Primer 515R-806R for V4 region) but different sequencing setups. A) Predicted 16S rRNA region coverage start, B) Predicted 16S rRNA region coverage end.

Dataset *b* [8, 33], which employed different primers but the same platform, confirmed that HVRLocator correctly matched the sequences to the corresponding, expected primer regions used during sample sequencing with 100% accuracy (Fig. 2b). For dataset *c* HVRLocator also accurately indicated more heterogeneous alignment start positions and sequence lengths, consistent with our expectation and the compilation of the MiCoDa database from the literature (Fig. 2c). Most sequences covered the V4 region (89%), as indicated by both the median alignment start and the coverage-based HV region start (Fig. 2c and Fig. 3, respectively). As expected, the median alignment end varied across projects and sequencing platforms.

To check the reliability of HVRLocator relative to manual extraction of metadata from the literature, we manually extracted data related to the primers used and the 16S rRNA HV region targets from all samples in dataset *c*. For a total of 18,426 samples, 16,771 samples were processed successfully without warnings; common issues included missing FASTQ files, low reads, alignment failures, and NCBI portal-related issues. Of the 16,771 samples processed, 1,712 (10%) did not produce results consistent with the literature (e.g., mismatches between the start region alignment and the reported primer, or incorrect HV region alignment compared with the reported HV region), underscoring the value of obtaining metadata from the sequence data directly, rather than from the literature. Finally, for the diverse data set that used both different 16S rRNA regions and sequencing setups (Fig. 2d), HVRLocator accurately and rapidly assigned the alignment positions.

Importantly, validation highlighted HVRLocator tool’s ability to identify problematic sequences. For example, we observed 932 samples with abnormally long average sequence lengths (i.e., >600 bp) that exceeded the expected output lengths with Illumina platforms. Upon manually reviewing these sequences, we found that either the sequencing platform was incorrectly annotated in the metadata (NCBI or the associated publication), or the sequences did not correspond to the 16S rRNA gene but rather to the internal transcribed spacer (ITS) region or the nifH gene. This highlights the use of HVRLocator as a curation tool for large datasets, where human errors in annotation can significantly impact downstream analysis. For further details, see Supplementary Table S6.

### Case study: 45,882 metabarcoding samples for the compilation of a large 16S rRNA gene database

We present an example of how to use the tool to select correct and comparable sequences to construct a large bacterial database based on metabarcoding sequences targeting the V4 hypervariable region of the 16S rRNA gene. We included samples sequenced from start position 515 bp of the 16S rRNA gene, which is the same starting position as that used by the Earth Microbiome Project primers (515F-806R) [1]. Our input dataset included 45,882 samples spanning a wide variety of matrices (e.g., soil, host-associated, and water), that were sequenced with different primer sets and sequencing platforms. These data were collected through an extensive literature search prioritizing meta-analyses, large amplicon research consortia, and Datathon activities [35] (Supplementary Table S7). Using SRA’s sequence-associated metadata, we selected only metabarcoding-derived sequences (i.e., excluding WGA, WGS, Tn-Seq, miRNA-Seq, POOLCLONE, RNA-Seq, etc.).

**Figure 4:**
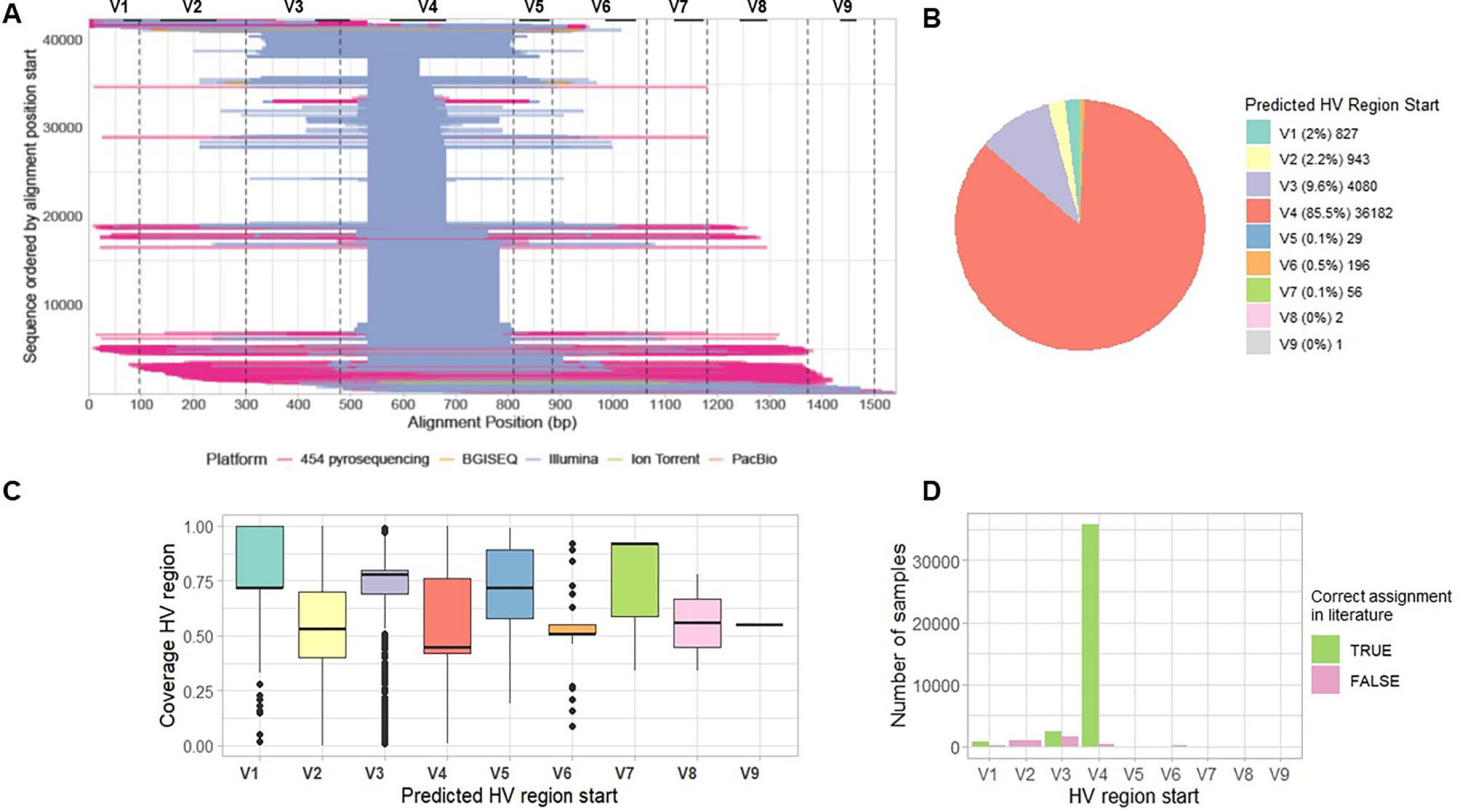
Application of HVRLocator for the selection of V4 16S rRNA gene metabarcoding samples from MiCoDa V2. A) Alignment start positions across the 16S rRNA gene for the 42,316 samples analyzed, B) Percentage of samples retained for downstream analyses after applying the HVRLocator tool, C) Variation in gene coverage across sequences, D) Number of samples in which primer assignment matched the metadata.

HVRLocator processed samples at an average rate of 6.5 samples per minute, using 8 GB of RAM and 4 CPU cores. A total of 42,166 samples were processed successfully, while 3,716 samples failed to be processed and generated warnings, mainly due to samples with fewer than 500 reads (66%), missing FASTQ files (21%), alignment errors (13%), and other issues related to the NCBI portal (1%). (See *Possible Errors and Troubleshooting* at the HVRLocator GitHub web page [19]). HVRLocator identified a diverse range of sequences with varying median hypervariable region start and end positions, coverage, and lengths (Fig. 4a). The output indicated that 1,532 samples had a true presence of primers, while 40,634 did not.

The detailed output for the average and median alignment columns, as well as the coverage-based start and end values, is provided in Supplementary Table S8a–d, f. Of the successfully processed samples, 85.9% (36,217) began in the V4 region of the 16S rRNA gene (Fig. 4b), and the next most common starting regions were V3 (9.7%), V2 (2.3%), and V1 (1.5%). The predicted end of the hypervariable region for the majority of samples corresponded to the V4 region (85.3%), followed by V6 (6.5%), V8 (3.4%), V5 (1.6%), and V7 (1.4%). (Supplementary Table S8e). We retained the 36,217 samples that had a median starting point in the V4 region.

For all sequences, we cross-checked the reported primer information either from the metadata of the research articles or NCBI records against the region predicted by HVRLocator (Fig. 4d). This allowed us to quantify the number of samples where the actual sequenced region was incorrectly assigned, despite being labeled as targeting the V4 hypervariable region in the metadata. Among the selected samples, 382 (1%) had incorrect primer annotations, either in the NCBI metadata or in the associated publications. These findings highlight that even when metadata is available, it may be inaccurate or misleading and underscores the importance of HVRLocator, which accurately and efficiently identifies the sequenced region.

## Discussion

INSDC databases currently host over 32 million next-generation sequencing samples [38], and represent a growing resource for large-scale analyses to address global questions through the synthesis and reuse of sequence data. However, efforts of sequence data archiving are undermined by the lack of available metadata [21], especially, as these metadata are crucial to data processing. High-quality data are relatively sparse [3], which makes the process of data identification intensive, inefficient, and error-prone. To facilitate the reuse of bacterial metabarcoding data, we developed HVRLocator, a publicly available tool that efficiently identifies the exact region sequenced by a set of 16S rRNA sequences, and can therefore greatly accelerate the identification of candidate datasets for reanalysis. The extensive validation of HVRLocator also highlights its potential for application towards data reuse.

Given the ubiquity of bacteria and their relevance to their environments, a wide range of disciplines employ 16S rRNA gene metabarcoding sequencing, and contribute data to INSDC archives in the process [38]. Indeed, according to available ENA metadata, metabarcoding datasets still dwarf metagenomic datasets by a factor of ten. Due to the lack of curation of INSDC metadata, information derived from peer-reviewed literature has been proposed as a central source of technical metadata that can enrich existing datasets [39], but the diversity of disciplines that employ metabarcoding also results in different degrees of resolution in the technical metadata provided for the sequence data. These metadata may lack the resolution necessary for an improved bioinformatics process or even introduce errors. Here, HVRLocator serves to bypass the need to return to the original literature to obtain the necessary processing metadata and the higher resolution information, such as the exact start and end sequence positions instead of the general region sequenced, and to correct potential errors that might be present in the literature-derived metadata.

To our knowledge, no tool has been designed with the specific purpose of automated metadata extraction from archived metabarcoding datasets to facilitate bioinformatics processing. Based on complete or near-complete 16S rRNA gene sequences and for a given set of primers, HyperEx (HyperVariable Region Extractor) [40] evaluates the precision and accuracy of different primer pairs in retrieving microbial diversity, and was developed as a tool for primer selection. Similarly, Qscore [9] evaluates the performance of 16S rRNA amplicons, assessing metrics including amplification rate, multitier taxonomic annotation, sequence type, and length. In contrast, HVRLocator does not rely on simulations or *a priori* primer information, and is designed for its application to existing datasets. HVRLocator operates directly on large, INSDC-archived metabarcoding datasets to identify the start and end positions of sequenced 16S rRNA amplicons, determine their corresponding hypervariable regions, and detect the presence primer sequences, generating the technical metadata that is needed for bioinformatics processing of the raw sequences. Additionally, some QIIME 2 plugins provide functionality that overlaps with certain steps of the HVRLocator workflow. For example, q2-cutadapt [41] uses cutadapt to remove adapter sequences, primers, and other unwanted sequences from high-throughput sequencing reads, thereby ensuring clean data for downstream analysis, but it requires prior knowledge of the exact primer or unwanted sequences used in the samples in order to accurately detect and remove them. Similarly, the quality-control filter-reads plugin [41] filters demultiplexed single- or paired-end sequences based on their alignment to a reference database using Bowtie 2 [42] and SAMtools [43] to remove contaminants (e.g., human DNA) or to retain only sequences that align to a specified reference, but also relies on alignment to external reference sequences and does not report hypervariable regions or primer information. USEARCH provides functions related to primer matching (e.g., search_oligodb, search_pcr, and search_pcr2) [44]. However, as with QIIME 2, users must supply the primer sequences in advance to perform database matching. In contrast, HVRLocator eliminates the need for prior knowledge of the exact primer sequences by automatically inferring this information.

Technical metadata is crucial, as species identification depends heavily on the targeted region and its length [12]. In the case of 16S rRNA gene metabarcoding, information about the gene region sequenced is essential for the bioinformatics processing of the sequence data, and for the statistical analyses (i.e., as a random effect in a hierarchical model) and downstream data interpretation. From an ecological perspective, the ability to consistently target the same genetic region across different studies brings us closer to achieving a macroecological understanding of microbial communities [8, 12, 45]. In this vein, HVRLocator can support and accelerate the bioinformatics processing of 16S rRNA metabarcoding data, enhancing comparability and improving short-read training sets for future predictive microbiome studies. Although the present work focuses on bacteria, we acknowledge that other domains of life, including Archaea and Eukaryotes, are relevant for future meta-analyses and synthesis studies. Consequently, future versions of the program aim to incorporate alternative model sequences representing Archaea and Eukaryote.

In the future, HVRLocator may support decision-making in the creation of large databases, improving the robustness and resolution of microbiome studies [13, 46, 47]. As long-read sequencing technologies gain relevance, HVRLocator could serve as a foundation for developing procedures to integrate multiple sequences with different coverage, ultimately enhancing our ability to capture microbial diversity more comprehensively.

## Availability of supporting source code and requirements

Project name: HVRLocator

Project home page: https://github.com/fbcorrea/HVRLocator

Operating system(s): Linux OS

Programming language: Python 3.9

Other requirements: Singularity container platform https://cloud.sylabs.io/library/jsaraiva/repo/hvrlocator

License: MIT license

RRID:SCR_027407

biotools: hvrlocator

## Supplementary Material

giag040_Supplemental_Files

giag040_Authors_Response_To_Reviewer_Comments_original_submission

giag040_Authors_Response_To_Reviewer_Comments_revision_1

giag040_GIGA-D-25-00344_original_submission

giag040_GIGA-D-25-00344_Revision_1

giag040_GIGA-D-25-00344_Revision_2

giag040_Reviewer_1_Report_original_submission Reviewer 1 -- 10/13/2025

giag040_Reviewer_1_Report_revision_1 Reviewer 1 -- 3/9/2026

giag040_Reviewer_2_Report_original_submission Reviewer 2 -- 12/6/2025

giag040_Reviewer_2_Report_revision_1 Reviewer 2 -- 3/12/2026

## Data Availability

The public datasets used in this paper can be found in Supplementary Tables S1, S2, S4, and S7. Machine learning annotations have been deposited in the DOME registry [48].

## Additional files

**Supplementary Table S1:** Run accession list with primers used for training and testing the random forest (RF) model.

**Supplementary Table S2:** Run accession list without primers used for training and testing the random forest (RF) model.

**Supplementary Table S3:** Random forest model for primer presence prediction.

**Supplementary Table S4:** Datasets (1–4) included in the validation process.

**Supplementary Table S5:** Number of samples per dataset and run time (in minutes) using 8 GB of RAM and 4 CPU cores.

**Supplementary Table S6:** Evaluation of tool performance on Archaeal and Eukaryotic sequences.

**Supplementary Table S7:** Run accession list for case study

**Supplementary Table S8:** Case study output.

**Supplementary Table S9:** Number of samples in which primer assignment matched the metadata (Figure S1.b)

**Supplementary Figure S1:** Variation in gene coverage across sequences and number of samples in which primer assignment matched the metadata.

## Abbreviations

ASV: Amplicon Sequence Variants; DDBJ: DNA Databank of Japan; DRA: Sequence Read Archives; EBI: European Bioinformatics Institute; ENA: European Nucleotide Agency; HPC: High Performance Computing; INSDC: International Nucleotide Sequence Database Collaboration; ITS: Internal Transcribed Spacer; NCBI: National Center for Biotechnology Information; NIG: National Institute of Genetics; SRA: Sequence Read Archive; SSU rRNA: Small Subunit Ribosomal RNA; TSV: Tab-separated values; UFZ: Helmholtz Center for Environmental Research.
