# Supplementary material for: HVRLocator: a computationally efficient tool for identifying hypervariable regions in large 16S rRNA datasets
Source: Gigascience. 2026 Apr 8;15:giag040. doi: 10.1093/gigascience/giag040 (PMC13188219; doi:10.1093/gigascience/giag040)

SUPPLEMENTARY MATERIAL

Figure S1. A) Variation in gene coverage across sequences, B) Number of samples in which primer assignment matched the metadata.

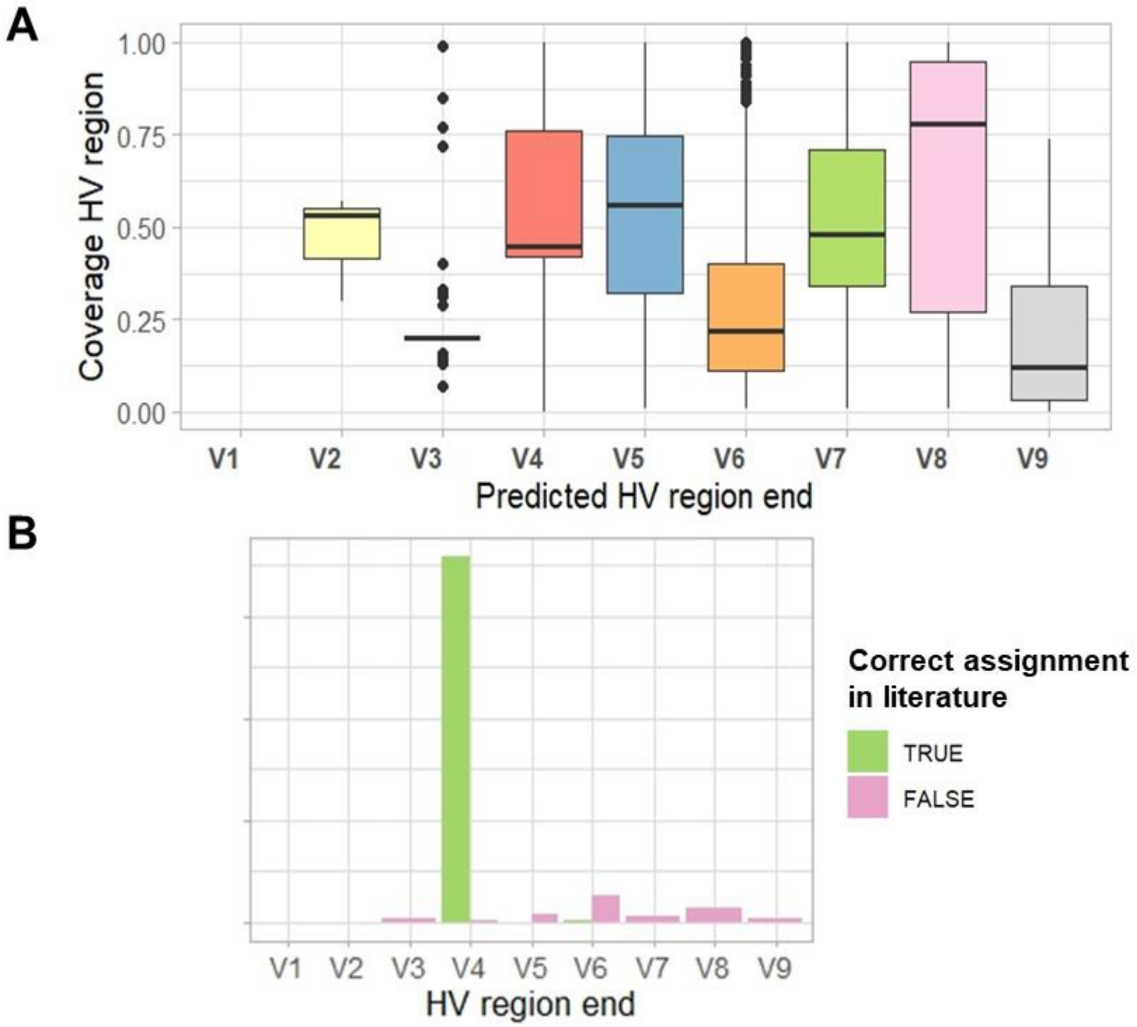

Supplement: giag040_Supplemental_Files [file giag040_supplemental_files.zip › Figure_S1.pdf]
