# Supplementary material for: HVRLocator: a computationally efficient tool for identifying hypervariable regions in large 16S rRNA datasets
Source: Gigascience. 2026 Apr 8;15:giag040. doi: 10.1093/gigascience/giag040 (PMC13188219; doi:10.1093/gigascience/giag040)
Supplement: giag040_Supplemental_Files [file giag040_supplemental_files.zip › TableS3_Random_Forest_Model_for_Primer_Presence_Prediction.pdf]

Table S3. Random Forest Model for Primer Presence Prediction

Purpose

The Random Forest (RF) model is designed to predict the presence of a primer in a given SRA sequencing dataset by analyzing the quality score distribution of the initial subset of reads.

Data Source

- A curated collection of SRA runs known to contain or lack specific primer sequences.
- Each SRA run was pre-processed with `fastp` to trim adapters and filter low-quality reads.
- From each processed sample, the **first 1000 reads** were extracted using `fastq-dump` with `-X 1000`.

Feature Engineering

Two sets of quality scores were extracted from each sample:

- **`s1`**: The quality scores from positions 1–5 of the first 1000 reads.
- **`s2`**: The quality scores from positions 6–10 of the same reads.

From each of these two segments, the following 8 statistical features were computed:

1. `count` – Number of quality scores extracted
2. `mean` – Average quality score
3. `median` – Median quality score
4. `std` – Standard deviation
5. `min` – Minimum value
6. `max` – Maximum value
7. `skew` – Skewness of the distribution (25th percentile)
8. `kurtosis` – Kurtosis of the distribution (75th percentile)

Total of **16 features** per sample:

- `1_5_count`, `1_5_mean`, ..., `1_5_kurtosis`
- `6_10_count`, `6_10_mean`, ..., `6_10_kurtosis`

Labels

Each sample was labeled as:

- **1** if the primer was confidently present
- **0** if the primer was confidently absent

Model Training

- **Algorithm**: RandomForestClassifier from `scikit-learn`
- **Version**: scikit-learn 1.2.1
- **Parameters**:
  - `n_estimators=100`
  - `random_state=42`
- **Training/Test Split**: 80/20 split with stratification
- **Scaler**: None (RF handles raw value ranges robustly)

Model Persistence

- The trained model was serialized using `joblib.dump(model, "rf_model.pkl")`
- Later used via `joblib.load("rf_model.pkl")` within the HVRegLocator script

Usage in Pipeline

- When the `--model` flag is passed, quality scores are extracted from trimmed FASTQ reads
- Features are computed and passed to the loaded model
- Prediction is stored as:
  - `Primer_Presence`: "Yes" / "No"
  - `Score_Primer_Presence`: Probability score from `predict_proba()`

Version Compatibility Note

The model was trained with **scikit-learn 1.2.1**, and should ideally be used in the same version to avoid `InconsistentVersionWarning` during unpickling.

Model Accuracy: 99.96%

Precision:

- No primers: 99.96%
- With primers: 100%

Recall:

- No primers: 100%
- With primers: 99.55%
