# Supplementary material for: HVRLocator: a computationally efficient tool for identifying hypervariable regions in large 16S rRNA datasets
Source: Gigascience. 2026 Apr 8;15:giag040. doi: 10.1093/gigascience/giag040 (PMC13188219; doi:10.1093/gigascience/giag040)
Supplement: giag040_Supplemental_Files [file giag040_supplemental_files.zip › TableS5_RunTime.pdf]

SUPPLEMENTARY MATERIAL

Table S5: Number of samples per dataset and run time (in minutes) using 8 GB of RAM and 4 CPU cores. We selected samples from the Earth Microbiome Project (Dataset 1), MiCoDa V1 (Dataset 2), and Datathon activities (Dataset 4). All samples were downloaded from the NCBI.

Dataset	Threshold	Duration (minutes)	Total Samples (Run Accession Numbers)	Samples processed successfully	Samples Not Processed (Warnings)	Time to process 1 sample	Samples processed per minute
**1**	**0.5**	2078.26	17537	16059	1478	0.1185	7.7271
**2**	**0.5**	2746.85	18426	16771	1655	0.1491	6.1055
**4**	**0.5**	922.38	5308	5163	145	0.1738	5.5975
**1**	**0.6**	2106.22	17537	16059	1478	0.1201	7.6245
**2**	**0.6**	2746.85	18426	16771	1655	0.1491	6.1055
**4**	**0.6**	904.98	5308	5163	145	0.1705	5.7051
**1**	**0.7**	2114.30	17537	16059	1478	0.1206	7.5954
**2**	**0.7**	2759.60	18426	16771	1655	0.1498	6.0773
**4**	**0.7**	917.05	5308	5163	145	0.1728	5.6300
