# Supplementary material for: HVRLocator: a computationally efficient tool for identifying hypervariable regions in large 16S rRNA datasets
Source: Gigascience. 2026 Apr 8;15:giag040. doi: 10.1093/gigascience/giag040 (PMC13188219; doi:10.1093/gigascience/giag040)
Supplement: giag040_Supplemental_Files [file giag040_supplemental_files.zip › TableS6_Tool_performance_on_Archaeal_Eukaryotic_sequences.pdf]

SUPPLEMENTARY MATERIAL

Table S6: Evaluation of tool performance on Archaeal and Eukaryotic sequences

Even for bacterial taxa that are highly divergent from E. coli, the 16S rRNA gene remains highly conserved across Bacteria, ensuring sufficient sequence similarity for reliable alignment to the appropriate reference region. Although 16S rRNA sequences vary in length among taxa, our validation showed 99% agreement between the predicted hypervariable regions and those reported in the original publications (metadata or NCBI records). Together, the conserved structure of the 16S rRNA gene and the strong empirical validation support the conclusion that the current alignment strategy provides reliable estimates of start and end positions for hypervariable regions in Bacteria and adequately addresses the objectives of this study.

Regarding Archaea and Eukaryotes, we performed several tests and identified potential warning signs that may alert users to review the metadata of sequences exhibiting the following patterns:

1. Putative ITS region: Sequences showing coverage across more than four or five hypervariable (HV) regions. In such cases, users should verify the following information in the metadata: the sequencing platform. If the platform used for these sequences is not PacBio or Nanopore, the primers may be targeting regions that are not specific to bacteria. If the mentioned platforms were used, users should also verify that the primers are not targeting Archaea or Eukaryotes.

The following table presents examples of ITS regions analyzed using HVRLocator:

Sample_ID	Primer Presence	Score Primer Presence	Min Alignment start	Max Alignment end	Average Alignment start	Average Alignment end	Median Alignment start	Median Alignment end	Predicted HV region start	Predicted HV region end	Coverage based HV region start	Coverage based HV region end	Coverage HV region start	Coverage HV region end	Warnings
SRR24285816	FALSE	0.17	0	1540	374	1068	399	1148	V3	V7	V4	V7	0.45	0.72	V3 below threshold of 0.5
SRR24285817	FALSE	0.17	0	1540	398	992	399	1110	V3	V7	V4	V6	0.45	0.39	V3 and V7 below threshold of 0.5
SRR24285818	FALSE	0.17	0	1540	400	1018	399	1110	V3	V7	V4	V6	0.45	0.39	V3 and V7 below threshold of 0.5
SRR24285819	FALSE	0.17	0	1540	402	994	399	1110	V3	V7	V4	V6	0.45	0.39	V3 and V7 below threshold of 0.5
SRR24285820	FALSE	0.17	0	1540	446	1017	399	1110	V3	V7	V4	V6	0.45	0.39	V3 and V7 below threshold of 0.5
SRR24285821	FALSE	0.16	0	1540	431	1000	399	1110	V3	V7	V4	V6	0.45	0.39	V3 and V7 below threshold of 0.5
SRR24285822	FALSE	0.17	0	1540	374	953	399	1110	V3	V7	V4	V6	0.45	0.39	V3 and V7 below threshold of 0.5
SRR24285823	FALSE	0.17	0	1540	392	961	399	1010	V3	V6	V4	V6	0.45	0.69	V3 below threshold of 0.5
SRR24285824	FALSE	0.17	0	1540	392	968	399	953	V3	V6	V4	V5	0.45	0.38	V3 and V6 below threshold of 0.5
SRR24285825	FALSE	0.17	0	1540	401	963	399	953	V3	V6	V4	V5	0.45	0.38	V3 and V6 below threshold of 0.5
SRR25665816	FALSE	0.17	0	1540	357	872	186	826	V2	V5	V2	V4	0.56	0.2	V5 below threshold of 0.5
SRR25665819	FALSE	0.2	0	1540	690	1172	903	1450	V6	V9	V6	V8	0.91	0.34	V9 below threshold of 0.5
SRR25665821	FALSE	0.22	0	1540	425	956	186	826	V2	V5	V2	V4	0.56	0.2	V5 below threshold of 0.5
SRR25665824	FALSE	0.2	0	1540	627	1210	685	1210	V4	V8	V5	V7	0.38	0.16	V4 and V8 below threshold of 0.5
SRR25665826	FALSE	0.22	0	1540	602	1118	574	1048	V4	V6	V4	V6	0.72	0.91	NA
SRR25665828	FALSE	0.2	0	1540	303	1164	177	1096	V2	V7	V2	V6	0.61	0.27	V7 below threshold of 0.5
SRR25665830	FALSE	0.17	0	1540	600	1149	529	1048	V4	V6	V4	V6	0.85	0.91	NA
SRR25665832	FALSE	0.21	0	1540	449	970	186	826	V2	V5	V2	V4	0.56	0.2	V5 below threshold of 0.5

ERR5924913	FALSE	0.17	0	1540	482	1055	548	1111	V4	V7	V4	V6	0.8	0.4	V7 below threshold of 0.5
ERR5924916	FALSE	0.17	0	1540	463	1076	595	1177	V4	V7	V4	V7	0.65	0.97	NA

2. Putative Archaea or 18S hypervariable region: As clarified in the manuscript, users must ensure that the target domain is Bacteria. Due to similarities in gene length and conserved regions, sequences targeting archaeal 16S rRNA genes or eukaryotic 18S rRNA genes may still produce a prediction when analyzed with HVRLocator. However, the tool is specifically designed and validated for bacterial 16S rRNA sequences. Therefore, verification of the sample metadata is essential to confirm that the predicted hypervariable region corresponds to bacterial sequences.
