# Supplementary material for: HVRLocator: a computationally efficient tool for identifying hypervariable regions in large 16S rRNA datasets
Source: Gigascience. 2026 Apr 8;15:giag040. doi: 10.1093/gigascience/giag040 (PMC13188219; doi:10.1093/gigascience/giag040)
Supplement: giag040_Supplemental_Files [file giag040_supplemental_files.zip › TableS8_CaseStudyOutput.pdf]

SUPPLEMENTARY MATERIAL

Table S8: Case study outputs

The average alignment column showed that 77.5% of the samples (32,677) were within the expected range for the V4 region start position 501-550 bp (**Supplementary Table S7a**), while the end position of these specific samples varied from 601 to 1500 bp; 45.8% of the sequences ended between 651 and 700 bp, whereas 30.8% ended between 751 and 800 bp (**Supplementary Table S7b**).

A. Average alignment start/end

Average alignment start			Average alignment end		
**Range**	**Number of samples**	**Percentage**	**Range**	**Number of samples**	**Percentage**
501-550	32677	77.50	651-700	15414	36.56
301-350	2528	6.00	751-800	10908	25.87
551-600	1707	4.05	801-850	5595	13.27
451-500	1033	2.45	601-650	2773	6.58
351-400	772	1.83	901-950	1841	4.37
601-650	680	1.61	701-750	854	2.03
401-450	398	0.94	1051-1100	607	1.44
1-50	387	0.92	851-900	525	1.25
651-700	367	0.87	1301-1350	523	1.24
201-250	353	0.84	951-1000	513	1.22
151-200	279	0.66	1001-1050	506	1.20
251-300	229	0.54	1251-1300	439	1.04
751-800	205	0.49	1101-1150	364	0.86
701-750	118	0.28	1151-1200	346	0.82
951-1000	113	0.27	301-350	340	0.81
801-850	94	0.22	1201-1250	303	0.72
101-150	86	0.20	501-550	121	0.29
-49-0	43	0.10	1351-1400	61	0.14
51-100	40	0.09	551-600	57	0.14
851-900	26	0.06	1401-1450	49	0.12
901-950	16	0.04	451-500	17	0.04
1101-1150	9	0.02	1451-1500	6	0.01
1001-1050	3	0.01	201-250	1	0.00
1051-1100	2	0.00	251-300	1	0.00
1151-1200	1	0.00	351-400	1	0.00
			401-450	1	0.00
**TOTAL**	**42166**	**100.00**	**TOTAL**	**42166**	**100.00**

B. Average alignment end of the previous 32677 sequences with average alignment start position at 501-550 bp.

Average alignment end		
**Range**	**Number of samples**	**Percentage**
651-700	14975	45.83
751-800	10081	30.85
601-650	2712	8.30
801-850	2651	8.11
901-950	1287	3.94
701-750	372	1.14
1001-1050	155	0.47
851-900	126	0.39
951-1000	82	0.25
1051-1100	68	0.21
1201-1250	46	0.14
1151-1200	42	0.13
1251-1300	27	0.08
1301-1350	20	0.06
1101-1150	19	0.06
1351-1400	10	0.03
1401-1450	2	0.01
1451-1500	2	0.01
**TOTAL**	**32677**	**100.00**

The results of the median alignment columns showed the start position 500-550 bp for a total of 34,474 samples (81.8%) (**Supplementary Table S7c**). These samples had an end position from 601 to 1550 bp, with 47.8% of the sequences ending from 651-700 bp and 31.3% ending from 751-800 bp (**Supplementary Table S7d**).

C. Median alignment start/end

Median alignment start			Median alignment end		
**Range**	**Number of samples**	**Percentage**	**Range**	**Number of samples**	**Percentage**
501-550	34474	81.76	651-700	16736	39.69
301-350	2699	6.40	751-800	11041	26.18
451-500	616	1.46	801-850	5300	12.57
351-400	532	1.26	601-650	2921	6.93
1-50	476	1.13	901-950	1863	4.42
401-450	455	1.08	1351-1400	783	1.86
601-650	439	1.04	851-900	493	1.17
551-600	374	0.89	1101-1150	350	0.83
151-200	323	0.77	951-1000	349	0.83
201-250	301	0.71	301-350	345	0.82
701-750	252	0.60	1201-1250	304	0.72
101-150	215	0.51	1051-1100	272	0.65
751-800	213	0.51	1151-1200	269	0.64
651-700	176	0.42	1301-1350	234	0.55
951-1000	154	0.37	1001-1050	212	0.50
251-300	103	0.24	1251-1300	170	0.40
801-850	98	0.23	501-550	155	0.37
51-100	92	0.22	701-750	111	0.26
-49-0	67	0.16	1401-1450	93	0.22
1051-1100	37	0.09	1451-1500	79	0.19
1001-1050	19	0.05	551-600	31	0.07
1101-1150	19	0.05	351-400	18	0.04
901-950	18	0.04	1501-1550	15	0.04
851-900	11	0.03	401-450	13	0.03
1151-1200	1	0.00	451-500	6	0.01
1301-1350	1	0.00	201-250	2	0.00
1401-1450	1	0.00	151-200	1	0.00
**TOTAL**	**42166**	**100**	**TOTAL**	**42166**	**100**

D. Median alignment end of the previous 34474 sequences with average alignment start position at 501-550 bp.

Median alignment end		
**Range**	**Number of samples**	**Percentage**
651-700	16483	47.81
751-800	10782	31.28
601-650	2828	8.20
801-850	2478	7.19
901-950	1418	4.11
851-900	178	0.52
701-750	72	0.21
1001-1050	43	0.12
1151-1200	40	0.12
951-1000	37	0.11
1201-1250	28	0.08
1351-1400	26	0.08
1051-1100	20	0.06
1101-1150	17	0.05
1301-1350	11	0.03
1251-1300	6	0.02
1401-1450	3	0.01
1451-1500	3	0.01
1501-1550	1	0.00
**TOTAL**	**34474**	**100**

E. Predicted HV region start/end

Predicted HV region start			Predicted HV region end		
**HV region**	**Number of samples**	**%**	**HV region**	**Number of samples**	**%**
V1	626	1.48	V1	0	0.00
V2	951	2.26	V2	3	0.01
V3	4088	9.70	V3	380	0.90
V4	36217	85.89	V4	35959	85.28
V5	29	0.07	V5	692	1.64
V6	196	0.46	V6	2733	6.48
V7	56	0.13	V7	578	1.37
V8	2	0.00	V8	1446	3.43
V9	1	0.00	V9	375	0.89
**TOTAL**	**42166**	**100.00**	**TOTAL**	**42166**	**100.00**

In the coverage-based start of the HV region (Figure 4c), 36,130 samples began in the V4 region, with a median coverage of 0.57.

F. Coverage based HV region start/end

Coverage based HV region start			Coverage based HV region end		
HV region	Number of samples	%	HV region	Number of samples	%
V1	517	1.23	V1	2	0.00
V2	496	1.18	V2	363	0.86
V3	3759	8.91	V3	478	1.13
V4	36130	85.69	V4	35836	84.99
V5	1004	2.38	V5	2600	6.17
V6	61	0.14	V6	865	2.05
V7	178	0.42	V7	803	1.90
V8	19	0.05	V8	1206	2.86
V9	2	0.00	V9	13	0.03
**TOTAL**	**42166**	**100.00**	**TOTAL**	**42166**	**100.00**

G. Coverage based HV region end of the previous 36130 sequences with average alignment start position at 501-550 bp.

Coverage based HV region end		
HV region	Number of samples	%
V4	33022	91.40
V5	2079	5.75
V6	505	1.40
V7	287	0.79
V8	236	0.65
V9	1	0.00
**TOTAL**	**36130**	**100.00**

H. Mean coverage associated with the most frequent warning during the samples processing.

Average of coverage HV region start									
Warnings	Cov_V1	Cov_V2	Cov_V3	Cov_V4	Cov_V5	Cov_V6	Cov_V7	Cov_V8	Cov_V9
V1 and V2 below threshold of .6	0.45	0.42							
V1 and V3 below threshold of .6	0.36	1.00	0.35						
V1 and V4 below threshold of .6	0.34	1.00	1.00	0.35					
V1 and V5 below threshold of .6	0.37	1.00	1.00	1.00	0.31				
V1 and V6 below threshold of .6	0.30	1.00	1.00	1.00	1.00	0.33			
V1 and V8 below threshold of .6	0.41	1.00	1.00	1.00	1.00	1.00	1.00	0.23	
V1 below threshold of .6	0.39	1.00	0.95	0.73	0.65	0.65	0.65	0.58	
V2 and V3 below threshold of .6		0.43	0.26						
V2 and V4 below threshold of .6		0.38	1.00	0.45					
V2 and V5 below threshold of .6		0.21	1.00	1.00	0.28				
V2 and V6 below threshold of .6		0.25	1.00	1.00	1.00	0.26			
V2 and V7 below threshold of .6		0.28	1.00	1.00	1.00	1.00	0.32		
V2 and V8 below threshold of .6		0.39	1.00	1.00	1.00	1.00	1.00	0.27	
V2 and V9 below threshold of .6		0.44	1.00	1.00	1.00	1.00	1.00	1.00	0.47
V2 below threshold of .6	0.28	0.47	1.00	0.99	0.91	0.88	0.78	0.67	
V3 and V4 below threshold of .6			0.40	0.38					
V3 and V5 below threshold of .6			0.28	1.00	0.43				
V3 and V6 below threshold of .6			0.17	1.00	1.00	0.39			
V3 and V7 below threshold of .6			0.37	1.00	1.00	1.00	0.37		
V3 and V8 below threshold of .6			0.35	1.00	1.00	1.00	1.00	0.29	
V3 and V9 below threshold of .6			0.19	1.00	1.00	1.00	1.00	1.00	0.16
V3 below threshold of .6	0.35	0.48	0.21	0.56	0.39	0.38	0.11	0.73	
V4 and V5 below threshold of .6				0.46	0.43				
V4 and V6 below threshold of .6				0.39	1.00	0.14			
V4 and V7 below threshold of .6				0.28	1.00	1.00	0.39		
V4 and V8 below threshold of .6				0.18	1.00	1.00	1.00	0.18	
V4 and V9 below threshold of .6				0.26	1.00	1.00	1.00	1.00	0.23
V4 below threshold of .6	0.35	0.47	0.16	0.41	0.20	0.14	0.85	0.60	0.27
V5 and V6 below threshold of .6					0.33	0.17			
V5 and V8 below threshold of .6					0.24	1.00	1.00	0.59	
V5 and V9 below threshold of .6					0.43	1.00	1.00	1.00	0.20
V5 below threshold of .6	0.16	0.18	0.35	0.97	0.25	0.40	0.40	0.25	
V6 and V8 below threshold of .6						0.47	1.00	0.18	
V6 and V9 below threshold of .6						0.52	1.00	1.00	0.21
V6 below threshold of .6	0.14	0.30	0.67	0.86	0.98	0.18	0.65	0.32	0.83
V7 and V8 below threshold of .6							0.45	0.35	
V7 and V9 below threshold of .6							0.57	1.00	0.27
V7 below threshold of .6			0.47	0.76	0.97	0.97	0.29	0.23	0.12
V8 below threshold of .6	0.53	0.25	0.31	0.77	0.94	0.97	0.99	0.32	0.33
V9 below threshold of .6	0.43	0.22	0.32	0.76	0.87	0.96	0.99	0.99	0.29
