# Supplementary material for: HVRLocator: a computationally efficient tool for identifying hypervariable regions in large 16S rRNA datasets
Source: Gigascience. 2026 Apr 8;15:giag040. doi: 10.1093/gigascience/giag040 (PMC13188219; doi:10.1093/gigascience/giag040)
Supplement: giag040_GIGA-D-25-00344_Revision_1 [file giag040_giga-d-25-00344_revision_1.pdf]

GigaScience

HVRLocator: A Computationally Efficient Tool for Identifying Hypervariable Regions in Large 16S rRNA Datasets

--Manuscript Draft--

**Manuscript Number:**	GIGA-D-25-00344R1
**Full Title:**	HVRLocator: A Computationally Efficient Tool for Identifying Hypervariable Regions in Large 16S rRNA Datasets
**Article Type:**	Technical Note
**Funding Information:**	German Centre for Integrative Biodiversity Research (iDiv) (sIBTEDS project (Illuminating Blindspots Through Equitable Data Reuse practices in the Global South)) — Dr Stephanie D. Jurburg
**Abstract:**	Background: Metabarcoding of the 16S rRNA gene is widely used to assess microbial diversity due to its cost-effectiveness and efficiency. However, publicly available 16S rRNA metabarcoding datasets often lack standardized metadata, particularly information on the sequenced hypervariable regions or primers used, which are critical to their accurate reuse. To address this, we present HVRLocator, a computational tool that (1) identifies the start and end positions of 16S rRNA amplicons, (2) determines their corresponding hypervariable regions, and (3) detects the presence of primer sequences. This tool was validated on four datasets comprising 41,513 samples generated with different primers and sequencing platforms.<br><br>Results: HVRLocator can process archived 16S rRNA sequences from NCBI SRA at an average rate of 6.5 samples per minute. Validation showed it reliably detects amplicon start and end positions across datasets sequenced with different primers and platforms, achieving 100% accuracy within single-platform studies and correctly revealing length heterogeneity across platforms. It also flagged misannotated metadata and problematic sequences, underscoring its value as a sequence data curation tool. Finally, HVRLocator can select comparable sequences to build large 16S rRNA amplicon databases spanning the same hypervariable region, facilitating cross-study comparisons.<br><br>Conclusion: HVRLocator overcomes unreliable metadata by accurately identifying 16S rRNA amplicon start and end positions, determining hypervariable regions, and detecting primer sequences, enabling accurate curation and large-scale processing of 16S rRNA data for reliable and reproducible microbial studies, syntheses, and meta-analyses.
**Corresponding Author:**	Clara Maria Arboleda-Baena, Ph.D.<br>iDiv: German Centre for Integrative Biodiversity Research (iDiv) Halle-Jena-Leipzig<br>Leipzig, GERMANY
**Corresponding Author Secondary Information:**	
**Corresponding Author's Institution:**	iDiv: German Centre for Integrative Biodiversity Research (iDiv) Halle-Jena-Leipzig
**Corresponding Author's Secondary Institution:**	
**First Author:**	Clara Arboleda-Baena, Ph.D.
**First Author Secondary Information:**	
**Order of Authors:**	Clara Arboleda-Baena, Ph.D.
	Felipe Borim Correa
	Joao Pedro Saraiva
	Santiago Castillo-Rivadeneira
	Jonas Coelho Kasmanas

Powered by Editorial Manager® and ProduXion Manager® from Aries Systems Corporation

	Antonis Chatzinotas
	Stephanie D. Jurburg
**Order of Authors Secondary Information:**	
**Response to Reviewers:**	GIGA-D-25-00344<br>HVRLocator: A Computationally Efficient Tool for Identifying Hypervariable Regions in Large 16S rRNA Datasets<br>Clara Arboleda-Baena; Felipe Borim Correa; Joao Pedro Saraiva; Santiago Castillo-Rivadeneira; Jonas Coelho Kasmanas; Antonis Chatzinotas; Stephanie D. Jurburg<br>GigaScience<br><br>Leipzig, 17 February 2026<br><br>Dear Dongni Ma,<br><br>I hope this message finds you well.<br><br>Please find attached our response to the reviewers regarding manuscript GIGA-D-25-00344: HVRLocator: A Computationally Efficient Tool for Identifying Hypervariable Regions in Large 16S rRNA Datasets. We have addressed all comments. We would like to thank you and the reviewers for the constructive feedback, which has helped us improve the manuscript and clarify our message. We hope that the revisions satisfactorily address the minor revision requests.<br><br>As outlined in the response document, it was not necessary to register a new software application in the bio.tools or SciCrunch.org databases to obtain RRID (Research Resource Identification Initiative ID) or biotoolsID identifiers.<br><br>We have now also uploaded the scripts used in our analyses to GitHub, as suggested by one of the reviewers.<br><br>We look forward to your response.<br><br>Best wishes,<br><br>Dr. Clara Arboleda<br>On behalf of all co-authors<br><br>Reviewer reports:<br><br>Reviewer #1: Metabarcoding data are accumulating rapidly. This paper makes a very valuable contribution to the automated extraction and curation of metabarcoding data and should be of great value in facilitating the re-use of existing data and the construction of custom databases based on these.<br>I have not tested or tried to install the software myself, as the manuscript provided sufficient detail to enable me to assess the tool<br><br>R/ We thank the reviewer for their positive feedback.<br><br>General comments:<br><br>The manuscript is written entirely in terms of "bacteria" and aligns amplicons to an E. coli model sequence. This is reasonable, but there should certainly be some

acknowledgement of Archaea and ideally some mention of Eukaryotes too. These are probably things for the discussion section of this manuscript, but the authors may wish to consider whether a future version of the program could contain options to use model Archaea and Eukaryote sequences as alternatives to the E. coli model.

R/ Thank you for the comment. We acknowledge that this software may be useful for researchers working with metabarcoding data from other realms of life. This is foreseen by the software, which allows the user to input their own reference sequence and map against it, offering that flexibility. We now refer to this in Lines 389-393. While this manuscript focuses on the evaluation of E. coli for usage against 16S rRNA gene data, we are currently benchmarking its use for fungi (ITS region) for future versions, although this is beyond the scope of the present work. For clarity, we have added the following text to the manuscript:

Lines 389-393: “Although the present work focuses on bacteria, we acknowledge that other domains of life, including Archaea and Eukaryotes, are relevant for future meta-analyses and synthesis studies. Consequently, future versions of the program aim to incorporate alternative model sequences representing Archaea and Eukaryota.”

It would also be helpful to assess how the program with its E. coli model deals with sequence data from Archaea, Eukaryotes (including mitochondria) and bacteria that are very divergent from E. coli.

R/ Thank you for the comment. Even for bacterial taxa that are highly divergent from E. coli, the 16S rRNA gene remains highly conserved across Bacteria, ensuring sufficient sequence similarity for reliable alignment to the appropriate reference region. Although 16S rRNA sequences vary in length among taxa, our validation showed 99% agreement between the predicted hypervariable regions and those reported in the original publications (metadata or NCBI records). Together, the conserved structure of the 16S rRNA gene and the strong empirical validation support the conclusion that the current alignment strategy provides reliable estimates of start and end positions for hypervariable regions in Bacteria and adequately addresses the objectives of this study.

Regarding Archaea and Eukaryotes, we performed several tests and identified potential warning signs that may alert users to review the metadata of sequences exhibiting the following patterns:

1. Putative ITS region: Sequences showing coverage across more than four or five hypervariable (HV) regions. In such cases, users should verify the following information in the metadata: the sequencing platform. If the platform used for these sequences is not PacBio or Nanopore, the primers may be targeting regions that are not specific to bacteria. If the mentioned platforms were used, users should also verify that the primers are not targeting Archaea or Eukaryotes.

The following table presents examples of ITS regions analyzed using HVRLocator:

2. Putative Archaea or 18S hypervariable region: As clarified in the manuscript, users must ensure that the target domain is Bacteria. Due to similarities in gene length and conserved regions, sequences targeting archaeal 16S rRNA genes or eukaryotic 18S rRNA genes may still produce a prediction when analyzed with HVRLocator. However, the tool is specifically designed and validated for bacterial 16S rRNA sequences. Therefore, verification of the sample metadata is essential to confirm that the predicted hypervariable region corresponds to bacterial sequences.

Finally, we have included the following statement in the main text to clarify this point for users:

Lines 275-283: “Importantly, validation highlighted HVRLocator tool’s ability to identify problematic sequences. For example, we observed 932 samples with abnormally long average sequence lengths (i.e., >600 bp) that exceeded the expected output lengths with Illumina platforms. Upon manually reviewing these sequences, we found that either the sequencing platform was incorrectly annotated in the metadata (NCBI or the

Powered by Editorial Manager® and ProduXion Manager® from Aries Systems Corporation

associated publication), or the sequences did not correspond to the 16S rRNA gene but rather to the Internal Transcribed Spacer (ITS) region or the nifH gene. This highlights the use of HVRLocator as a curation tool for large datasets, where human errors in annotation can significantly impact downstream analysis."

Lines 389-393: "Although the present work focuses on bacteria, we acknowledge that other domains of life, including Archaea and Eukaryotes, are relevant for future meta-analyses and synthesis studies. Consequently, future versions of the program aim to incorporate alternative model sequences representing Archaea and Eukaryota"

The methods section does not contain details of software used to generate the figures, or whether these figures are produced by "the pipeline" or by separate analysis of the .txt file that the pipeline produces. I suspect that it is that latter, in which case making the authors should make the scripts used available - as well as providing complete documentation of what has been done, this is likely to increase use made of the tool. And it would be helpful to include an output file in the supplementary materials.

R/ Thank you for the recommendation. We have now made all scripts and tables available on GitHub. The following text has been included in the Methods section:

"All graphics were carried out in R with RStudio interface (R Core Team, 2016), and all pipelines are available at: https://github.com/ClarArboledaBaena/HVRLocator-Figures."

Specific comments

Line 64 "however the integration of these data in light of processing metadata" - not clear

R/ Thank you for noting this. The correct phrase is: "however, the integration of these data in the context of Big Data processing and synthesis".

Line 67-8 "though bacterial diversity increases linearly with amplicon length".
Needs re-wording. The number of ASVs will increase with amplicon length, but the actual bacterial diversity in a sample is constant.

R/ Thank you for your comment. We have modified it to "though detected bacterial diversity increases linearly with amplicon length".

Line 79 "Wasimuddin and colleagues" should be "Wasimuddin et al". More generally, check that citations conform with journal house style

R/ Done

Line 79-82 "For example, Wasimuddin and colleagues [8] found that compared to three other primer sets targeting different regions, the primer pair targeting the V4 hypervariable region of the 16S rRNA gene produced the highest estimates of species richness and diversity across various sample types"
There are three issues here:
1) different primer pairs vary in their coverage and bias, so different primers targeting the same variable region will produce different numbers of ASVs
2) Even with complete coverage and the absence of bias, different variable regions will generate different numbers of ASVs as a result of differences in length and rate of evolution between variable regions (and differences in the number of ASVs that are clustered into OTUs at a particular sequence similarity threshold
3) The relationship between ASVs or OTUs and "species" is not straightforward (Edgar, 2018).
At minimum "species" should be replace with ASV or OTU (whichever Wasimuddin et al used)
Edgar, R. C. (2018). Updating the 97% identity threshold for 16S ribosomal RNA OTUs. Bioinformatics, 34(14), 2371-2375. doi:10.1093/bioinformatics/bty113

R/ Yes, the reviewer is correct regarding all three points. Accordingly, we have replaced the term "species" with "Amplicon Sequence Variants (ASVs)" and added the following sentence:

Powered by Editorial Manager® and ProduXion Manager® from Aries Systems Corporation

Lines 83-85: “However, different primers targeting the same variable region can still generate different numbers of ASVs (Parada et al., 2016)”

Line 89-90 "as bacterial diversity and taxonomic resolution linearly increase with target sequence length [12]." Overlaps with statements made in line 67-8, and the same issue applies here.

R/ Thank you for pointing this out. We agree that this statement overlaps with the one in lines 67–68. To avoid redundancy, we have rephrased lines 89–90 to better differentiate the concepts and improve clarity:

“Variation in amplicon length complicates the reuse of 16S rRNA metabarcoding data, since longer sequences tend to detect greater bacterial diversity and taxonomic resolution”

Lines 167-170. The output file contains (amongst other things) "Predicted HV region Start/End: Predicted hypervariable (HV) region based on the median alignment start and end positions across all reads, inferred from literature on conserved and hypervariable regions of the 16S rRNA gene (Brosius et al., 1978; Yang et al., 2016)". This implies that the program predicts a single variable region for each study. I am not clear what this column will contain for amplicons that contain more than one variable region, although columns 11-19 indicate that the program identifies the presence/absence of each of the 9 HV regions.

R/ Thank you very much for this helpful comment. For clarification, HVRLocator predicts a single hypervariable region for each sample, rather than for each study, as mentioned by the reviewer. As correctly pointed out, some studies (BioProjects) include samples spanning multiple hypervariable regions, a situation that has become increasingly common with the advent of long-read sequencing technologies.

In addition to identifying the hypervariable region corresponding to the average and median start and end positions of the amplicons within each sample, HVRLocator also reports the coverage across all nine hypervariable regions for every individual sample. In this way, users can evaluate the amplicon length, its exact start and end positions, and the coverage of specific hypervariable region(s) within each sample.

We have added the following sentence in the main manuscript to clarify this point for users (Lines 201-205): ”Finally, in addition to identifying the hypervariable region based on the average and median start and end positions of the amplicons within each sample, HVRLocator also reports coverage across all nine hypervariable regions for every individual sample. This allows users to assess the amplicon length, determine its exact start and end positions, and evaluate the coverage of specific hypervariable region(s) within each sample.”

Regarding studies that include samples with different hypervariable region coverage, we aimed to illustrate this scenario using Dataset B, which comprises 242 samples from two studies that compared different primer sets but were sequenced on the same platform (Wasimuddin et al., 2020; Varliero et al., 2023). As shown in Figure 2B, HVRLocator successfully predicts different variable regions within the same study.

My guess is that the authors are using "HV region" in two different sense:
1) Its usual meaning of one region out of V1 to V9
2) The sequence from the beginning of the first of the nine variable regions the amplicon includes to the end of the last.
It would also be helpful to indicate whether the sequence positions here are relative to the E coli model or refer to sequence positions in the amplicon

R/ Thank you for the comment. For clarification, we use the term “HV region” according to option (1), that is, its standard meaning as one of the regions V1–V9. To address the reviewer’s concern, we will provide an example illustrating the HVRLocator output and demonstrate how the information produced by the tool resolves the points raised:

The following table shows the HVRLocator output for five samples from the same study

	(Varliero et al., 2023). As shown, even within the same study, the tool predicts different HV regions for different samples.<br><br>Output (only 8 columns shown):<br><br>We are going to explain the first sample:<br><br>For sample ERR10042760, the average and median alignment start positions are 8 and 7, respectively. These values represent the sequence start positions relative to the E. coli reference model, as mentioned in your comment. As you can see the predicted HV region where this amplicon starts for this sample is V1.<br>The average and median alignment end positions are 537 and 534, respectively. These values indicate the sequence end positions relative to the E. coli reference model. The predicted hypervariable (HV) region at which this amplicon ends is V4.<br><br>This indicates that the samples cover HV regions V1, V2, V3, and V4. This can be verified by examining the remaining columns of the output.<br><br>Output (only 14 columns shown):<br><br>The tool further shows that the amplicon provides full coverage of HV regions V1 to V3. This is reflected in the columns "Cov_V1" to "Cov_V9," which report coverage values (ranging from 0 to 1) for each HV region. Coverage values close to or equal to 1 are observed for HV regions V1–V3, whereas a lower value is observed for V4.<br><br>This indicates that sample ERR10042760 starts in HV region V1 and ends in HV region V4, fully covering HV regions V1–V3, while HV region V4 is only partially covered. This information allows users to interpret the output and make informed decisions in downstream analysis pipelines.<br><br>Reviewer #2: General Comments<br>This manuscript introduces a tool named HVRLocator, designed to address the issue of missing or non-standard metadata in 16S rRNA sequencing data found in public databases such as the SRA. The tool identifies amplicon regions by aligning sequences to a reference genome and attempts to detect the presence of primers

Powered by Editorial Manager® and ProduXion Manager® from Aries Systems Corporation

	using a machine learning model. This is a subject with significant practical value, particularly for conducting large-scale meta-analyses. However, there are still many issues regarding methodological rigor, the depth of validation, and comparisons with existing tools that require further clarification by the authors.<br><br>R/ We address the reviewer’s concerns regarding methodological rigor, the depth of validation, and comparisons with existing tools in the following comments.<br><br>Major Comments<br><br>1. Concerns regarding the singularity of the reference sequence<br>A.The authors mention aligning sequences to a single Escherichia coli (J01859.1) reference genome to determine start and end positions. Is a single E. coli reference sufficient to cover Archaea or bacterial phyla that are distantly related to Proteobacteria, which may be present in environmental samples (e.g., soil, ocean)?<br><br>R/ HVRLocator aims to identify primers designed to target Bacteria only, not Archaea, although We acknowledge that bacterial primers amplify non-target 16S rRNA from Archaea. Crucially, this software is designed to work with metabarcoding data, where the composition of the sequenced community is usually unknown a priori, so it is possible and expected that when distantly-related taxa are present in the samples, some reads will not map to E. coli as well as others. This is why we designed HVRLocator to systematically download 10,000 reads per sample to assess the mapping of these reads against our model E. coli on average. We see no indication of the presence of distantly related organisms in a sample affecting our ability to infer the target region sequenced, as confirmed by our assessment of the region sequenced across the methods stated in the literature for a subset of our samples.<br>We acknowledge that the ability to change the mapping sequence is useful, and would greatly expand the functionality of HVRLocator, and have built the tool foreseeing this expansion in the future, and we know acknowledge this in Line 389-393, although the accuracy against other mapping sequences is beyond the scope of this paper. Indeed, we are currently testing it for fungal studies targeting the ITS region, and hope to release this new version in the coming years, after rigorous testing.<br><br>Lines 389-393: “Although the present work focuses on bacteria, we acknowledge that other domains of life, including Archaea and Eukaryotes, are relevant for future meta-analyses and synthesis studies. Consequently, future versions of the program aim to incorporate alternative model sequences representing Archaea and Eukaryota.”<br><br>B. For taxa with significant length variations or insertions/deletions (Indels), could forced alignment to the E. coli reference lead to misjudgment of start/end positions?<br><br>R/ We do not consider length variation to be a major issue, as HVRLocator aligns relatively short amplicons to the full-length E. coli 16S rRNA reference sequence. Although some taxa exhibit insertions/deletions (indels) or length variation, the 16S rRNA gene is highly conserved across Bacteria, ensuring sufficient sequence similarity for reliable alignment to the appropriate reference region.<br>While 16S rRNA sequences vary in length across taxa, our validation showed 99% agreement between predicted hypervariable regions and those reported in the original publications (metadata or NCBI records). Together, the conserved structure of the 16S rRNA gene and the strong empirical validation support that the current alignment strategy provides reliable start and end position estimates for hypervariable regions and adequately addresses the objectives of this study.<br><br>C. Have the authors evaluated the impact on accuracy if a more universal reference database (such as representative sequences from SILVA or Greengenes) were used?<br><br>R/ We did not evaluate the impact of using a more universal reference database, as the current approach yielded accurate, consistent, and importantly, efficient results for the purposes of this study. However, we agree that exploring the use of representative sequences from broader reference databases such as SILVA or Greengenes could be valuable, and we will consider this for future versions of the tool.<br><br>2. Rationality of the primer detection model (Random Forest based on Quality Scores)

2.1 The authors developed a Random Forest model to predict primer presence by analyzing the quality score distribution of the first 1,000 reads. Primer detection is typically based on the sequence itself rather than quality scores. Can the authors explain why quality scores were chosen as features?

R/ We appreciate the reviewer's point and welcome the opportunity to clarify our rationale. Illumina sequencing is known to generate distinct quality score patterns in the first few cycles when base diversity is low, as occurs when untrimmed primers are present at the start of all reads (Mitra et al., 2015). Thus, we chose to use quality scores as features because they serve as a practical proxy for detecting primer presence. This approach allows for accurate, sequence-agnostic primer detection, especially when the actual primer sequences used are unknown or highly variable across datasets, which is the case in synthesis work, and a key reason for our development of HVRLocator. We have added text in lines 139-145 in the manuscript indicating our rationale:

Lines 139-145: "RF performs well under moderate class imbalances when class-aware evaluation and sampling is applied [25,26]. Additionally, distinct quality score patterns are known to occur in the first few cycles of Illumina sequencing when base diversity is low [27] as is in the case of untrimmed primers at the start of reads. Quality score patterns have also been used to detect sequencing bias and artifacts by tools such as DADA2 [28] and Mapinsights [29]. Thus, this metric can serve as a proxy for detecting primer presence."

Mitra A. et al. (2015). "Strategies for Achieving High Sequencing Accuracy for Low Diversity Samples…"

2.2 Sequencing quality scores are influenced by technical factors such as sequencer status, reagent batches, and run cycles, which have no direct biological correlation with the presence of primers. Is there a risk that this model is "overfitting" specific sequencing platforms or datasets?

R/ This is a valid concern, and we took several steps to mitigate the risk of platform-specific overfitting. First, our training dataset included thousands of SRA runs generated from a range of sequencing platforms and protocols. Second, during validation, we tested the model across multiple independent datasets, including those with differing primer sets and sequencing technologies, and achieved consistently high performance (precision and recall >99%) regardless of platform. Third, we ensured that the features used generalize well across datasets by using stratified sampling and fixed model parameters. Similar uses of quality score patterns to model sequencing bias and detect artifacts have been validated in tools like DADA2 (Callahan et al., 2016) and Mapinsights (Das et al., 2023), supporting the robustness of such approaches. Our observed performance across diverse conditions suggests the model is not overfitting. We have now added text in the manuscript (lines 139-145) highlighting the use of quality score patterns by publicly available tools:

Lines 139-145: "RF performs well under moderate class imbalances when class-aware evaluation and sampling is applied [25,26]. Additionally, distinct quality score patterns are known to occur in the first few cycles of Illumina sequencing when base diversity is low [27] as is in the case of untrimmed primers at the start of reads. Quality score patterns have also been used to detect sequencing bias and artifacts by tools such as DADA2 [28] and Mapinsights [29]. Thus, this metric can serve as a proxy for detecting primer presence."

Das S. et al. (2023). "Mapinsights: deep exploration of quality issues and error profiles in high-throughput sequence data."

Callahan B. et al. (2016). "DADA2: High resolution sample inference from Illumina amplicon data"

Since the reads are already downloaded, why not directly use degenerate primer sequence matching (e.g., using Cutadapt or SeqKit logic) to determine primer presence? This seems to be a more direct and accurate method.

R/ Degenerate sequence matching (e.g., via Cutadapt or SeqKit) requires prior knowledge of all potential primer sequences used across datasets. This poses a challenge for large-scale curation of public datasets, which often lack standardized or complete primer metadata, especially when INSDC databases often lack a link between the publication and the data for a substantial portion of the datasets archived. Our method provides a generalizable and lightweight solution that does not rely on prior knowledge of primer sequences (mentioned in lines 358-364 of the manuscript). By bypassing primer-specific matching, we reduce computational cost and avoid the risk of missing primer variants due to sequence mismatches or degeneracy. We view our workflow as a complementary tool, particularly useful in high-throughput or poorly annotated datasets.

Line 358-364: “In contrast, HVRLocator does not rely on simulations or a priori primer information, and is designed for its application to existing datasets. HVRLocator operates directly on large, INSDC-archived metabarcoding datasets to identify the start and end positions of sequenced 16S rRNA amplicons, determine their corresponding hypervariable regions, and detect the presence primer sequences, generating the technical metadata that is needed for bioinformatics processing of the raw sequences.”

3. Verification of accuracy claims
In the validation section, the authors claim to achieve 100% accuracy on certain datasets. In bioinformatics tool development, a claim of 100% accuracy is often a red flag. Have the authors manually checked those samples marked as "correct" by the model that might suffer from edge effects or borderline cases?

R/ We fully agree that claims of 100% accuracy require scrutiny. This value refers to specific controlled datasets where the sequenced region and primer pairs were known from curated sources (e.g., MiCoDa). Regardless, we manually reviewed edge cases and anomalies flagged by our tool such as sequences with unusual read lengths or misannotated primer regions and confirmed that HVRLocator correctly identified true mismatches with the reported metadata. In several such cases, we found that the discrepancies arose from metadata errors rather than model misclassification. These actions are stated in lines 263-283 of the manuscript:

Lines 263-283: “To check the reliability of HVRLocator relative to manual extraction of metadata from the literature, we manually extracted data related to the primers used and the 16S rRNA HV region targets from all samples in dataset c. For a total of 18,426 samples, 16,771 samples were processed successfully without warnings; common issues included missing FASTQ files, low reads, alignment failures, and NCBI portal-related issues. Of the 16,771 samples processed, 1,712 (10%) did not produce results consistent with the literature (e.g. mismatches between the start region alignment and the reported primer, or incorrect HV region alignment compared with the reported HV region), underscoring the value of obtaining metadata from the sequence data directly, rather than from the literature. Finally, for the diverse data set that used both different 16S rRNA regions and sequencing setups (Figure 2d), HVRLocator accurately and rapidly assigned the alignment positions.
Importantly, validation highlighted HVRLocator tool’s ability to identify problematic sequences. For example, we observed 932 samples with abnormally long average sequence lengths (i.e., >600 bp) that exceeded the expected output lengths with Illumina platforms. Upon manually reviewing these sequences, we found that either the sequencing platform was incorrectly annotated in the metadata (NCBI or the associated publication), or the sequences did not correspond to the 16S rRNA gene but rather to the Internal Transcribed Spacer (ITS) region or the nifH gene. This highlights the use of HVRLocator as a curation tool for large datasets, where human errors in annotation can significantly impact downstream analysis.”

4. Dataset imbalance in the Random Forest model
For the Random Forest model, the authors used 882 samples with primers and 8,940 samples without primers for training. Such an extremely imbalanced dataset, even with stratified sampling, may cause the model to be biased towards the majority class.

R/ We thank the reviewer for this important point. We evaluated performance using class-specific metrics achieving a precision of 100% and recall of 99.55% for the “primer-present” class on the held-out test set. These figures demonstrate that the

Powered by Editorial Manager® and ProduXion Manager® from Aries Systems Corporation

model retained strong sensitivity and specificity for the minority class despite the imbalance. Random Forests are known to perform robustly under moderate imbalance when class-aware evaluation and sampling are applied (Chen et al., 2004; Saito & Rehmsmeier, 2015). We have added text to manuscript in lines 139-145 that highlights this aspect of RF models. We also acknowledge that future versions could incorporate additional balancing techniques (e.g., class weighting), but the current results give confidence that no skew bias is affecting performance.

To confirm the robustness of the RF model, we applied the same stratified sampling during the 80/20 train-test split and ensured that both classes were adequately represented in each fold (lines 152-158 of the manuscript). This “balanced” model (which was trained using 881 no-primer samples and 881 primer samples) yielded virtually identical performance compared to the original: primer recall remained at 0.994, primer precision at 1.000, and overall accuracy exceeded 99.7%. This confirms that the initial model was not biased by the class imbalance and that the features used provide strong separation between classes:

Lines 152-158: “The model was trained using scikit-learn’s RandomForestClassifier (v1.2.1) with 100 estimators and a fixed random seed (random_state=42), using an 80/20 stratified train-test split. The Random Forest model yielded a precision of 99.96% for the dataset without primers and 100% for the dataset with primers. Recall of the model using the “no-primer” and “primer” dataset was 100% and 99.55%, respectively. Full details on the model generation including the algorithm, versions and packages are available in the Supplementary Table S3.”

Lines 139-145: “RF performs well under moderate class imbalances when class-aware evaluation and sampling is applied [25,26]. Additionally, distinct quality score patterns are known to occur in the first few cycles of Illumina sequencing when base diversity is low [27] as is in the case of untrimmed primers at the start of reads. Quality score patterns have also been used to detect sequencing bias and artifacts by tools such as DADA2 [28] and Mapinsights [29]. Thus, this metric can serve as a proxy for detecting primer presence.”

Chen, C., Liaw, A., & Breiman, L. (2004). Using random forest to learn imbalanced data. University of California, Berkeley, 110(1-12), 24.

Saito, T., & Rehmsmeier, M. (2015). The precision-recall plot is more informative than the ROC plot when evaluating binary classifiers on imbalanced datasets. PloS one, 10(3), e0118432.

5. Comparison with existing tools
The manuscript mentions that no tool has been designed for this specific purpose, but this may overlook some existing general-purpose tools or scripts. Many pipelines (such as certain plugins in QIIME 2, USEARCH, etc.) possess functionalities to identify primers or evaluate amplicon regions. The authors should discuss how their tool compares to these existing workflows.

R/ Thank you for the comments. We have added the following text in lines 364-379:

Lines 364-379: “Additionally, some QIIME 2 plugins provide functionality that overlaps with certain steps of the HVRLocator workflow. For example, q2-cutadapt [38] uses cutadapt to remove adapter sequences, primers, and other unwanted sequences from high-throughput sequencing reads, thereby ensuring clean data for downstream analysis, but it requires prior knowledge of the exact primer or unwanted sequences used in the samples in order to accurately detect and remove them. Similarly, the quality-control filter-reads plugin [38] filters demultiplexed single- or paired-end sequences based on their alignment to a reference database using Bowtie 2 [39] and SAMtools [40] to remove contaminants (e.g., human DNA) or to retain only sequences that align to a specified reference, but also relies on alignment to external reference sequences and does not report hypervariable regions or primer information. USEARCH provides functions related to primer matching (e.g., search_oligodb, search_pcr, and search_pcr2) [41]. However, as with QIIME 2, users must supply the

primer sequences in advance to perform database matching. In contrast, HVRLocator eliminates the need for prior knowledge of the exact primer sequences by automatically inferring this information.”

Minor Comments
1. Confusion regarding processing speed metrics
The abstract mentions a processing speed of "0.147 samples per minute", but later the text mentions "6.5 samples per minute" and "one sample every 0.147 minutes". There is confusion regarding units and values in these three descriptions (is it samples per minute or minutes per sample?). Please unify and correct these data to ensure consistency.

R/ Thank you. The reviewer is correct. There is no confusion regarding the units or values; however, we agree that the message was not clear or fully consistent. The information was derived from Table S5.

The value reported in the phrase “...at an average rate of 6.5 samples per minute” corresponds to the mean of the values in the last column (“Samples processed per minute”).

The value reported in the statement “one sample every 0.147 minutes” corresponds to the mean of the column labeled “Time to process 1 sample.”

For greater clarity, we have revised the text as follows:

1.Abstract: “HVRLocator can process archived 16S rRNA sequences from NCBI SRA at 6.5 samples per minute.”
We modified the sentence as follows: “HVRLocator can process archived 16S rRNA sequences from NCBI SRA at an average rate of 6.5 samples per minute.”

3.Case Study: “HVRLocator processed approximately one sample every 0.147 minutes, using 8 GB of RAM and 4 CPU cores.”
We modified the sentence as follows: “HVRLocator processed samples at an average rate of 6.5 samples per minute, using 8 GB of RAM and 4 CPU cores.”

2. Usage of fastq-dump
The use of fastq-dump is mentioned. The SRA Toolkit's fastq-dump is relatively slow and has largely been superseded by fasterq-dump for efficiency. Why did the authors not use the more efficient fasterq-dump?

R/ We thank the reviewer for pointing this out. We agree that fasterq-dump is generally more efficient and represents the recommended approach for large-scale data retrieval. In the current implementation, however, we download only the first 10,000 reads from each sample for primer inference. Under these conditions, the overall runtime is dominated by the time required to establish connections to the server rather than by the data transfer itself, and the use of a multi-threaded tool such as fasterq-dump does not result in a substantial performance improvement. Nevertheless, we acknowledge the reviewer’s suggestion and will consider adopting fasterq-dump in future versions of the tool to improve efficiency.

3. Definition of "Standardized metadata"
The term "standardized metadata" is used frequently. Please explicitly define what constitutes "standard" metadata in the context of this tool within the text.

R/ Thank you for the observation. We have added the following text in lines 95-98:

Lines 95-98: “Standardized metadata is structured information that follows agreed-upon standards, ensuring consistency and comparability across studies and databases through defined fields, controlled vocabularies or ontologies, and standardized formats.”

4. Robustness and error handling

The results section mentions that some samples failed due to "NCBI portal-related issues". Does this imply the tool lacks breakpoint resumption or retry mechanisms?

R/ We apologize for the confusion. The tool includes a retry mechanism to handle temporary NCBI portal issues. The reported failures were due to persistent external server problems rather than a lack of retry mechanisms in the tool. We have added this clarification to the results section:

Lines 239-241: “Although HVRLocator incorporates an automatic retry mechanism to mitigate temporary NCBI portal interruptions, a small number of samples failed due to persistent external server-side issues beyond the control of the tool.”

Given that network fluctuations are common during large-scale downloads, how is the tool's robustness demonstrated?

R/ For any error in the download of a dataset using “fastq-dump”, we have implemented a retry function that tries to retrieve the data three times before moving to the next entry. This way we assure to bypass any temporary issues at the servers level.
Lastly, at the end of a whole run, the user can select the IDs of the failed entries and submit again to a new run. We opted to not automatically try the “failed” downloads again at the end and let the user decide what to do with those. From our experience, these runs are usually simply not available in the Sequence Read Archive (SRA) (Jurburg et al, 2020).

Jurburg, S.D., Konzack, M., Eisenhauer, N. and Heintz-Buschart, A. (2020) The archives are half-empty: an assessment of the availability of microbial community sequencing data. Communications Biology; 3 (474)

5. Output confidence intervals
The output file contains "TRUE/FALSE" and a probability score. For samples where the probability score is at a critical threshold (e.g., around 0.5), does the tool provide an "uncertain" tag, or does it force a classification? It is suggested to add an indicator for ambiguous ranges.

R/ The cutoff value used (0.5) represents a validated and balanced threshold that is not overly conservative, thereby avoiding an excessive number of false positives. However, we provide users with the underlying probability scores values, which can be manually adjusted directly within the results table to apply custom filtering according to their preferences. This allows users to disregard the binary TRUE/FALSE classification and instead filter results based solely on the probability score.
HVRLocator uses the 0.5 threshold by default because it was validated using the Random Forest model. Any alternative threshold selected by the user is applied at their own judgment and responsibility.

We add the following text in the main manuscript in order to clarify this:

Line 196-200:“Additionally, HVRLocator provides the underlying probability scores for primer presence, allowing users to manually adjust filtering based on their own criteria instead of relying solely on the default TRUE/FALSE classification. The default 0.5 threshold was validated using the Random Forest model, and any alternative threshold chosen by the user is applied at their own discretion.”

--
6. Please also take a moment to check our website at https://www.editorialmanager.com/giga/l.asp?i=238196&l=G6VFB430 for any additional comments that were saved as attachments. Please note that as GigaScience has a policy of open peer review, you will be able to see the names of the reviewers.

R/ Done.

In compliance with data protection regulations, you may request that we remove your personal registration details at any time. (Use the following URL:

	https://www.editorialmanager.com/giga/login.asp?a=r). Please contact the publication office if you have any questions.
**Additional Information:**	
**Question**	**Response**
Are you submitting this manuscript to a special series or article collection?	No
**Experimental design and statistics**<br><br>Full details of the experimental design and statistical methods used should be given in the Methods section, as detailed in our Minimum Standards Reporting Checklist. Information essential to interpreting the data presented should be made available in the figure legends.<br><br>Have you included all the information requested in your manuscript?	Yes
**Resources**<br><br>A description of all resources used, including antibodies, cell lines, animals and software tools, with enough information to allow them to be uniquely identified, should be included in the Methods section. Authors are strongly encouraged to cite Research Resource Identifiers (RRIDs) for antibodies, model organisms and tools, where possible.<br><br>Have you included the information requested as detailed in our Minimum Standards Reporting Checklist?	Yes
**Availability of data and materials**<br><br>All datasets and code on which the conclusions of the paper rely must be either included in your submission or deposited in publicly available repositories (where available and ethically appropriate), referencing such data using a unique identifier in the references and in the “Availability of Data and Materials”	Yes

section of your manuscript.<br><br>Have you have met the above requirement as detailed in our Minimum Standards Reporting Checklist?	
GigaScience has policies and guidelines in place for the use of generative AI-writing tools such as ChatGPT. If you have used such writing tools to assist with writing the manuscript this must be declared and cited in the text. Authors should not list AI-writing tools and other AI-assisted technologies as an author or co-author and should acknowledge that they are fully responsible for text generated or refined by AI-writing tools.<p><br><br>A summary of use (particularly in the introduction or among methods) needs to be included at the end of the paper, and the outputs should also be included as a supplementary file hosted in GigaDB or other open repositories. Please read our guidelines for more information. <p><br><br>By submitting to GigaScience, you are aware of the journal's AI-writing tools policy, and if you have declared use of such tools below, you have acknowledged this where appropriate in your manuscript and have made a summary of use and outputs available. <p><br><b>AI-assisted writing tools have been used in the preparation of this manuscript?	No

Manuscript

Click here to access/download;Manuscript;GIGA-D-25-00344_HVRLocator_Manuscript_202602.pdf

HVRLocator: A Computationally Efficient Tool for Identifying Hypervariable Regions in Large 16S rRNA Datasets

Clara Arboleda-Baena[1‡], Felipe Borim Correa[2‡], Joao Pedro Saraiva[2], Santiago Castillo-Rivadeneira[1], Jonas Coelho Kasmanas[2], Antonis Chatzinotas[1,2,3], Stephanie D. Jurburg[1,2*]

[1] German Centre for Integrative Biodiversity Research (iDiv) Halle-Jena-Leipzig, 04103 Leipzig, Germany

[2] Department of Applied Microbial Ecology, Helmholtz Centre for Environmental Research - UFZ, 04318 Leipzig, Germany

[3] Institute of Biology, Leipzig University, 04103 Leipzig, Germany

‡*CAB and FBC contributed equally to this work*

*Corresponding authors: claraarboledab@gmail.com and s.d.jurburg@gmail.com

Abstract

Background: Metabarcoding of the 16S rRNA gene is widely used to assess microbial diversity due to its cost-effectiveness and efficiency. However, publicly available 16S rRNA metabarcoding datasets often lack standardized metadata, particularly information on the sequenced hypervariable regions or primers used, which are critical to their accurate reuse. To address this, we present HVRLocator, a computational tool that (1) identifies the start and end positions of 16S rRNA amplicons, (2) determines their corresponding hypervariable regions, and (3) detects the presence of primer sequences. This tool was validated on four datasets comprising 41,513 samples generated with different primers and sequencing platforms.

Results: HVRLocator can process archived 16S rRNA sequences from NCBI SRA at an average rate of 6.5 samples per minute. Validation showed it reliably detects amplicon start and end positions across datasets sequenced with different primers and platforms, achieving 100% accuracy within single-platform studies and correctly revealing length heterogeneity across platforms. It also flagged misannotated metadata and problematic

sequences, underscoring its value as a sequence data curation tool. Finally, HVRLocator can select comparable sequences to build large 16S rRNA amplicon databases spanning the same hypervariable region, facilitating cross-study comparisons.
Conclusion: HVRLocator overcomes unreliable metadata by accurately identifying 16S rRNA amplicon start and end positions, determining hypervariable regions, and detecting primer sequences, enabling accurate curation and large-scale processing of 16S rRNA data for reliable and reproducible microbial studies, syntheses, and meta-analyses.

Keywords: Big data, 16S rRNA gene, metabarcoding, high throughput sequencing, metadata, microbial ecology.

Background

While the existence of bacteria has been known for over three centuries, the ability to study all individuals in a bacterial community is relatively novel. By extracting and sequencing nucleic acids from hosts or environmental samples, it is now possible to characterize the taxonomic diversity of a bacterial community without the need to cultivate its members. Metabarcoding, which focuses sequencing efforts on a segment of a universal marker gene, or amplicon (typically the 16S rRNA gene for prokaryotes), has emerged as a dominant technique due to its technical ease and low cost. To date, 16S rRNA gene metabarcoding has uncovered the extreme diversity and ubiquity of microbes [1], while revealing avenues for improving human health, agricultural productivity, and sustainability [2]. At the same time, metabarcoding datasets archived in public repositories have grown exponentially [3]. These data are uniform in format, are routinely archived with technical and experimental metadata, and are a rich and growing resource for synthetic and large-scale research. However, metabarcoding data are archived in their raw format, and the metadata needed for bioinformatics processing is often unavailable or not curated, creating barriers to data reuse [3,4].

Technical metadata are central to sequence data harmonization and reuse as they provide context for the data [5] and directly inform bioinformatics processing. Technical choices preceding sequencing, most notably the DNA extraction kit [6], sequencing

platform [7], and target amplicon [8,9] have been shown to affect microbial diversity assessments [10]; however the integration of these data *in light* of Big Data processing and synthesis has received less attention. For example, Abdill and colleagues [11] restricted their synthesis to sequences obtained from Illumina technologies to use a unified processing pipeline, but did not enforce consistent amplicon sequence lengths, even though detected bacterial diversity increases linearly with amplicon length [12].

The 16S rRNA gene contains both highly conserved regions that are essential for primer design, and hypervariable regions that allow for the phylogenetic identification of microorganisms [13]. Full-length 16S rRNA gene sequences (~1500 bp) comprise nine hypervariable regions interspersed with nine highly conserved regions [14,15]. Identifying which 16S rRNA gene segment was targeted for amplification is crucial to fully leverage sequence length and coverage, which ultimately determines the efficiency and accuracy of downstream processing and taxonomic classification pipelines. In the case of paired-end reads (e.g., Illumina technologies), the length of the target region further informs the minimum read length needed to achieve a successful merger of the pair [16].

At the same time, the choice of the sequenced region can significantly affect the relative abundances of detected organisms. For example, Wasimuddin et al. [8] found that compared to three other primer sets targeting different regions, the primer pair targeting the V4 hypervariable region of the 16S rRNA gene produced the highest estimates of Amplicon Sequence Variants (ASVs) richness and diversity across various sample types (including soil, maize roots, cattle rumen, and cattle and human feces). However, different primers targeting the same variable region can still generate different numbers of ASVs [17].

Crucially, as novel sequencing technologies and platforms have emerged, the length of the target amplicons has also varied extensively, ranging from 150 base pair segments (e.g., Illumina HiSeq single-end sequencing) to whole genes (e.g., nanopore sequencing), resulting in massive heterogeneity in the length and location of the sequenced regions. Variation in amplicon length complicates the reuse of 16S rRNA metabarcoding data,

since longer sequences tend to detect greater bacterial diversity and taxonomic resolution [12].

Considering technical information about the sequenced region during downstream bioinformatics processing is essential to metabarcoding data reuse, but this information is often missing from the metadata, vague, or incorrect [18]. Standardized metadata is structured information that follows agreed-upon standards, ensuring consistency and comparability across studies and databases through defined fields, controlled vocabularies or ontologies, and standardized formats. The lack of standardized metadata significantly slows down the compilation of large datasets, making it difficult to reprocess metabarcoding sequences collectively. Here, we present HVRLocator, a computational tool designed to efficiently identify hypervariable regions of the 16S rRNA gene sequenced for a set of metabarcoding samples. By optimizing computational resources, our approach enables rapid and accurate screening of large datasets, facilitating more comprehensive and scalable microbial diversity analyses.

Materials and methods

Design

HVRLocator identifies which segment of the 16S rRNA gene was sequenced for a given set of metabarcoding samples. The full pipeline uses Python programming language, and users can access it either via a singularity container or by installing it locally on their computer. For further details on installation and usage please see: https://github.com/fbcorrea/hvrlocator/.

As input, HVRLocator accepts text file (.txt) lists of accession numbers compliant with International Nucleotide Sequence Collaboration (INSDC) databases, including the European Nucleotide Archive (ENA) at the European Bioinformatics Institute, the Sequence Read Archive (SRA) at the National Center for Biotechnology Information, and the DNA Databank of Japan (DDBJ) Sequence Read Archives at the National Institute of Genetics [19], bypassing the need to download the data a priori. HVRLocator also accepts resolved amplicons (e.g., Amplicon Sequence Variants (ASV)) or raw sequencing data in .FASTA or .FASTQ file formats of the 16S rRNA gene.

For INSDC data, the tool employs a multi-step process. First, it retrieves 1000 reads using fastq-dump from the SRA Toolkit [20] and performs quality control and trimming with fastp [21]. Then, for each sample, the HVRLocator identifies the data type (single- or paired-end DNA). For paired-end reads, it merges the processed reads using VSEARCH [22], while for single-end reads, it directly converts the trimmed FASTQ to FASTA format. The tool then aligns the processed sequences to a reference 16S rRNA gene sequence from an *Escherichia coli* reference genome (J01859.1) using MAFFT [23]. HVRLocator subsequently analyzes the aligned sequences to determine hypervariable regions by identifying the median start and end alignment positions relative to the *E. coli* 16S rRNA gene reference sequence, using established coverage thresholds (default = 0.6) for the conserved and hypervariable regions of the 16S rRNA gene [13,24]. To note, the thresholds can be adjusted by adding the "-t" flag. The output is a tab-separated values (TSV) file containing alignment start and end positions, as well as the boundaries (median and average start and end positions, and minimum start and maximum end positions) of the identified hypervariable regions. HVRLocator is currently limited to bacterial 16S rRNA sequences, as it aligns them to the E. coli 16S rRNA reference.

Finally, a Random Forest (RF) model was designed to predict the presence of a primer in a given SRA sequencing dataset by analyzing the quality score distribution of the initial subset of reads. RF performs well under moderate class imbalances when class-aware evaluation and sampling is applied [25,26]. Additionally, distinct quality score patterns are known to occur in the first few cycles of Illumina sequencing when base diversity is low [27] as is in the case of untrimmed primers at the start of reads. Quality score patterns have also been used to detect sequencing bias and artifacts by tools such as DADA2 [28] and Mapinsights [29]. Thus, this metric can serve as a proxy for detecting primer presence. To this end, we selected a curated collection of SRA samples with (882 samples) and without primers (8940 samples, **Supplementary Tables S1 and S2**). For each sample, the first 1,000 reads from each sample were extracted using fastq-dump (NCBI SRA Toolkit, 3.2.1) and two quality score segments from positions 1-5 and 6-10 were calculated. Eight statistical features were obtained: count, mean, median, standard deviation, minimum, maximum, an estimate of skewness (approximated by the 25th percentile), and kurtosis (approximated by the 75th percentile), resulting in 16 features

per sample. The model was trained using scikit-learn's RandomForestClassifier (v1.2.1) with 100 estimators and a fixed random seed (random_state=42), using an 80/20 stratified train-test split. The Random Forest model yielded a precision of 99.96% for the dataset without primers and 100% for the dataset with primers. Recall of the model using the "no-primer" and "primer" dataset was 100% and 99.55%, respectively. Full details on the model generation including the algorithm, versions and packages are available in the **Supplementary Table S3.**

HVRLocator is available as a singularity container located at https://cloud.sylabs.io/library/jsaraiva/repo/hvrlocator, and can be executed on High Performance Computing (HPC) clusters or cloud computing platforms. Further, Singularity enables the seamless execution of containers without requiring root privileges, maintaining security and reproducibility. HVRLocator will be actively maintained with updates for compatibility and user feedback. Issues or feature requests can be sent to the corresponding author. The HVRLocator output is a text file (.txt) containing the following columns:

1.Sample_ID: Identifier of the processed sample (Run Accession number).
2.Primer Presence: Presence or absence of a primer (TRUE/FALSE)
3.Score Primer Presence: The associated probability value ranging from 0 to 1.
4.Min/Max Alignment Start/End: Minimum (0) and maximum (1540) possible alignment positions along the 16S rRNA gene.
5.Average Alignment Start/End: The mean position where reads align to the 16S rRNA gene. The *start* indicates the average starting position, and the *end* indicates the average ending position across all reads in the sample.
6.Median Alignment Start/End: The median position where reads align to the 16S rRNA gene. The start indicates the median starting position, and the end indicates the median ending position across all reads in the sample.
7.Predicted HV region Start/End: Predicted hypervariable (HV) region based on the median alignment start and end positions across all reads, inferred from literature on conserved and hypervariable regions of the 16S rRNA gene (Brosius et al., 1978; Yang et al., 2016).

8.Coverage based HV region Start/End: Predicted hypervariable region based on coverage at the start and end positions across all reads.

9.Coverage HV region Start/End: Coverage values (0-1) for the "*Coverage based HV region*" start or end position across all reads. 0 = no reads cover that position; 1 = all reads cover that position

10.Warnings: Alerts about low coverage regions. See possible errors and troubleshooting.

11-19. Cov_V1 to Cov_V9: Coverage values (0-1) for each HV region.

Importantly, validation showed that the median alignment position is a more reliable indicator of the sequenced region than the average alignment position, as low-quality sequences within a sample can skew the mean and lead to an incorrect identification of the hypervariable region. For this reason, we report both metrics, but recommend prioritizing the median when deciding whether to retain or discard sequences during downstream processing. Additionally, HVRLocator provides the underlying probability scores for primer presence, allowing users to manually adjust filtering based on their own criteria instead of relying solely on the default TRUE/FALSE classification. The default 0.5 threshold was validated using the Random Forest model, and any alternative threshold chosen by the user is applied at their own discretion.

Finally, in addition to identifying the hypervariable region based on the average and median start and end positions of the amplicons within each sample, HVRLocator also reports coverage across all nine hypervariable regions for every individual sample. This allows users to assess the amplicon length, determine its exact start and end positions, and evaluate the coverage of specific hypervariable region(s) within each sample.

Validation

HVRLocator's processing stability was calculated by measuring the sample time for 1, 10, 100, 1000 and 10000 samples using the same cluster resources: 8 GB of RAM and 4 CPU cores to emulate the standard capabilities of a personal computer.

HVRLocator was validated by analyzing four datasets which contained samples sequenced using a) same primer and same sequencing platform, b) different primers and

the same platform, c) the same primer and different sequencing platforms, and d) different primers and sequencing platforms. Dataset *a* included 17,537 samples from the Earth Microbiome Project (https://earthmicrobiome.org), in which all samples were sequenced on the Illumina Miseq platform using the 515F–806R primer set targeting the V4 hypervariable region of the 16S rRNA gene [30]. Dataset *b* included 242 samples from two studies that compared different primers but were sequenced on the same platform [8,31]. Dataset *c* included 18,426 samples from the MiCoDa database Version 1, which were compiled from available literature and data (https://micoda.idiv.de/) [32]**,** to select samples that were sequenced in the same 515-806 region of the 16S SSU rRNA (Small Subunit Ribosomal RNA) with various sequencing platforms. The primer information for each sample was obtained from the metadata archive in NCBI. For dataset *d,* we selected 5,308 samples compiled during the Datathon project in Latin America [33]**.** These samples employed various primer sets targeting different regions of the 16S rRNA gene and were sequenced on different platforms, and represent a realistic set of samples that might be encountered during data compilation efforts. The run accession numbers used for all datasets are listed in **Supplementary Table S4, 1-4**.

All graphics were carried out in R with RStudio interface [34], and all pipelines are available at: https://github.com/ClarArboledaBaena/HVRLocator-Figures.

Results

As the number of samples increased, the running time per sample and computational resource usage remained stable at an average rate of 6.5 samples per minute (**Figure 1 and Supplementary Table S5**), highlighting the tool’s processing stability and scalability for the analysis of large datasets. Failures were primarily due to samples with fewer than 500 reads (72%), alignment errors (14%), missing FASTQ files (13%), and NCBI portal-related issues (1%). (See *Possible Errors and Troubleshooting* at https://github.com/fbcorrea/hvrlocator). Although HVRLocator incorporates an automatic retry mechanism to mitigate temporary NCBI portal interruptions, a small number of samples failed due to persistent external server-side issues beyond the control of the tool. By analyzing the alignment positions across different 16S rRNA hypervariable regions and sequencing setups (**Figure 2**), we found that HVRLocator accurately predicted the

alignment positions compared to what is expected from the literature associated with each database. For example, sequences in dataset *a,* consistently aligned with the V4 hypervariable region of the 16S rRNA gene and had a median sequence start of 532 bp, as expected from the standardized primer set (515F-806R) used in the Earth Microbiome Project. Sequence lengths were highly homogeneous, also likely due to the use of the same primer set and sequencing machinery (**Figure 2a**). For a total of 17,537 samples, 16,059 samples were processed successfully without warnings; common issues included missing FASTQ files, low reads, alignment failures, and NCBI portal-related issues. Out of 16,059 samples processed, 9 did not yield the expected results based on the literature. Dataset *b* [8,31], which employed different primers but the same platform, confirmed that HVRLocator correctly matched the sequences to the corresponding, expected primer regions used during sample sequencing with 100% accuracy (**Figure 2b**). For dataset *c* HVRLocator also accurately indicated more heterogeneous alignment start positions and sequence lengths, consistent with our expectation and the compilation of the MiCoDa database from the literature (**Figure 2c)**. Most sequences covered the V4 region (89%), as indicated by both the median alignment start and the coverage-based HV region start (**Figure 2c and Figure 3**, respectively). As expected, the median alignment end varied across projects and sequencing platforms.

To check the reliability of HVRLocator relative to manual extraction of metadata from the literature, we manually extracted data related to the primers used and the 16S rRNA HV region targets from all samples in dataset *c.* For a total of 18,426 samples, 16,771 samples were processed successfully without warnings; common issues included missing FASTQ files, low reads, alignment failures, and NCBI portal-related issues. Of the 16,771 samples processed, 1,712 (10%) did not produce results consistent with the literature (e.g. mismatches between the start region alignment and the reported primer, or incorrect HV region alignment compared with the reported HV region), underscoring the value of obtaining metadata from the sequence data directly, rather than from the literature. Finally, for the diverse data set that used both different 16S rRNA regions and sequencing setups (**Figure 2d**), HVRLocator accurately and rapidly assigned the alignment positions.

Importantly, validation highlighted HVRLocator tool's ability to identify problematic sequences. For example, we observed 932 samples with abnormally long average sequence lengths (i.e., >600 bp) that exceeded the expected output lengths with Illumina platforms. Upon manually reviewing these sequences, we found that either the sequencing platform was incorrectly annotated in the metadata (NCBI or the associated publication), or the sequences did not correspond to the 16S rRNA gene but rather to the Internal Transcribed Spacer (ITS) region or the nifH gene. This highlights the use of HVRLocator as a curation tool for large datasets, where human errors in annotation can significantly impact downstream analysis.

Case study: 45,882 metabarcoding samples for the compilation of a large 16S rRNA gene database.

We present an example of how to use the tool to select correct and comparable sequences to construct a large bacterial database based on metabarcoding sequences targeting the V4 hypervariable region of the 16S rRNA gene. We included samples sequenced from start position 515 bp of the 16S rRNA gene, which is the same starting position as that used by the Earth Microbiome Project primers (515F–806R) [1]. Our input dataset included 45,882 samples spanning a wide variety of matrices (e.g., soil, host-associated, and water), that were sequenced with different primer sets and sequencing platforms. These data were collected through an extensive literature search prioritizing meta-analyses, large amplicon research consortia, and Datathon activities [33] (**Supplementary Table S6**). Using SRA's sequence-associated metadata, we selected only metabarcoding-derived sequences (i.e., excluding WGA, WGS, Tn-Seq, miRNA-Seq, POOLCLONE, RNA-Seq, etc).

HVRLocator processed samples at an average rate of 6.5 samples per minute, using 8 GB of RAM and 4 CPU cores.. A total of 42,166 samples were processed successfully, while 3,716 samples failed to be processed and generated warnings, mainly due to samples with fewer than 500 reads (66%), missing FASTQ files (21%), alignment errors (13%), and other issues related to the NCBI portal (1%). (See *Possible Errors and Troubleshooting* at https://github.com/fbcorrea/hvrlocator). HVRLocator identified a diverse range of sequences with varying median hypervariable region start and end

positions, coverage, and lengths **(Figure 4a)**. The output indicated that 1,532 samples had a true presence of primers, while 40,634 did not.

The detailed output for the average and median alignment columns, as well as the coverage-based start and end values, is provided in **Supplementary Table S7a–d,f.** Of the successfully processed samples, 85.9% (36,217) began in the V4 region of the 16S rRNA gene (**Figure 4b**), and the next most common starting regions were V3 (9.7%), V2 (2.3%), and V1 (1.5%). The predicted end of the hypervariable region for the majority of samples corresponded to the V4 region (85.3%), followed by V6 (6.5%), V8 (3.4%), V5 (1.6%), and V7 (1.4%). (**Supplementary Table S7e**). We retained the 36,217 samples that had a median starting point in the V4 region.

For all sequences, we cross-checked the reported primer information either from the metadata of the research articles or NCBI records against the region predicted by HVRLocator (**Figure 4d**). This allowed us to quantify the number of samples where the actual sequenced region was incorrectly assigned, despite being labeled as targeting the V4 hypervariable region in the metadata. Among the selected samples, 382 (1%) had incorrect primer annotations, either in the NCBI metadata or in the associated publications.These findings highlight that even when metadata is available, it may be inaccurate or misleading and underscores the importance of HVRLocator, which accurately and efficiently identify the sequenced region.

Discussion

INSDC databases currently host over 32 million next-generation sequencing samples [35], and represent a growing resource for large-scale analyses to address global questions through the synthesis and reuse of sequence data. However, efforts of sequence data archiving are undermined by the lack of available metadata [20], especially, as these metadata are crucial to data processing. High quality data are relatively sparse [3], which makes the process of data identification intensive, inefficient, and error-prone. To facilitate the reuse of bacterial metabarcoding data, we developed HVRLocator, a publicly available tool which efficiently identifies the exact region sequenced by a set of 16S rRNA sequences, and can therefore greatly accelerate the

identification of candidate datasets for reanalysis. The extensive validation of HVRLocator also highlights its potential for application towards data reuse.

Given the ubiquity of bacteria and their relevance to their environments, a wide range of disciplines employ 16S rRNA gene metabarcoding sequencing, and contribute data to INSDC archives in the process [35]. Indeed, according to available ENA metadata, metabarcoding datasets still dwarf metagenomic datasets by a factor of ten. Due to the lack of curation of INSDC metadata, information derived from peer-reviewed literature has been proposed as a central source of technical metadata that can enrich existing datasets [36], but the diversity of disciplines which employ metabarcoding also results in different degrees of resolution in the technical metadata provided for the sequence data. These metadata may lack the resolution necessary for an improved bioinformatics process or even introduce errors. Here, HVRLocator serves to bypass the need to return to the original literature to obtain the necessary processing metadata and the higher resolution information, such as the exact start and end sequence positions instead of the general region sequenced, and to correct potential errors that might be present in the literature-derived metadata.

To our knowledge, no tool has been designed with the specific purpose of automated metadata extraction from archived metabarcoding datasets to facilitate bioinformatics processing. Based on complete or near-complete 16S rRNA gene sequences and for a given set of primers, HyperEx (HyperVariable Region Extractor) [37] evaluates the precision and accuracy of different primer pairs in retrieving microbial diversity, and was developed as a tool for primer selection. Similarly, Qscore [9] evaluates the performance of 16S rRNA amplicons, assessing metrics including amplification rate, multitier taxonomic annotation, sequence type, and length. In contrast, HVRLocator does not rely on simulations or *a priori* primer information, and is designed for its application to existing datasets. HVRLocator operates directly on large, INSDC-archived metabarcoding datasets to identify the start and end positions of sequenced 16S rRNA amplicons, determine their corresponding hypervariable regions, and detect the presence primer sequences, generating the technical metadata that is needed for bioinformatics processing of the raw sequences. Additionally, some QIIME 2 plugins provide

functionality that overlaps with certain steps of the HVRLocator workflow. For example, q2-cutadapt [38] uses cutadapt to remove adapter sequences, primers, and other unwanted sequences from high-throughput sequencing reads, thereby ensuring clean data for downstream analysis, but it requires prior knowledge of the exact primer or unwanted sequences used in the samples in order to accurately detect and remove them. Similarly, the quality-control filter-reads plugin [38] filters demultiplexed single- or paired-end sequences based on their alignment to a reference database using Bowtie 2 [39] and SAMtools [40] to remove contaminants (e.g., human DNA) or to retain only sequences that align to a specified reference, but also relies on alignment to external reference sequences and does not report hypervariable regions or primer information. USEARCH provides functions related to primer matching (e.g., search_oligodb, search_pcr, and search_pcr2) [41]. However, as with QIIME 2, users must supply the primer sequences in advance to perform database matching. In contrast, HVRLocator eliminates the need for prior knowledge of the exact primer sequences by automatically inferring this information.

Technical metadata is crucial, as species identification depends heavily on the targeted region and its length [12]. In the case of 16S rRNA gene metabarcoding, information about the gene region sequenced is essential for the bioinformatics processing of the sequence data, and for the statistical analyses (i.e., as a random effect in a hierarchical model) and downstream data interpretation. From an ecological perspective, the ability to consistently target the same genetic region across different studies brings us closer to achieving a macroecological understanding of microbial communities [8,12,42]. In this vein, HVRLocator can support and accelerate the bioinformatics processing of 16S rRNA metabarcoding data, enhancing comparability and improving short-read training sets for future predictive microbiome studies. Although the present work focuses on bacteria, we acknowledge that other domains of life, including Archaea and Eukaryotes, are relevant for future meta-analyses and synthesis studies. Consequently, future versions of the program aim to incorporate alternative model sequences representing Archaea and Eukaryota.

In the future, HVRLocator may support decision-making in the creation of large databases, improving the robustness and resolution of microbiome studies [13,43,44]. As long-read sequencing technologies gain relevance, HVRLocator could serve as a foundation for developing procedures to integrate multiple sequences with different coverage, ultimately enhancing our ability to capture microbial diversity more comprehensively.

Availability of supporting source code and requirements

Project name: HVRLocator

Project home page: https://github.com/fbcorrea/HVRLocator

Operating system(s): Linux OS

Programming language: Python 3.9

Other requirements: Singularity container platform https://cloud.sylabs.io/library/jsaraiva/repo/hvrlocator

License: CC0 1.0 Universal

RRID: SCR_027407

bio.tools ID: biotools:hvrlocator - https://bio.tools/hvrlocator

Additional files

Supplementary Table S1: Run accession list with primers used for training and testing the Random Forest (RF) model.

Supplementary Table S2: Run accession list without primers used for training and testing the Random Forest (RF) model.

Supplementary Table S3: Random Forest Model for Primer Presence Prediction.

Supplementary Table S4: Datasets (1-4) included in the validation process.

Supplementary Table S5: Number of samples per dataset and run time (in minutes) using 8 GB of RAM and 4 CPU cores.

Supplementary Table S6: Run accession list for case study

Supplementary Table S7: Case study output.

Supplementary Table S8: Number of samples in which primer assignment matched the metadata (Figure S1.b)

Supplementary Figure S1: Variation in gene coverage across sequences and number of samples in which primer assignment matched the metadata.

Data Availability

The public datasets used in this paper can be found in **Supplementary Table S1, S2, S4** and **S6**.

Abbreviations

ASV: Amplicon Sequence Variants; DDBJ: DNA Databank of Japan; DRA: Sequence Read Archives; EBI: European Bioinformatics Institute (EBI); ENA: European Nucleotide Agency; HPC: High Performance Computing; INSDC: International Nucleotide Sequence Database Collaboration; ITS: Internal Transcribed Spacer; NCBI: National Center for Biotechnology Information; NIG: National Institute of Genetics; SRA: Sequence Read Archive; SSU rRNA: Small Subunit Ribosomal RNA; TSV: Tab-separated values; UFZ: Helmholtz Center for Environmental Research.

Acknowledgments

The results were computed at the High-Performance Computing (HPC) Cluster EVE, a joint effort of both the Helmholtz Centre for Environmental Research - UFZ (http://www.ufz.de/) and the German Centre for Integrative Biodiversity Research (iDiv) Halle-Jena-Leipzig (http://www.idiv-biodiversity.de/). We would like to thank the administration and support staff of EVE who keep the system running and support us with our scientific computing needs: Toni Harzendorf, Mark Fliak and Conrad Ostertag from UFZ, and Christian Krause from iDiv. Also, we would like to thank Marten Winter at the Synthesis Centre for Biodiversity Sciences (sDiv) at the German Centre for Integrative Biodiversity Research (iDiv).

Author Contributions

Conceptualization: SJ, FBC, CAB; funding acquisition: SJ; methodology: SJ, FBC, CAB, JPS, SC; software: FBC, JPS, JCK; writing—original draft: SJ, FBC, CAB, JPS; writing—review and editing: all authors. All authors read and approved the final manuscript.

Funding

sIBTEDS project (Illuminating Blindspots Through Equitable Data Reuse practices in the Global South) from the German Centre for Integrative Biodiversity Research (iDiv).

Competing Interests

The authors declare that they have no competing interests

References

1. Thompson LR, Sanders JG, McDonald D, Amir A, Ladau J, Locey KJ, et al.. A communal catalogue reveals Earth's multiscale microbial diversity. *Nature*. 2017; doi: 10.1038/nature24621.

2. Jurburg SD, Eisenhauer N, Buscot F, Chatzinotas A, Chaudhari NM, Heintz-Buschart A, et al.. Potential of microbiome-based solutions for agrifood systems. *Nat Food*. 2022; doi: 10.1038/s43016-022-00576-x.

3. Jurburg SD, Konzack M, Eisenhauer N, Heintz-Buschart A. The archives are half-empty: an assessment of the availability of microbial community sequencing data. *Commun Biol*. 2020; doi: 10.1038/s42003-020-01204-9.

4. Hassenrück C, Poprick T, Helfer V, Molari M, Meyer R, Kostadinov I. FAIR enough? A perspective on the status of nucleotide sequence data and metadata on public archives. *bioRxiv*. Cold Spring Harbor Laboratory; 2021; doi: 10.1101/2021.09.23.461561.

5. Leigh DM, Vandergast AG, Hunter ME, Crandall ED, Funk WC, Garroway CJ, et al.. Best practices for genetic and genomic data archiving. *Nat Ecol Evol*. Springer Science and Business Media LLC; 2024; doi: 10.1038/s41559-024-02423-7.

6. Tremblay J, Singh K, Fern A, Kirton ES, He S, Woyke T, et al.. Primer and platform effects on 16S rRNA tag sequencing. *Front Microbiol*. Frontiers Media SA; 2015; doi: 10.3389/fmicb.2015.00771.

7. Whon TW, Chung W-H, Lim MY, Song E-J, Kim PS, Hyun D-W, et al.. The effects of sequencing platforms on phylogenetic resolution in 16 S rRNA gene profiling of human feces. *Sci Data*. Springer Science and Business Media LLC; 2018; doi: 10.1038/sdata.2018.68.

8. Wasimuddin, Schlaeppi K, Ronchi F, Leib SL, Erb M, Ramette A. Evaluation of primer pairs for microbiome profiling from soils to humans within the One Health framework. *Mol Ecol Resour*. 2020; doi: 10.1111/1755-0998.13215.

9. Zhang W, Fan X, Shi H, Li J, Zhang M, Zhao J, et al.. Comprehensive Assessment of 16S rRNA Gene Amplicon Sequencing for Microbiome Profiling across Multiple Habitats. Li D, editor. *Microbiol Spectr*. 2023; doi: 10.1128/spectrum.00563-23.

10. Brooks JP, Edwards DJ, Harwich MD, Rivera MC, Fettweis JM, Serrano MG, et al.. The truth about metagenomics: quantifying and counteracting bias in 16S rRNA studies. *BMC Microbiol*. Springer Science and Business Media LLC; 2015; doi: 10.1186/s12866-015-0351-6.

11. Abdill RJ, Graham SP, Rubinetti V, Ahmadian M, Hicks P, Chetty A, et al.. Integration of 168,000 samples reveals global patterns of the human gut microbiome. *Cell*. Elsevier BV; 2025; doi: 10.1016/j.cell.2024.12.017.

12. Jurburg SD. Short Read Lengths Recover Ecological Patterns in 16S rRNA Gene

Amplicon Data. *Mol Ecol Resour*. 2025; doi: 10.1111/1755-0998.14102.

13. Yang B, Wang Y, Qian P-Y. Sensitivity and correlation of hypervariable regions in 16S rRNA genes in phylogenetic analysis. *BMC Bioinformatics*. 2016; doi: 10.1186/s12859-016-0992-y.

14. Baker GC, Smith JJ, Cowan DA. Review and re-analysis of domain-specific 16S primers. *J Microbiol Methods*. 2003; doi: 10.1016/j.mimet.2003.08.009.

15. Wang Y, Qian P-Y. Conservative Fragments in Bacterial 16S rRNA Genes and Primer Design for 16S Ribosomal DNA Amplicons in Metagenomic Studies. Field D, editor. *PLoS ONE*. 2009; doi: 10.1371/journal.pone.0007401.

16. Callahan BJ, McMurdie PJ, Holmes SP. Exact sequence variants should replace operational taxonomic units in marker-gene data analysis. *ISME J*. 2017; doi: 10.1038/ismej.2017.119.

17. Parada AE, Needham DM, Fuhrman JA. Every base matters: assessing small subunit rRNA primers for marine microbiomes with mock communities, time series and global field samples: Primers for marine microbiome studies. *Environ Microbiol*. 2016; doi: 10.1111/1462-2920.13023.

18. Gonçalves RS, Musen MA. The variable quality of metadata about biological samples used in biomedical experiments. *Sci Data*. 2019; doi: 10.1038/sdata.2019.21.

19. Karsch-Mizrachi I, Nakamura Y, Cochrane G, on behalf of the International Nucleotide Sequence Database Collaboration. The International Nucleotide Sequence Database Collaboration. *Nucleic Acids Res*. 2012; doi: 10.1093/nar/gkr1006.

20. Leinonen R, Sugawara H, Shumway M, on behalf of the International Nucleotide Sequence Database Collaboration. The Sequence Read Archive. *Nucleic Acids Res*. 2011; doi: 10.1093/nar/gkq1019.

21. Chen S, Zhou Y, Chen Y, Gu J. fastp: an ultra-fast all-in-one FASTQ preprocessor. *Bioinformatics*. 2018; doi: 10.1093/bioinformatics/bty560.

22. Rognes T, Flouri T, Nichols B, Quince C, Mahé F. VSEARCH: a versatile open source tool for metagenomics. *PeerJ*. 2016; doi: 10.7717/peerj.2584.

23. Katoh K. MAFFT: a novel method for rapid multiple sequence alignment based on fast Fourier transform. *Nucleic Acids Res*. 2002; doi: 10.1093/nar/gkf436.

24. Brosius J, Palmer ML, Kennedy PJ, Noller HF. Complete nucleotide sequence of a 16S ribosomal RNA gene from Escherichia coli. *Proc Natl Acad Sci USA*. 1978; doi: 10.1073/pnas.75.10.4801.

25. Chen C, Liaw A, Breiman L. Using random forest to learn imbalanced data. *Univ Calif Berkeley*. 110:242004;

26. Saito T, Rehmsmeier M. The Precision-Recall Plot Is More Informative than the ROC Plot When Evaluating Binary Classifiers on Imbalanced Datasets. Brock G, editor. *PLOS ONE*. 2015; doi: 10.1371/journal.pone.0118432.

27. Mitra A, Skrzypczak M, Ginalski K, Rowicka M. Strategies for Achieving High Sequencing Accuracy for Low Diversity Samples and Avoiding Sample Bleeding Using Illumina Platform. Oudejans C, editor. *PLOS ONE*. 2015; doi: 10.1371/journal.pone.0120520.

28. Callahan BJ, McMurdie PJ, Rosen MJ, Han AW, Johnson AJA, Holmes SP. DADA2: High-resolution sample inference from Illumina amplicon data. *Nat Methods*. 2016; doi: 10.1038/nmeth.3869.

29. Das S, Biswas NK, Basu A. Mapinsights: deep exploration of quality issues and error profiles in high-throughput sequence data. *Nucleic Acids Res*. 2023; doi: 10.1093/nar/gkad539.

30. Gilbert JA, Jansson JK, Knight R. The Earth Microbiome project: successes and aspirations. *BMC Biol*. 2014; doi: 10.1186/s12915-014-0069-1.

31. Varliero G, Lebre PH, Stevens MI, Czechowski P, Makhalanyane T, Cowan DA. The use of different 16S rRNA gene variable regions in biogeographical studies. *Environ Microbiol Rep*. 2023; doi: 10.1111/1758-2229.13145.

32. Jurburg S, et al.,. Microbial Community Database (MiCoDa). v1 [Database]. 2023; doi: 10.25829/na76.

33. Jurburg SD, Álvarez Blanco MJ, Chatzinotas A, Kazem A, König-Ries B, Babin D, et al.. Datathons: fostering equitability in data reuse in ecology. *Trends Microbiol*. 2024; doi: 10.1016/j.tim.2024.02.010.

34. R Core Team. R: A language and environment for statistical computing. R Foundation for Statistical Computing, Vienna, Austria. *Httpwww R-Proj Org*. 2016;

35. Karsch-Mizrachi I, Arita M, Burdett T, Cochrane G, Nakamura Y, Pruitt KD, et al.. The international nucleotide sequence database collaboration (INSDC): enhancing global participation. *Nucleic Acids Res*. Oxford University Press; 2025; doi: 10.1093/nar/gkae1058.

36. Crandall ED, Toczydlowski RH, Liggins L, Holmes AE, Ghoojaei M, Gaither MR, et al.. Metadata preservation and stewardship for genomic data is possible, but must happen now. *bioRxiv*. Cold Spring Harbor Laboratory; 2022; doi: 10.1101/2022.09.12.507034.

37. Ebou A, Koua D, Zeze A. HyperEx: A Tool to Extract Hypervariable Regions from 16S rRNA Sequencing Data. *bioRxiv*. 2021; doi: 10.1101/2021.09.03.455391.

38. Bolyen E, Rideout JR, Dillon MR, Bokulich NA, Abnet CC, Al-Ghalith GA, et al..

Reproducible, interactive, scalable and extensible microbiome data science using QIIME 2. *Nat Biotechnol*. Nature Publishing Group US New York; 37:852–72019;

39. Langmead B, Salzberg SL. Fast gapped-read alignment with Bowtie 2. *Nat Methods*. Nature Publishing Group; 9:357–92012;

40. Li H, Handsaker B, Wysoker A, Fennell T, Ruan J, Homer N, et al.. The sequence alignment/map format and SAMtools. *bioinformatics*. Oxford University Press; 25:2078–92009;

41. Edgar RC. Search and clustering orders of magnitude faster than BLAST. *Bioinformatics*. Oxford University Press; 26:2460–12010;

42. Shade A, Dunn RR, Blowes SA, Keil P, Bohannan BJM, Herrmann M, et al.. Macroecology to Unite All Life, Large and Small. *Trends Ecol Evol*. 2018; doi: 10.1016/j.tree.2018.08.005.

43. Jones CB, White JR, Ernst SE, Sfanos KS, Peiffer LB. Incorporation of Data From Multiple Hypervariable Regions when Analyzing Bacterial 16S rRNA Gene Sequencing Data. *Front Genet*. 2022; doi: 10.3389/fgene.2022.799615.

44. Graham AS, Patel F, Little F, Van Der Kouwe A, Kaba M, Holmes MJ. Using short-read 16S rRNA sequencing of multiple variable regions to generate high-quality results to a species level. *Front Bioinforma*. 2025; doi: 10.3389/fbinf.2025.1484113.

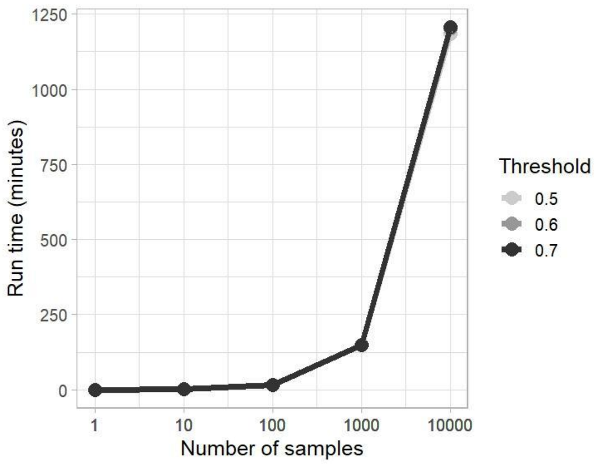

Figure 1. Relationship between the number of samples and run time (in minutes) using 8 GB of RAM and 4 CPU cores. We randomly selected sample numbers from the Earth Microbiome Project (Dataset 1), MiCoDa V1 (Dataset 2), and Datathon activities (Dataset 4). All samples were downloaded from the NCBI.

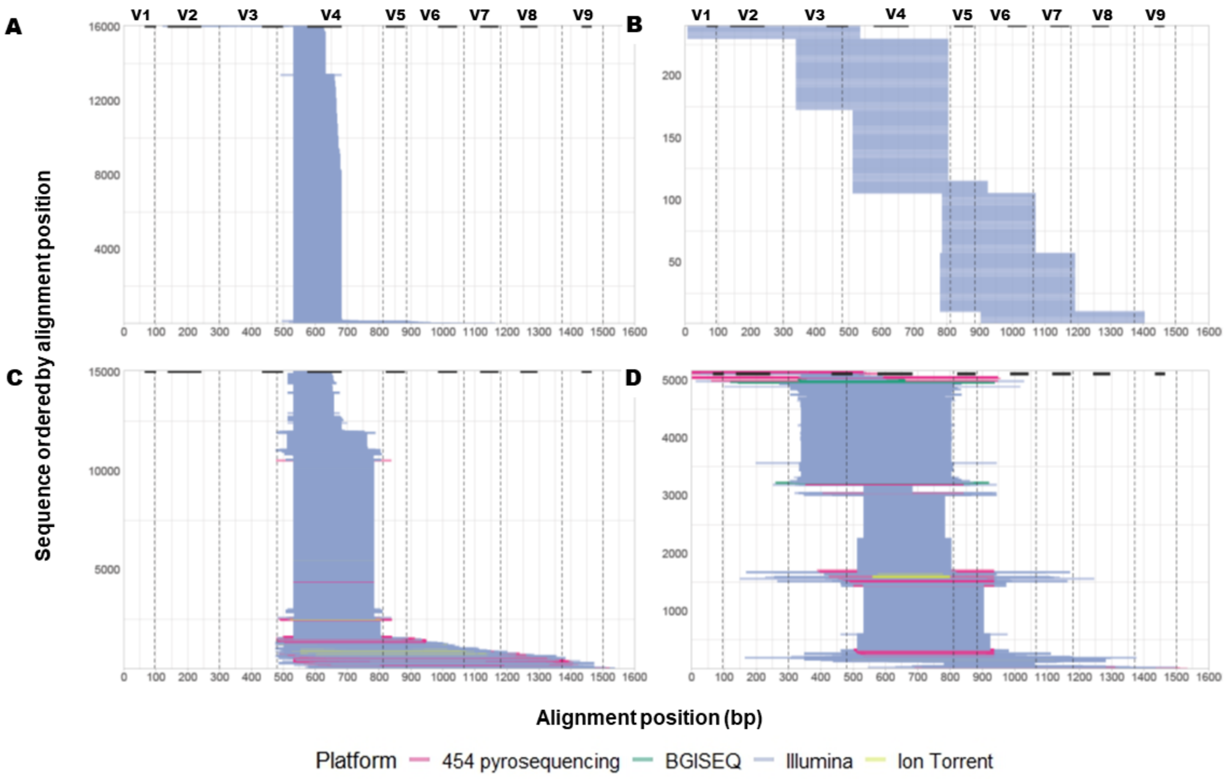

Figure 2. Alignment positions across 16S rRNA regions and sequencing setups. a) Same 16S rRNA region and sequencing setup (N = 16059 samples); b) Different 16S rRNA regions, same sequencing setup (N = 239 samples); c) Same 16S rRNA region, different sequencing setups (N = 15049 samples); d) Different 16S rRNA regions and sequencing setups (N = 5113 samples).The upper part of the figure, along with the dashed lines, indicates the start and end positions of the sequencing setups used to assign specific regions of the 16S rRNA gene (modified from Yang *et al.,* 2016). The hypervariable regions corresponding to each setup are highlighted with bold black bars (Brosius *et al.,* 1978).

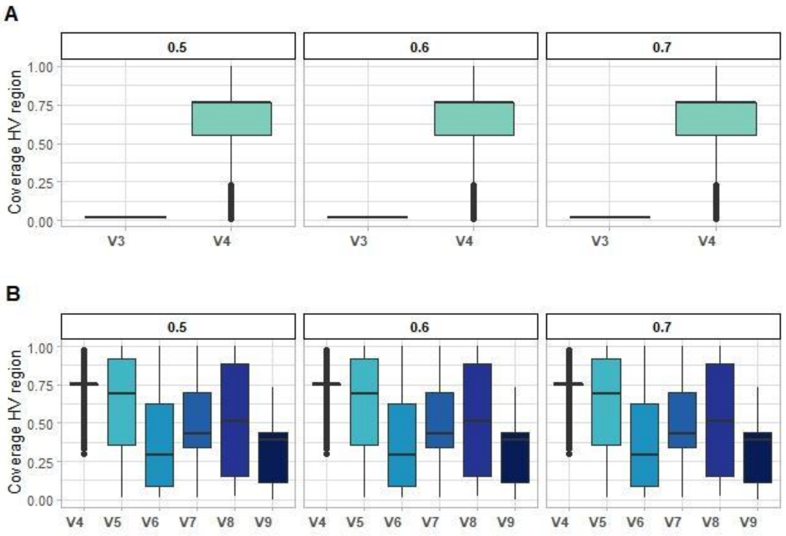

Figure 3. Differences in 16S rRNA gene coverage using the same primer set (Primer 515R-806R for V4 region) but different sequencing setups. A) Predicted 16S rRNA region coverage start, B) Predicted 16s rRNA region coverage end.

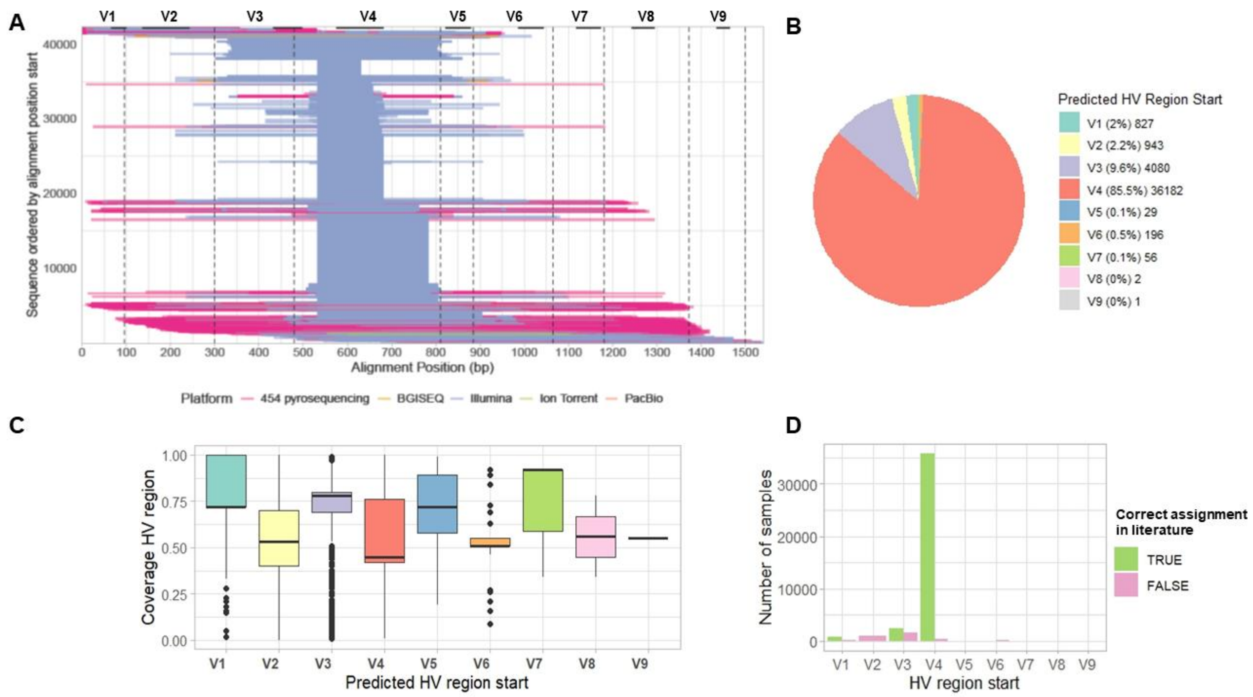

Figure 4. Application of HVRLocator for the selection of V4 16S rRNA gene metabarcoding samples from MiCoDa V2. A) Alignment start positions across the 16S rRNA gene for the 42,316 samples analyzed, B) Percentage of samples retained for downstream analyses after applying the HVRLocator tool, C) Variation in gene coverage across sequences, D) Number of samples in which primer assignment matched the metadata.

Supplementary Figure S1

Click here to access/download
Supplementary Material
Supplementary Figure S1.pdf

Click here to access/download

Supplementary Material

TableS1_Run_accession_list_with_primers_Random_For
est_model.csv

TableS2

Click here to access/download
Supplementary Material
TableS2.csv

Click here to access/download
Supplementary Material
TableS3_Random_Forest_Model_for_Primer_Presence_
Prediction.pdf

Click here to access/download
Supplementary Material
TableS4_Dataset1_EarthMicrobiomeProject.csv

Click here to access/download
Supplementary Material
TableS4_Dataset2_MiCoDaVersion1.csv

TableS4_Dataset3

Click here to access/download
Supplementary Material
TableS4_Dataset3_WasimuddinVarliero.csv

Click here to access/download
Supplementary Material
TableS4_Dataset4_Datathons.csv

Click here to access/download
Supplementary Material
TableS5_RunTime.pdf

Click here to access/download
Supplementary Material
TableS6_Run_accession_list_for_case_study.csv

TableS7_CaseStudyOutput

Click here to access/download
Supplementary Material
TableS7_CaseStudyOutput.pdf

Click here to access/download
Supplementary Material
TableS8_CorrectAssignmentInLiterature.csv

Personal Cover
Click here to access/download;Personal Cover;PersonalCover_GIGA-D-25-00344.pdf

GIGA-D-25-00344
HVRLocator: A Computationally Efficient Tool for Identifying Hypervariable Regions in Large 16S rRNA Datasets
Clara Arboleda-Baena; Felipe Borim Correa; Joao Pedro Saraiva; Santiago Castillo-Rivadeneira; Jonas Coelho Kasmanas; Antonis Chatzinotas; Stephanie D. Jurburg
GigaScience

Leipzig, 17 February 2026

Dear Dongni Ma,

I hope this message finds you well.

Please find attached our response to the reviewers regarding manuscript GIGA-D-25-00344: HVRLocator: A Computationally Efficient Tool for Identifying Hypervariable Regions in Large 16S rRNA Datasets. We have addressed all comments. We would like to thank you and the reviewers for the constructive feedback, which has helped us improve the manuscript and clarify our message. We hope that the revisions satisfactorily address the minor revision requests.

As outlined in the response document, it was not necessary to register a new software application in the bio.tools or SciCrunch.org databases to obtain RRID (Research Resource Identification Initiative ID) or biotoolsID identifiers.

We have now also uploaded the scripts used in our analyses to GitHub, as suggested by one of the reviewers.

We look forward to your response.

Best wishes,

Dr. Clara Arboleda
On behalf of all co-authors

GIGA-D-25-00344
HVRLocator: A Computationally Efficient Tool for Identifying Hypervariable Regions in Large 16S rRNA Datasets
Clara Arboleda-Baena; Felipe Borim Correa; Joao Pedro Saraiva; Santiago Castillo-Rivadeneira; Jonas Coelho Kasmanas; Antonis Chatzinotas; Stephanie D. Jurburg
GigaScience

Leipzig, 17 February 2026

Dear Dongni Ma,

I hope this message finds you well.

Please find attached our response to the reviewers regarding manuscript GIGA-D-25-00344: HVRLocator: A Computationally Efficient Tool for Identifying Hypervariable Regions in Large 16S rRNA Datasets. We have addressed all comments. We would like to thank you and the reviewers for the constructive feedback, which has helped us improve the manuscript and clarify our message. We hope that the revisions satisfactorily address the minor revision requests.

As outlined in the response document, it was not necessary to register a new software application in the bio.tools or SciCrunch.org databases to obtain RRID (Research Resource Identification Initiative ID) or biotoolsID identifiers.

We have now also uploaded the scripts used in our analyses to GitHub, as suggested by one of the reviewers.

We look forward to your response.

Best wishes,

Dr. Clara Arboleda
On behalf of all co-authors

Reviewer reports:

Reviewer #1: Metabarcoding data are accumulating rapidly. This paper makes a very valuable contribution to the automated extraction and curation of metabarcoding data and should be of great value in facilitating the re-use of existing data and the construction of custom databases based on these.
I have not tested or tried to install the software myself, as the manuscript provided sufficient detail to enable me to assess the tool

R/ We thank the reviewer for their positive feedback.

General comments:

The manuscript is written entirely in terms of "bacteria" and aligns amplicons to an E. coli model sequence. This is reasonable, but there should certainly be some acknowledgement of Archaea and ideally some mention of Eukaryotes too. These are probably things for the discussion section of this manuscript, but the authors may wish to consider whether a future version of the program could contain options to use model Archaea and Eukaryote sequences as alternatives to the E. coli model.

R/ Thank you for the comment. We acknowledge that this software may be useful for researchers working with metabarcoding data from other realms of life. This is foreseen by the software, which allows the user to input their own reference sequence and map against it, offering that flexibility. We now refer to this in Lines 389-393. While this manuscript focuses on the evaluation of *E. coli* for usage against 16S rRNA gene data, we are currently benchmarking its use for fungi (ITS region) for future versions, although this is beyond the scope of the present work. For clarity, we have added the following text to the manuscript:

Lines 389-393: "Although the present work focuses on bacteria, we acknowledge that other domains of life, including Archaea and Eukaryotes, are relevant for future meta-analyses and synthesis studies. Consequently, future versions of the program aim to incorporate alternative model sequences representing Archaea and Eukaryota."

It would also be helpful to assess how the program with its E. coli model deals with sequence data from Archaea, Eukaryotes (including mitochondria) and bacteria that are very divergent from E. coli.

R/ Thank you for the comment. Even for bacterial taxa that are highly divergent from E. coli, the 16S rRNA gene remains highly conserved across Bacteria, ensuring sufficient sequence similarity for reliable alignment to the appropriate reference region. Although 16S rRNA sequences vary in length among taxa, our validation showed 99% agreement between the predicted hypervariable regions and those reported in the original publications (metadata or NCBI records). Together, the conserved structure of the 16S rRNA gene and the strong empirical validation support the conclusion that the current alignment strategy provides reliable estimates of start and end

positions for hypervariable regions in Bacteria and adequately addresses the objectives of this study.

Regarding Archaea and Eukaryotes, we performed several tests and identified potential warning signs that may alert users to review the metadata of sequences exhibiting the following patterns:

1. Putative ITS region: Sequences showing coverage across more than four or five hypervariable (HV) regions. In such cases, users should verify the following information in the metadata: the sequencing platform. If the platform used for these sequences is not PacBio or Nanopore, the primers may be targeting regions that are not specific to bacteria. If the mentioned platforms were used, users should also verify that the primers are not targeting Archaea or Eukaryotes.

The following table presents examples of ITS regions analyzed using HVRLocator:

Sample_ID	Primer_Presence	Score_Primer_Presence	Min_Alignment_start	Max_Alignment_end	Average_Alignment_start	Average_Alignment_end	Median_Alignment_start	Median_Alignment_end	d_HV_regi	d_HV_reg	ased_HV_	ased_HV_	Coverage_HV_region_start	Coverage_HV_region_end	Warnings
SRR2428[illegible]816	FALSE	0.17	0	1540	378	1068	399	1148	V3	V7	V4	V7	0.45	0.72	V3 below threshold of 0.6
SRR2428[illegible]817	FALSE	0.17	0	1540	398	992	399	1110	V3	V7	V4	V6	0.45	0.39	V3 and V7 below threshold of 0.6
SRR2428[illegible]818	FALSE	0.17	0	1540	400	1018	399	1110	V3	V7	V4	V6	0.45	0.39	V3 and V7 below threshold of 0.6
SRR2428[illegible]819	FALSE	0.17	0	1540	402	994	399	1110	V3	V7	V4	V6	0.45	0.39	V3 and V7 below threshold of 0.6
SRR2428[illegible]820	FALSE	0.17	0	1540	446	1017	399	1110	V3	V7	V4	V6	0.45	0.39	V3 and V7 below threshold of 0.6
SRR2428[illegible]821	FALSE	0.16	0	1540	431	1000	399	1110	V3	V7	V4	V6	0.45	0.39	V3 and V7 below threshold of 0.6
SRR2428[illegible]822	FALSE	0.17	0	1540	378	953	399	1110	V3	V7	V4	V6	0.45	0.39	V3 and V7 below threshold of 0.6
SRR2428[illegible]823	FALSE	0.17	0	1540	392	961	399	1010	V3	V6	V4	V6	0.45	0.69	V3 below threshold of 0.6
SRR2428[illegible]824	FALSE	0.17	0	1540	392	968	399	953	V3	V6	V4	V5	0.45	0.38	V3 and V6 below threshold of 0.6
SRR2428[illegible]825	FALSE	0.17	0	1540	401	963	399	953	V3	V6	V4	V5	0.45	0.38	V3 and V6 below threshold of 0.6
SRR2560[illegible]816	FALSE	0.17	0	1540	357	872	186	826	V2	V5	V3	V4	0.56	0.2	V2 and V5 below threshold of 0.6
SRR2560[illegible]819	FALSE	0.2	0	1540	690	1172	903	1450	V6	V9	V6	V8	0.91	0.34	V9 below threshold of 0.6
SRR2560[illegible]821	FALSE	0.22	0	1540	425	956	186	826	V2	V5	V3	V4	0.56	0.2	V2 and V5 below threshold of 0.6
SRR2560[illegible]824	FALSE	0.2	0	1540	627	1210	685	1210	V4	V8	V5	V7	0.38	0.16	V4 and V8 below threshold of 0.6
SRR2560[illegible]826	FALSE	0.22	0	1540	602	1118	574	1048	V4	V6	V4	V6	0.72	0.91	
SRR2560[illegible]828	FALSE	0.2	0	1540	303	1164	177	1096	V2	V7	V2	V6	0.61	0.27	V7 below threshold of 0.6
SRR2560[illegible]830	FALSE	0.17	0	1540	600	1189	529	1048	V4	V6	V4	V6	0.85	0.91	
SRR2560[illegible]832	FALSE	0.21	0	1540	449	970	186	826	V2	V5	V3	V4	0.56	0.2	V2 and V5 below threshold of 0.6
ERR5924913	FALSE	0.17	0	1540	482	1055	548	1111	V4	V7	V4	V6	0.8	0.4	V7 below threshold of 0.6
ERR5924916	FALSE	0.17	0	1540	463	1076	595	1177	V4	V7	V4	V7	0.65	0.97	
ERR5924917	FALSE	0.17	0	1540	587	1165	595	1257	V4	V8	V4	V7	0.65	0.4	V8 below threshold of 0.6
ERR5924918	FALSE	0.17	0	1540	536	1123	595	1180	V4	V7	V4	V7	0.65	1	
ERR5924921	FALSE	0.17	0	1540	445	1009	411	984	V3	V6	V4	V5	0.38	0.33	V3 and V6 below threshold of 0.6
ERR5924922	FALSE	0.17	0	1540	385	1004	411	984	V3	V6	V4	V5	0.38	0.33	V3 and V6 below threshold of 0.6
ERR5924923	FALSE	0.17	0	1540	452	1039	548	984	V4	V6	V4	V5	0.8	0.33	V6 below threshold of 0.6
ERR5924933	FALSE	0.18	0	1540	466	1053	595	1177	V4	V7	V4	V7	0.65	0.97	
ERR5924934	FALSE	0.17	0	1540	494	1088	595	1257	V4	V8	V4	V7	0.65	0.4	V8 below threshold of 0.6
ERR5924935	FALSE	0.17	0	1540	429	1022	469	1111	V3	V7	V4	V6	0.06	0.4	V3 and V7 below threshold of 0.6
ERR5924937	FALSE	0.17	0	1540	401	1012	548	1111	V4	V7	V4	V6	0.8	0.4	V7 below threshold of 0.6

2. Putative Archaea or 18S hypervariable region: As clarified in the manuscript, users must ensure that the target domain is Bacteria. Due to similarities in gene length and conserved regions, sequences targeting archaeal 16S rRNA genes or eukaryotic 18S rRNA genes may still produce a prediction when analyzed with HVRLocator. However, the tool is specifically designed and validated for bacterial 16S rRNA sequences. Therefore, verification of the sample metadata is essential to confirm that the predicted hypervariable region corresponds to bacterial sequences.

Finally, we have included the following statement in the main text to clarify this point for users:

Lines 275-283: “Importantly, validation highlighted HVRLocator tool’s ability to identify problematic sequences. For example, we observed 932 samples with abnormally long average sequence lengths (i.e., >600 bp) that exceeded the expected output lengths with Illumina platforms. Upon manually reviewing these sequences, we found that either the sequencing platform was incorrectly annotated in the metadata (NCBI or the associated publication), or the sequences did not correspond to the 16S rRNA gene but rather to the Internal Transcribed Spacer (ITS) region or the nifH gene. This highlights the use of HVRLocator as a curation tool for large datasets, where human errors in annotation can significantly impact downstream analysis.”

Lines 389-393: “Although the present work focuses on bacteria, we acknowledge that other domains of life, including Archaea and Eukaryotes, are relevant for future meta-analyses and synthesis studies. Consequently, future versions of the program aim to incorporate alternative model sequences representing Archaea and Eukaryota”

The methods section does not contain details of software used to generate the figures, or whether these figures are produced by "the pipeline" or by separate analysis of the .txt file that the pipeline produces. I suspect that it is that latter, in which case making the authors should make the scripts used available - as well as providing complete documentation of what has been done, this is likely to increase use made of the tool.
And it would be helpful to include an output file in the supplementary materials.

R/ Thank you for the recommendation. We have now made all scripts and tables available on GitHub. The following text has been included in the Methods section:

“All graphics were carried out in R with RStudio interface (R Core Team, 2016), and all pipelines are available at: https://github.com/ClarArboledaBaena/HVRLocator-Figures.”

Specific comments

Line 64 "however the integration of these data in light of processing metadata" - not clear

R/ Thank you for noting this. The correct phrase is: “however, the integration of these data in the context of Big Data processing and synthesis”.

Line 67-8 "though bacterial diversity increases linearly with amplicon length".
Needs re-wording. The number of ASVs will increase with amplicon length, but the actual bacterial diversity in a sample is constant.

R/ Thank you for your comment. We have modified it to “though detected bacterial diversity increases linearly with amplicon length”.

Line 79 "Wasimuddin and colleagues" should be "Wasimuddin et al". More generally, check that citations conform with journal house style

R/ Done

Line 79-82 "For example, Wasimuddin and colleagues [8] found that compared to three other primer sets targeting different regions, the primer pair targeting the V4 hypervariable region of the 16S rRNA gene produced the highest estimates of species richness and diversity across various sample types"
There are three issues here:
1) different primer pairs vary in their coverage and bias, so different primers targeting the same variable region will produce different numbers of ASVs
2) Even with complete coverage and the absence of bias, different variable regions will generate different numbers of ASVs as a result of differences in length and rate of evolution between variable regions (and differences in the number of ASVs that are clustered into OTUs at a particular sequence similarity threshold

3) The relationship between ASVs or OTUs and "species" is not straightforward (Edgar, 2018). At minimum "species" should be replace with ASV or OTU (whichever Wasimuddin et al used) Edgar, R. C. (2018). Updating the 97% identity threshold for 16S ribosomal RNA OTUs. Bioinformatics, 34(14), 2371-2375. doi:10.1093/bioinformatics/bty113

R/ Yes, the reviewer is correct regarding all three points. Accordingly, we have replaced the term “species” with “Amplicon Sequence Variants (ASVs)” and added the following sentence:

Lines 83-85: “However, different primers targeting the same variable region can still generate different numbers of ASVs (Parada et al., 2016)”

Line 89-90 "as bacterial diversity and taxonomic resolution linearly increase with target sequence length [12]." Overlaps with statements made in line 67-8, and the same issue applies here.

R/ Thank you for pointing this out. We agree that this statement overlaps with the one in lines 67–68. To avoid redundancy, we have rephrased lines 89–90 to better differentiate the concepts and improve clarity:

“Variation in amplicon length complicates the reuse of 16S rRNA metabarcoding data, since longer sequences tend to detect greater bacterial diversity and taxonomic resolution”

Lines 167-170. The output file contains (amongst other things) "Predicted HV region Start/End: Predicted hypervariable (HV) region based on the median alignment start and end positions across all reads, inferred from literature on conserved and hypervariable regions of the 16S rRNA gene (Brosius et al., 1978; Yang et al., 2016)". This implies that the program predicts a single variable region for each study. I am not clear what this column will contain for amplicons that contain more than one variable region, although columns 11-19 indicate that the program identifies the presence/absence of each of the 9 HV regions.

R/ Thank you very much for this helpful comment. For clarification, HVRLocator predicts a single hypervariable region for each sample, rather than for each study, as mentioned by the reviewer. As correctly pointed out, some studies (BioProjects) include samples spanning multiple hypervariable regions, a situation that has become increasingly common with the advent of long-read sequencing technologies.

In addition to identifying the hypervariable region corresponding to the average and median start and end positions of the amplicons within each sample, HVRLocator also reports the coverage across all nine hypervariable regions for every individual sample. In this way, users can evaluate the amplicon length, its exact start and end positions, and the coverage of specific hypervariable region(s) within each sample.

We have added the following sentence in the main manuscript to clarify this point for users (Lines 201-205): ”Finally, in addition to identifying the hypervariable region based on the average and

median start and end positions of the amplicons within each sample, HVRLocator also reports coverage across all nine hypervariable regions for every individual sample. This allows users to assess the amplicon length, determine its exact start and end positions, and evaluate the coverage of specific hypervariable region(s) within each sample."

Regarding studies that include samples with different hypervariable region coverage, we aimed to illustrate this scenario using Dataset B, which comprises 242 samples from two studies that compared different primer sets but were sequenced on the same platform (Wasimuddin et al., 2020; Varliero et al., 2023). As shown in Figure 2B, HVRLocator successfully predicts different variable regions within the same study.

My guess is that the authors are using "HV region" in two different sense:

1) Its usual meaning of one region out of V1 to V9
2) The sequence from the beginning of the first of the nine variable regions the amplicon includes to the end of the last.

It would also be helpful to indicate whether the sequence positions here are relative to the E coli model or refer to sequence positions in the amplicon

R/ Thank you for the comment. For clarification, we use the term "HV region" according to option (1), that is, its standard meaning as one of the regions V1–V9. To address the reviewer's concern, we will provide an example illustrating the HVRLocator output and demonstrate how the information produced by the tool resolves the points raised:

The following table shows the HVRLocator output for five samples from the same study (Varliero et al., 2023). As shown, even within the same study, the tool predicts different HV regions for different samples.

Output (only 8 columns shown):

Sample_ID	Primer_Presence	Score_Primer_Presence	Average_Alignment_start	Average_Alignment_end	Median_Alignment_start	Median_Alignment_end	Predicted_HV_region_start	Predicted_HV_region_end
ERR10042760	FALSE	0.17	8	537	7	534	V1	V4
ERR10042770	FALSE	0.17	340	804	339	803	V3	V4
ERR10042780	FALSE	0.17	512	806	513	804	V4	V4
ERR10042790	FALSE	0.17	510	923	513	924	V4	V6
ERR10042800	FALSE	0.17	898	1402	905	1404	V6	V9

We are going to explain the first sample:

For sample ERR10042760, the average and median alignment start positions are 8 and 7, respectively. These values represent the sequence start positions relative to the E. coli reference model, as mentioned in your comment. As you can see the predicted HV region where this amplicon starts for this sample is V1.

The average and median alignment end positions are 537 and 534, respectively. These values indicate the sequence end positions relative to the E. coli reference model. The predicted hypervariable (HV) region at which this amplicon ends is V4.

This indicates that the samples cover HV regions V1, V2, V3, and V4. This can be verified by examining the remaining columns of the output.

Output (only 14 columns shown):

Sample_ID	Coverage_based_HV_region_start	Coverage_based_HV_region_end	Coverage_HV_region_start	Coverage_HV_region_end	Warnings	Cov_V1	Cov_V2	Cov_V3	Cov_V4	Cov_V5	Cov_V6	Cov_V7	Cov_V8	Cov_V9
ERR10042760	V1	V3	0.93	0.17	V4 below threshold of 0.5	0.93	1	1	0.17	0	0	0	0	0
ERR10042770	V3	V4	0.78	0.98	NA	0	0	0.78	0.98	0	0	0	0	0
ERR10042780	V4	V4	0.88	0.88	NA	0	0	0	0.88	0	0	0	0	0
ERR10042790	V4	V5	0.9	0.22	V6 below threshold of 0.5	0	0	0	0.9	1	0.22	0	0	0
ERR10042800	V6	V8	0.89	0.14	V9 below threshold of 0.5	0	0	0	0	0	0.89	1	1	0.14

The tool further shows that the amplicon provides full coverage of HV regions V1 to V3. This is reflected in the columns “Cov_V1” to “Cov_V9,” which report coverage values (ranging from 0 to 1) for each HV region. Coverage values close to or equal to 1 are observed for HV regions V1–V3, whereas a lower value is observed for V4.

This indicates that sample ERR10042760 starts in HV region V1 and ends in HV region V4, fully covering HV regions V1–V3, while HV region V4 is only partially covered. This information allows users to interpret the output and make informed decisions in downstream analysis pipelines.

Reviewer #2: General Comments

This manuscript introduces a tool named HVRLocator, designed to address the issue of missing or non-standard metadata in 16S rRNA sequencing data found in public databases such as the SRA. The tool identifies amplicon regions by aligning sequences to a reference genome and attempts to detect the presence of primers using a machine learning model. This is a subject with significant practical value, particularly for conducting large-scale meta-analyses. However, there are still many issues regarding methodological rigor, the depth of validation, and comparisons with existing tools that require further clarification by the authors.

R/ We address the reviewer's concerns regarding methodological rigor, the depth of validation, and comparisons with existing tools in the following comments.

Major Comments

1. Concerns regarding the singularity of the reference sequence

A.The authors mention aligning sequences to a single Escherichia coli (J01859.1) reference genome to determine start and end positions. Is a single E. coli reference sufficient to cover Archaea or bacterial phyla that are distantly related to Proteobacteria, which may be present in environmental samples (e.g., soil, ocean)?

R/ HVRLocator aims to identify primers designed to target Bacteria only, not Archaea, although We acknowledge that bacterial primers amplify non-target 16S rRNA from Archaea. Crucially, this software is designed to work with metabarcoding data, where the composition of the sequenced community is usually unknown *a priori*, so it is possible and expected that when distantly-related taxa are present in the samples, some reads will not map to *E. coli* as well as others. This is why we designed HVRLocator to systematically download 10,000 reads per sample to assess the mapping of these reads against our model *E. coli* on average. We see no indication of the presence of distantly related organisms in a sample affecting our ability to infer the target region sequenced, as confirmed by our assessment of the region sequenced across the methods stated in the literature for a subset of our samples.

We acknowledge that the ability to change the mapping sequence is useful, and would greatly expand the functionality of HVRLocator, and have built the tool foreseeing this expansion in the future, and we know acknowledge this in Line 389-393, although the accuracy against other mapping sequences is beyond the scope of this paper. Indeed, we are currently testing it for fungal studies targeting the ITS region, and hope to release this new version in the coming years, after rigorous testing.

Lines 389-393: "Although the present work focuses on bacteria, we acknowledge that other domains of life, including Archaea and Eukaryotes, are relevant for future meta-analyses and synthesis studies. Consequently, future versions of the program aim to incorporate alternative model sequences representing Archaea and Eukaryota."

B. For taxa with significant length variations or insertions/deletions (Indels), could forced alignment to the E. coli reference lead to misjudgment of start/end positions?

R/ We do not consider length variation to be a major issue, as HVRLocator aligns relatively short amplicons to the full-length E. coli 16S rRNA reference sequence. Although some taxa exhibit insertions/deletions (indels) or length variation, the 16S rRNA gene is highly conserved across Bacteria, ensuring sufficient sequence similarity for reliable alignment to the appropriate reference region.

While 16S rRNA sequences vary in length across taxa, our validation showed 99% agreement between predicted hypervariable regions and those reported in the original publications (metadata or NCBI records). Together, the conserved structure of the 16S rRNA gene and the strong empirical validation support that the current alignment strategy provides reliable start and end position estimates for hypervariable regions and adequately addresses the objectives of this study.

C. Have the authors evaluated the impact on accuracy if a more universal reference database (such as representative sequences from SILVA or Greengenes) were used?

R/ We did not evaluate the impact of using a more universal reference database, as the current approach yielded accurate, consistent, and importantly, efficient results for the purposes of this study. However, we agree that exploring the use of representative sequences from broader reference databases such as SILVA or Greengenes could be valuable, and we will consider this for future versions of the tool.

2. Rationality of the primer detection model (Random Forest based on Quality Scores)

2.1 The authors developed a Random Forest model to predict primer presence by analyzing the quality score distribution of the first 1,000 reads. Primer detection is typically based on the sequence itself rather than quality scores. Can the authors explain why quality scores were chosen as features?

R/ We appreciate the reviewer's point and welcome the opportunity to clarify our rationale. Illumina sequencing is known to generate distinct quality score patterns in the first few cycles when base diversity is low, as occurs when untrimmed primers are present at the start of all reads (Mitra et al., 2015). Thus, we chose to use quality scores as features because they serve as a practical proxy for detecting primer presence. This approach allows for accurate, sequence-agnostic primer detection, especially when the actual primer sequences used are unknown or highly variable across datasets, which is the case in synthesis work, and a key reason for our development of HVRLocator. We have added text in lines 139-145 in the manuscript indicating our rationale:

Lines 139-145: "RF performs well under moderate class imbalances when class-aware evaluation and sampling is applied [25,26]. Additionally, distinct quality score patterns are known to occur in the first few cycles of Illumina sequencing when base diversity is low [27] as is in the case of untrimmed primers at the start of reads. Quality score patterns have also been used to detect sequencing bias and artifacts by tools such as DADA2 [28] and Mapinsights [29]. Thus, this metric can serve as a proxy for detecting primer presence."

Mitra A. et al. (2015). “Strategies for Achieving High Sequencing Accuracy for Low Diversity Samples…”

2.2 Sequencing quality scores are influenced by technical factors such as sequencer status, reagent batches, and run cycles, which have no direct biological correlation with the presence of primers. Is there a risk that this model is "overfitting" specific sequencing platforms or datasets?

R/ This is a valid concern, and we took several steps to mitigate the risk of platform-specific overfitting. First, our training dataset included thousands of SRA runs generated from a range of sequencing platforms and protocols. Second, during validation, we tested the model across multiple independent datasets, including those with differing primer sets and sequencing technologies, and achieved consistently high performance (precision and recall >99%) regardless of platform. Third, we ensured that the features used generalize well across datasets by using stratified sampling and fixed model parameters. Similar uses of quality score patterns to model sequencing bias and detect artifacts have been validated in tools like DADA2 (Callahan et al., 2016) and Mapinsights (Das et al., 2023), supporting the robustness of such approaches. Our observed performance across diverse conditions suggests the model is not overfitting. We have now added text in the manuscript (lines 139-145) highlighting the use of quality score patterns by publicly available tools:

Lines 139-145: “RF performs well under moderate class imbalances when class-aware evaluation and sampling is applied [25,26]. Additionally, distinct quality score patterns are known to occur in the first few cycles of Illumina sequencing when base diversity is low [27] as is in the case of untrimmed primers at the start of reads. Quality score patterns have also been used to detect sequencing bias and artifacts by tools such as DADA2 [28] and Mapinsights [29]. Thus, this metric can serve as a proxy for detecting primer presence.”

Das S. et al. (2023). “Mapinsights: deep exploration of quality issues and error profiles in high-throughput sequence data.”

Callahan B. et al. (2016). “DADA2: High resolution sample inference from Illumina amplicon data”

Since the reads are already downloaded, why not directly use degenerate primer sequence matching (e.g., using Cutadapt or SeqKit logic) to determine primer presence? This seems to be a more direct and accurate method.

R/ Degenerate sequence matching (e.g., via Cutadapt or SeqKit) requires prior knowledge of all potential primer sequences used across datasets. This poses a challenge for large-scale curation of public datasets, which often lack standardized or complete primer metadata, especially when INSDC databases often lack a link between the publication and the data for a substantial portion of the datasets archived. Our method provides a generalizable and lightweight solution that does not rely on prior knowledge of primer sequences (mentioned in lines 358-364 of the manuscript). By bypassing primer-specific matching, we reduce computational cost and avoid the risk of

missing primer variants due to sequence mismatches or degeneracy. We view our workflow as a complementary tool, particularly useful in high-throughput or poorly annotated datasets.

Line 358-364: “In contrast, HVRLocator does not rely on simulations or a priori primer information, and is designed for its application to existing datasets. HVRLocator operates directly on large, INSDC-archived metabarcoding datasets to identify the start and end positions of sequenced 16S rRNA amplicons, determine their corresponding hypervariable regions, and detect the presence primer sequences, generating the technical metadata that is needed for bioinformatics processing of the raw sequences.”

3. Verification of accuracy claims
In the validation section, the authors claim to achieve 100% accuracy on certain datasets. In bioinformatics tool development, a claim of 100% accuracy is often a red flag. Have the authors manually checked those samples marked as "correct" by the model that might suffer from edge effects or borderline cases?

R/ We fully agree that claims of 100% accuracy require scrutiny. This value refers to specific controlled datasets where the sequenced region and primer pairs were known from curated sources (e.g., MiCoDa). Regardless, we manually reviewed edge cases and anomalies flagged by our tool such as sequences with unusual read lengths or misannotated primer regions and confirmed that HVRLocator correctly identified true mismatches with the reported metadata. In several such cases, we found that the discrepancies arose from metadata errors rather than model misclassification. These actions are stated in lines 263-283 of the manuscript:

Lines 263-283: “To check the reliability of HVRLocator relative to manual extraction of metadata from the literature, we manually extracted data related to the primers used and the 16S rRNA HV region targets from all samples in dataset c. For a total of 18,426 samples, 16,771 samples were processed successfully without warnings; common issues included missing FASTQ files, low reads, alignment failures, and NCBI portal-related issues. Of the 16,771 samples processed, 1,712 (10%) did not produce results consistent with the literature (e.g. mismatches between the start region alignment and the reported primer, or incorrect HV region alignment compared with the reported HV region), underscoring the value of obtaining metadata from the sequence data directly, rather than from the literature. Finally, for the diverse data set that used both different 16S rRNA regions and sequencing setups (Figure 2d), HVRLocator accurately and rapidly assigned the alignment positions.
Importantly, validation highlighted HVRLocator tool’s ability to identify problematic sequences. For example, we observed 932 samples with abnormally long average sequence lengths (i.e., >600 bp) that exceeded the expected output lengths with Illumina platforms. Upon manually reviewing these sequences, we found that either the sequencing platform was incorrectly annotated in the metadata (NCBI or the associated publication), or the sequences did not correspond to the 16S rRNA gene but rather to the Internal Transcribed Spacer (ITS) region or the nifH gene. This highlights the use of HVRLocator as a curation tool for large datasets, where human errors in annotation can significantly impact downstream analysis.”

4. Dataset imbalance in the Random Forest model

For the Random Forest model, the authors used 882 samples with primers and 8,940 samples without primers for training. Such an extremely imbalanced dataset, even with stratified sampling, may cause the model to be biased towards the majority class.

R/ We thank the reviewer for this important point. We evaluated performance using class-specific metrics achieving a precision of 100% and recall of 99.55% for the "primer-present" class on the held-out test set. These figures demonstrate that the model retained strong sensitivity and specificity for the minority class despite the imbalance. Random Forests are known to perform robustly under moderate imbalance when class-aware evaluation and sampling are applied (Chen et al., 2004; Saito & Rehmsmeier, 2015). We have added text to manuscript in lines 139-145 that highlights this aspect of RF models. We also acknowledge that future versions could incorporate additional balancing techniques (e.g., class weighting), but the current results give confidence that no skew bias is affecting performance.

To confirm the robustness of the RF model, we applied the same stratified sampling during the 80/20 train-test split and ensured that both classes were adequately represented in each fold (lines 152-158 of the manuscript). This "balanced" model (which was trained using 881 no-primer samples and 881 primer samples) yielded virtually identical performance compared to the original: primer recall remained at 0.994, primer precision at 1.000, and overall accuracy exceeded 99.7%. This confirms that the initial model was not biased by the class imbalance and that the features used provide strong separation between classes. :

	precision	recall	f1-score	support
**no-primer**	0.994	1.000	0.997	177
**primer**	1.000	0.994	0.997	176
**accuracy**		0.997	0.997	0.997
**macro avg**	0.997	0.997	0.997	353
**weighted avg**	0.997	0.997	0.997	353

Lines 152-158: "The model was trained using scikit-learn's RandomForestClassifier (v1.2.1) with 100 estimators and a fixed random seed (random_state=42), using an 80/20 stratified train-test split. The Random Forest model yielded a precision of 99.96% for the dataset without primers and 100% for the dataset with primers. Recall of the model using the "no-primer" and "primer" dataset was 100% and 99.55%, respectively. Full details on the model generation including the algorithm, versions and packages are available in the Supplementary Table S3."

Lines 139-145: "RF performs well under moderate class imbalances when class-aware evaluation and sampling is applied [25,26]. Additionally, distinct quality score patterns are known to occur in the first few cycles of Illumina sequencing when base diversity is low [27] as is in the case of untrimmed primers at the start of reads. Quality score patterns have also been used to detect sequencing bias and artifacts by tools such as DADA2 [28] and Mapinsights [29]. Thus, this metric can serve as a proxy for detecting primer presence."

Chen, C., Liaw, A., & Breiman, L. (2004). Using random forest to learn imbalanced data. University of California, Berkeley, 110(1-12), 24.

Saito, T., & Rehmsmeier, M. (2015). The precision-recall plot is more informative than the ROC plot when evaluating binary classifiers on imbalanced datasets. PloS one, 10(3), e0118432.

5. Comparison with existing tools
The manuscript mentions that no tool has been designed for this specific purpose, but this may overlook some existing general-purpose tools or scripts. Many pipelines (such as certain plugins in QIIME 2, USEARCH, etc.) possess functionalities to identify primers or evaluate amplicon regions. The authors should discuss how their tool compares to these existing workflows.

R/ Thank you for the comments. We have added the following text in lines 364-379:

Lines 364-379: "Additionally, some QIIME 2 plugins provide functionality that overlaps with certain steps of the HVRLocator workflow. For example, q2-cutadapt [38] uses cutadapt to remove adapter sequences, primers, and other unwanted sequences from high-throughput sequencing reads, thereby ensuring clean data for downstream analysis, but it requires prior knowledge of the exact primer or unwanted sequences used in the samples in order to accurately detect and remove them. Similarly, the quality-control filter-reads plugin [38] filters demultiplexed single- or paired-end sequences based on their alignment to a reference database using Bowtie 2 [39] and SAMtools [40] to remove contaminants (e.g., human DNA) or to retain only sequences that align to a specified reference, but also relies on alignment to external reference sequences and does not report hypervariable regions or primer information. USEARCH provides functions related to primer matching (e.g., search_oligodb, search_pcr, and search_pcr2) [41]. However, as with QIIME 2, users must supply the primer sequences in advance to perform database matching. In contrast, HVRLocator eliminates the need for prior knowledge of the exact primer sequences by automatically inferring this information."

Minor Comments
1. Confusion regarding processing speed metrics
The abstract mentions a processing speed of "0.147 samples per minute", but later the text mentions "6.5 samples per minute" and "one sample every 0.147 minutes". There is confusion regarding units and values in these three descriptions (is it samples per minute or minutes per sample?). Please unify and correct these data to ensure consistency.

R/ Thank you. The reviewer is correct. There is no confusion regarding the units or values; however, we agree that the message was not clear or fully consistent. The information was derived from Table S5.

The value reported in the phrase "...at an average rate of 6.5 samples per minute" corresponds to the mean of the values in the last column ("Samples processed per minute").

The value reported in the statement “one sample every 0.147 minutes” corresponds to the mean of the column labeled “Time to process 1 sample.”

Table S5: Number of samples per dataset and run time (in minutes) using 8 GB of RAM and 4 CPU cores. We selected samples from the Earth Microbiome Project (Dataset 1), MiCoDa V1 (Dataset 2), and Datathon activities (Dataset 4). All samples were downloaded from the NCBI.

Dataset	Threshold	Duration (minutes)	Total Samples (Run Accession Numbers)	Samples processed successfully	Samples Not Processed (Warnings)	Time to process 1 sample	Samples processed per minute
1	0.5	2078.26	17537	16059	1478	0.1185	7.7271
2	0.5	2746.85	18426	16771	1655	0.1491	6.1055
4	0.5	922.38	5308	5163	145	0.1738	5.5975
1	0.6	2106.22	17537	16059	1478	0.1201	7.6245
2	0.6	2746.85	18426	16771	1655	0.1491	6.1055
4	0.6	904.98	5308	5163	145	0.1705	5.7051
1	0.7	2114.30	17537	16059	1478	0.1206	7.5954
2	0.7	2759.60	18426	16771	1655	0.1498	6.0773
4	0.7	917.05	5308	5163	145	0.1728	5.6300

For greater clarity, we have revised the text as follows:

1.Abstract: “HVRLocator can process archived 16S rRNA sequences from NCBI SRA at 6.5 samples per minute.”
We modified the sentence as follows: “HVRLocator can process archived 16S rRNA sequences from NCBI SRA at an average rate of 6.5 samples per minute.”

3.Case Study: “HVRLocator processed approximately one sample every 0.147 minutes, using 8 GB of RAM and 4 CPU cores.”
We modified the sentence as follows: “HVRLocator processed samples at an average rate of 6.5 samples per minute, using 8 GB of RAM and 4 CPU cores.”

2. Usage of fastq-dump
The use of fastq-dump is mentioned. The SRA Toolkit's fastq-dump is relatively slow and has largely been superseded by fasterq-dump for efficiency. Why did the authors not use the more efficient fasterq-dump?

R/ We thank the reviewer for pointing this out. We agree that fasterq-dump is generally more efficient and represents the recommended approach for large-scale data retrieval. In the current implementation, however, we download only the first 10,000 reads from each sample for primer inference. Under these conditions, the overall runtime is dominated by the time required to establish connections to the server rather than by the data transfer itself, and the use of a multi-threaded tool such as fasterq-dump does not result in a substantial performance improvement. Nevertheless, we acknowledge the reviewer’s suggestion and will consider adopting fasterq-dump in future versions of the tool to improve efficiency.

3. Definition of "Standardized metadata"

The term "standardized metadata" is used frequently. Please explicitly define what constitutes "standard" metadata in the context of this tool within the text.

R/ Thank you for the observation. We have added the following text in lines 95-98:

Lines 95-98: “Standardized metadata is structured information that follows agreed-upon standards, ensuring consistency and comparability across studies and databases through defined fields, controlled vocabularies or ontologies, and standardized formats.”

4. Robustness and error handling
The results section mentions that some samples failed due to "NCBI portal-related issues". Does this imply the tool lacks breakpoint resumption or retry mechanisms?

R/ We apologize for the confusion. The tool includes a retry mechanism to handle temporary NCBI portal issues. The reported failures were due to persistent external server problems rather than a lack of retry mechanisms in the tool. We have added this clarification to the results section:

Lines 239-241: “Although HVRLocator incorporates an automatic retry mechanism to mitigate temporary NCBI portal interruptions, a small number of samples failed due to persistent external server-side issues beyond the control of the tool.”

Given that network fluctuations are common during large-scale downloads, how is the tool's robustness demonstrated?

R/ For any error in the download of a dataset using “fastq-dump”, we have implemented a retry function that tries to retrieve the data three times before moving to the next entry. This way we assure to bypass any temporary issues at the servers level.
Lastly, at the end of a whole run, the user can select the IDs of the failed entries and submit again to a new run. We opted to not automatically try the “failed” downloads again at the end and let the user decide what to do with those. From our experience, these runs are usually simply not available in the Sequence Read Archive (SRA) (Jurburg et al, 2020).

Jurburg, S.D., Konzack, M., Eisenhauer, N. and Heintz-Buschart, A. (2020) The archives are half-empty: an assessment of the availability of microbial community sequencing data. Communications Biology; 3 (474)

5. Output confidence intervals
The output file contains "TRUE/FALSE" and a probability score. For samples where the probability score is at a critical threshold (e.g., around 0.5), does the tool provide an "uncertain" tag, or does it force a classification? It is suggested to add an indicator for ambiguous ranges.

R/ The cutoff value used (0.5) represents a validated and balanced threshold that is not overly conservative, thereby avoiding an excessive number of false positives. However, we provide users with the underlying probability scores values, which can be manually adjusted directly within

the results table to apply custom filtering according to their preferences. This allows users to disregard the binary TRUE/FALSE classification and instead filter results based solely on the probability score.
HVRLocator uses the 0.5 threshold by default because it was validated using the Random Forest model. Any alternative threshold selected by the user is applied at their own judgment and responsibility.

We add the following text in the main manuscript in order to clarify this:

Line 196-200:"Additionally, HVRLocator provides the underlying probability scores for primer presence, allowing users to manually adjust filtering based on their own criteria instead of relying solely on the default TRUE/FALSE classification. The default 0.5 threshold was validated using the Random Forest model, and any alternative threshold chosen by the user is applied at their own discretion."

--
6. Please also take a moment to check our website at https://www.editorialmanager.com/giga/l.asp?i=238196&l=G6VFB430 for any additional comments that were saved as attachments. Please note that as GigaScience has a policy of open peer review, you will be able to see the names of the reviewers.

R/ Done.

In compliance with data protection regulations, you may request that we remove your personal registration details at any time. (Use the following URL: https://www.editorialmanager.com/giga/login.asp?a=r). Please contact the publication office if you have any questions.
