# Supplementary material for: HVRLocator: a computationally efficient tool for identifying hypervariable regions in large 16S rRNA datasets
Source: Gigascience. 2026 Apr 8;15:giag040. doi: 10.1093/gigascience/giag040 (PMC13188219; doi:10.1093/gigascience/giag040)
Supplement: giag040_GIGA-D-25-00344_Revision_2 [file giag040_giga-d-25-00344_revision_2.pdf]

GigaScience

HVRLocator: A Computationally Efficient Tool for Identifying Hypervariable Regions in Large 16S rRNA Datasets

--Manuscript Draft--

**Manuscript Number:**	GIGA-D-25-00344R2
**Full Title:**	HVRLocator: A Computationally Efficient Tool for Identifying Hypervariable Regions in Large 16S rRNA Datasets
**Article Type:**	Technical Note
**Funding Information:**	German Centre for Integrative Biodiversity Research (iDiv) (sIBTEDS project (Illuminating Blindspots Through Equitable Data Reuse practices in the Global South)) — Dr Stephanie D. Jurburg
**Abstract:**	Background: Metabarcoding of the 16S rRNA gene is widely used to assess microbial diversity due to its cost-effectiveness and efficiency. However, publicly available 16S rRNA metabarcoding datasets often lack standardized metadata, particularly information on the sequenced hypervariable regions or primers used, which are critical to their accurate reuse. To address this, we present HVRLocator, a computational tool that (1) identifies the start and end positions of 16S rRNA amplicons, (2) determines their corresponding hypervariable regions, and (3) detects the presence of primer sequences. This tool was validated on four datasets comprising 41,513 samples generated with different primers and sequencing platforms.<br>Results: HVRLocator can process archived 16S rRNA sequences from NCBI SRA at an average rate of 6.5 samples per minute. Validation showed it reliably detects amplicon start and end positions across datasets sequenced with different primers and platforms, achieving 100% accuracy within single-platform studies and correctly revealing length heterogeneity across platforms. It also flagged misannotated metadata and problematic sequences, underscoring its value as a sequence data curation tool. Finally, HVRLocator can select comparable sequences to build large 16S rRNA amplicon databases spanning the same hypervariable region, facilitating cross-study comparisons.<br>Conclusion: HVRLocator overcomes unreliable metadata by accurately identifying 16S rRNA amplicon start and end positions, determining hypervariable regions, and detecting primer sequences, enabling accurate curation and large-scale processing of 16S rRNA data for reliable and reproducible microbial studies, syntheses, and meta-analyses.
**Corresponding Author:**	Clara Maria Arboleda-Baena, Ph.D.<br>iDiv: German Centre for Integrative Biodiversity Research (iDiv) Halle-Jena-Leipzig<br>Leipzig, GERMANY
**Corresponding Author Secondary Information:**	
**Corresponding Author's Institution:**	iDiv: German Centre for Integrative Biodiversity Research (iDiv) Halle-Jena-Leipzig
**Corresponding Author's Secondary Institution:**	
**First Author:**	Clara Arboleda-Baena, Ph.D.
**First Author Secondary Information:**	
**Order of Authors:**	Clara Arboleda-Baena, Ph.D.
	Felipe Borim Correa
	Joao Pedro Saraiva
	Santiago Castillo-Rivadeneira
	Jonas Coelho Kasmanas
	Antonis Chatzinotas

Powered by Editorial Manager® and ProduXion Manager® from Aries Systems Corporation

	Stephanie D. Jurburg
**Order of Authors Secondary Information:**	
**Response to Reviewers:**	GIGA-D-25-00344R1<br>HVRLocator: A Computationally Efficient Tool for Identifying Hypervariable Regions in Large 16S rRNA Datasets<br>Clara Arboleda-Baena; Felipe Borim Correa; Joao Pedro Saraiva; Santiago Castillo-Rivadeneira; Jonas Coelho Kasmanas; Antonis Chatzinotas; Stephanie D. Jurburg<br>GigaScience<br><br>Dear Dr Arboleda-Baena,<br><br>Your manuscript "HVRLocator: A Computationally Efficient Tool for Identifying Hypervariable Regions in Large 16S rRNA Datasets" (GIGA-D-25-00344R1) has been assessed by our reviewers. Based on these reports, and my own assessment as Editor, I am pleased to inform you that it is potentially acceptable for publication in GigaScience, once you have carried out some essential revisions suggested by our reviewers.<br><br>Their reports, together with any other comments, are below. Please also take a moment to check our website at https://www.editorialmanager.com/giga/ for any additional comments that were saved as attachments.<br><br>In addition, please register any new software application in the bio.tools and SciCrunch.org databases to receive RRID (Research Resource Identification Initiative ID) and biotoolsID identifiers, and include these in your manuscript. Computational workflows should be registered in workflowhub.eu and the DOIs cited in the relevant places in the manuscript. These will facilitate tracking, reproducibility and re-use of your tool.<br><br>Once you have made the necessary corrections, please submit a revised manuscript online at:<br><br>https://www.editorialmanager.com/giga/<br><br>If you have forgotten your username or password please use the "Send Login Details" link to get your login information. For security reasons, your password will be reset.<br><br>Please include a point-by-point within the 'Response to Reviewers' box in the submission system. Please ensure you describe additional experiments that were carried out and include a detailed rebuttal of any criticisms or requested revisions that you disagreed with. Please also ensure that your revised manuscript conforms to the journal style, which can be found in the Instructions for Authors on the journal homepage. If the data and code has been modified in the revision process please be sure to update the public versions of this too.<br><br>The due date for submitting the revised version of your article is 27 Mar 2026.<br><br>We look forward to receiving your revised manuscript soon.<br><br>Best wishes,<br><br>Dongni Ma, Ph.D.<br>GigaScience<br>www.gigasciencejournal.com<br><br>R/ Thank you very much, attached you can find the Response to Reviewers.<br><br>Reviewer reports:<br>Reviewer #1: I believe that the authors have addressed the concerns that I raised - in particular the question about whether using a single E. coli sequence would detect divergent bacteria which was raised by the other referee too. If I have understood their methodology correctly they are characterising a sample at a time and while there may be some individual sequences that do not "match" [sensu lato] E. coli there will almost

Powered by Editorial Manager® and ProduXion Manager® from Aries Systems Corporation

always be sequences that are sufficiently similar to E. coli for their method to work. In their response to referees, the authors say:

"Even for bacterial taxa that are highly divergent from E. coli, the 16S rRNA gene remains highly conserved across Bacteria, ensuring sufficient sequence similarity for reliable alignment to the appropriate reference region. Although 16S rRNA sequences vary in length among taxa, our validation showed 99% agreement between the predicted hypervariable regions and those reported in the original publications (metadata or NCBI records). Together, the conserved structure of the 16S rRNA gene and the strong empirical validation support the conclusion that the current alignment strategy provides reliable estimates of start and end positions for hypervariable regions in Bacteria and adequately addresses the objectives of this study."

I would reccommend that the sense of this response is included more explicitly into the final manuscript, perhaps with a cross reference to supplementary material, and that the authors check through their other responses to the referees to make sure that the arguments that they provided there are included within the main part of the manuscript.

R/ We reviewed the responses in the previous file “Response to Reviewers_GIGA-D-25-00344” and found that most of the reviewers’ concerns corresponded to comments that had already been addressed in the main manuscript. However, we have taken the reviewer’s final suggestion into account and added the following text to the main manuscript:

Lines 275-283: “Importantly, validation highlighted HVRLocator tool’s ability to identify problematic sequences. For example, we observed 932 samples with abnormally long average sequence lengths (i.e., >600 bp) that exceeded the expected output lengths with Illumina platforms. Upon manually reviewing these sequences, we found that either the sequencing platform was incorrectly annotated in the metadata (NCBI or the associated publication), or the sequences did not correspond to the 16S rRNA gene but rather to the Internal Transcribed Spacer (ITS) region or the nifH gene. This highlights the use of HVRLocator as a curation tool for large datasets, where human errors in annotation can significantly impact downstream analysis. For further details, see Supplementary Table S6.”

We also added a new supplementary table (Supplementary Table S6), which contains the following:

Even for bacterial taxa that are highly divergent from E. coli, the 16S rRNA gene remains highly conserved across Bacteria, ensuring sufficient sequence similarity for reliable alignment to the appropriate reference region. Although 16S rRNA sequences vary in length among taxa, our validation showed 99% agreement between the predicted hypervariable regions and those reported in the original publications (metadata or NCBI records). Together, the conserved structure of the 16S rRNA gene and the strong empirical validation support the conclusion that the current alignment strategy provides reliable estimates of start and end positions for hypervariable regions in Bacteria and adequately addresses the objectives of this study.

Regarding Archaea and Eukaryotes, we performed several tests and identified potential warning signs that may alert users to review the metadata of sequences exhibiting the following patterns:

1. Putative ITS region: Sequences showing coverage across more than four or five hypervariable (HV) regions. In such cases, users should verify the following information in the metadata: the sequencing platform. If the platform used for these sequences is not PacBio or Nanopore, the primers may be targeting regions that are not specific to bacteria. If the mentioned platforms were used, users should also verify that the primers are not targeting Archaea or Eukaryotes.

The following table presents examples of ITS regions analyzed using HVRLocator: Sample_IDPrimer PresenceScore Primer PresenceMin Alignment startMax Alignment endAverage Alignment startAverage Alignment endMedian Alignment startMedian Alignment endPredicted HV region startPredicted HV region endCoverage based HV region startCoverage based HV region endCoverage HV region startCoverage HV

Powered by Editorial Manager® and ProduXion Manager® from Aries Systems Corporation

	region endWarnings<br>SRR24285816FALSE0.17015403741068399 1148V3V7V4V70.450.72V3 below threshold of 0.5<br>SRR24285817FALSE0.17015403989923991110V3V7V4V60.450.39V3 and V7 below threshold of 0.5<br>SRR24285818FALSE0.17015404001018399 1110V3V7V4V60.450.39V3 and V7 below threshold of 0.5<br>SRR24285819FALSE0.17015404029943991110V3V7V4V60.450.39V3 and V7 below threshold of 0.5<br>SRR24285820FALSE0.17015404461017399 1110V3V7V4V60.450.39V3 and V7 below threshold of 0.5<br>SRR24285821FALSE0.16015404311000399 1110V3V7V4V60.450.39V3 and V7 below threshold of 0.5<br>SRR24285822FALSE0.17015403749533991110V3V7V4V60.450.39V3 and V7 below threshold of 0.5<br>SRR24285823FALSE0.17015403929613991010V3V6V4V60.450.69V3 below threshold of 0.5<br>SRR24285824FALSE0.170154039296839 9953V3V6V4V50.450.38V3 and V6 below threshold of 0.5<br>SRR24285825FALSE0.17015404019633999 53V3V6V4V50.450.38V3 and V6 below threshold of 0.5<br>SRR25665816FALSE0.170154035787218 6826V2V5V2V40.560.2V5 below threshold of 0.5<br>SRR25665819FALSE0.20154069011729031450V6V9V6V80.910.34V9 below threshold of 0.5<br>SRR25665821FALSE0.220154042595618 6826V2V5V2V40.560.2V5 below threshold of 0.5<br>SRR25665824FALSE0.20154062712106851210V4V8V5V70.380.16V4 and V8 below threshold of 0.5<br>SRR25665826FALSE0.22015406021118574 1048V4V6V4V60.720.91NA<br>SRR25665828FALSE0.20154030311641771096V2V7V2V60.610.27V7 below threshold of 0.5<br>SRR25665830FALSE0.17015406001149529 1048V4V6V4V60.850.91NA<br>SRR25665832FALSE0.210154044997018 6826V2V5V2V40.560.2V5 below threshold of 0.5<br>ERR5924913FALSE0.17015404821055548 1111V4V7V4V60.80.4V7 below threshold of 0.5<br>ERR5924916FALSE0.17015404631076595 1177V4V7V4V70.650.97NA<br><br>2. Putative Archaea or 18S hypervariable region: As clarified in the manuscript, users must ensure that the target domain is Bacteria. Due to similarities in gene length and conserved regions, sequences targeting archaeal 16S rRNA genes or eukaryotic 18S rRNA genes may still produce a prediction when analyzed with HVRLocator. However, the tool is specifically designed and validated for bacterial 16S rRNA sequences. Therefore, verification of the sample metadata is essential to confirm that the predicted hypervariable region corresponds to bacterial sequences.<br><br>Reviewer #2: The authors have made proactive improvements in both the functional flexibility of the program (supporting custom reference sequences) and the expansion of the taxonomic scope (mentioning future support for ITS, etc.); therefore, this manuscript would be of significant value for publication as a Technical Note in GigaScience.<br><br>R/ We thank the reviewer for their positive feedback.<br><br>--<br>Please also take a moment to check our website at https://www.editorialmanager.com/giga/l.asp?i=242341&l=0KJITSI3 for any additional comments that were saved as attachments. Please note that as GigaScience has a policy of open peer review, you will be able to see the names of the reviewers.<br><br>R/ Done, thank you.
**Additional Information:**	

Powered by Editorial Manager® and ProduXion Manager® from Aries Systems Corporation

Question	Response
Are you submitting this manuscript to a special series or article collection?	No
**Experimental design and statistics**<br><br>Full details of the experimental design and statistical methods used should be given in the Methods section, as detailed in our Minimum Standards Reporting Checklist. Information essential to interpreting the data presented should be made available in the figure legends.<br><br>Have you included all the information requested in your manuscript?	Yes
**Resources**<br><br>A description of all resources used, including antibodies, cell lines, animals and software tools, with enough information to allow them to be uniquely identified, should be included in the Methods section. Authors are strongly encouraged to cite Research Resource Identifiers (RRIDs) for antibodies, model organisms and tools, where possible.<br><br>Have you included the information requested as detailed in our Minimum Standards Reporting Checklist?	Yes
**Availability of data and materials**<br><br>All datasets and code on which the conclusions of the paper rely must be either included in your submission or deposited in publicly available repositories (where available and ethically appropriate), referencing such data using a unique identifier in the references and in the "Availability of Data and Materials" section of your manuscript.	Yes

Powered by Editorial Manager® and ProduXion Manager® from Aries Systems Corporation

Have you have met the above requirement as detailed in our Minimum Standards Reporting Checklist?	
GigaScience has policies and guidelines in place for the use of generative AI-writing tools such as ChatGPT. If you have used such writing tools to assist with writing the manuscript this must be declared and cited in the text. Authors should not list AI-writing tools and other AI-assisted technologies as an author or co-author and should acknowledge that they are fully responsible for text generated or refined by AI-writing tools.<p><br><br>A summary of use (particularly in the introduction or among methods) needs to be included at the end of the paper, and the outputs should also be included as a supplementary file hosted in GigaDB or other open repositories. Please read our guidelines for more information. <p><br><br>By submitting to GigaScience, you are aware of the journal's AI-writing tools policy, and if you have declared use of such tools below, you have acknowledged this where appropriate in your manuscript and have made a summary of use and outputs available. <p><br><b>AI-assisted writing tools have been used in the preparation of this manuscript?	No

Powered by Editorial Manager® and ProduXion Manager® from Aries Systems Corporation

Manuscript
Click here to access/download;Manuscript;GIGA-D-25-00344R1_HVRLocator_Manuscript_202604.docx

HVRLocator: A Computationally Efficient Tool for Identifying Hypervariable Regions in Large 16S rRNA Datasets

Clara Arboleda-Baena[1,2‡*], Felipe Borim Correa[2‡], Joao Pedro Saraiva[2], Santiago Castillo-Rivadeneira[1], Jonas Coelho Kasmanas[2], Antonis Chatzinotas[1,2,3], Stephanie D. Jurburg[1,2*]

[1] German Centre for Integrative Biodiversity Research (iDiv) Halle-Jena-Leipzig, 04103 Leipzig, Germany
[2] Department of Applied Microbial Ecology, Helmholtz Centre for Environmental Research - UFZ, 04318 Leipzig, Germany
[3] Institute of Biology, Leipzig University, 04103 Leipzig, Germany

‡*CAB and FBC contributed equally to this work*
*Corresponding authors: claraarboledab@gmail.com and s.d.jurburg@gmail.com

ORCIDs
Clara Maria Arboleda Baena https://orcid.org/0000-0002-5185-8790
Felipe Borim Correa : https://orcid.org/0000-0003-0031-1565
Joao Pedro Saraiva : https://orcid.org/0000-0001-8251-1960
Santiago Castillo Rivadeneira: https://orcid.org/0009-0006-0058-8547
Jonas Coelho Kasmanas : https://orcid.org/0000-0001-6513-5350
Antonis Chatzinotas : https://orcid.org/0000-0002-0387-9802
Stephanie D. Jurburg : https://orcid.org/0000-0002-7701-6030

Abstract

Background: Metabarcoding of the 16S rRNA gene is widely used to assess microbial diversity due to its cost-effectiveness and efficiency. However, publicly available 16S rRNA metabarcoding datasets often lack standardized metadata, particularly information on the sequenced hypervariable regions or primers used, which are critical to their accurate reuse. To address this, we present HVRLocator, a computational tool that (1)

identifies the start and end positions of 16S rRNA amplicons, (2) determines their corresponding hypervariable regions, and (3) detects the presence of primer sequences. This tool was validated on four datasets comprising 41,513 samples generated with different primers and sequencing platforms.

Results: HVRLocator can process archived 16S rRNA sequences from NCBI SRA at an average rate of 6.5 samples per minute. Validation showed it reliably detects amplicon start and end positions across datasets sequenced with different primers and platforms, achieving 100% accuracy within single-platform studies and correctly revealing length heterogeneity across platforms. It also flagged misannotated metadata and problematic sequences, underscoring its value as a sequence data curation tool. Finally, HVRLocator can select comparable sequences to build large 16S rRNA amplicon databases spanning the same hypervariable region, facilitating cross-study comparisons.

Conclusion: HVRLocator overcomes unreliable metadata by accurately identifying 16S rRNA amplicon start and end positions, determining hypervariable regions, and detecting primer sequences, enabling accurate curation and large-scale processing of 16S rRNA data for reliable and reproducible microbial studies, syntheses, and meta-analyses.

Keywords: Big data, 16S rRNA gene, metabarcoding, high throughput sequencing, metadata, microbial ecology.

Background

While the existence of bacteria has been known for over three centuries, the ability to study all individuals in a bacterial community is relatively novel. By extracting and sequencing nucleic acids from hosts or environmental samples, it is now possible to characterize the taxonomic diversity of a bacterial community without the need to cultivate its members. Metabarcoding, which focuses sequencing efforts on a segment of a universal marker gene, or amplicon (typically the 16S rRNA gene for prokaryotes), has emerged as a dominant technique due to its technical ease and low cost. To date, 16S rRNA gene metabarcoding has uncovered the extreme diversity and ubiquity of microbes [1], while revealing avenues for improving human health, agricultural productivity, and sustainability [2]. At the same time, metabarcoding datasets archived in public

repositories have grown exponentially [3]. These data are uniform in format, are routinely archived with technical and experimental metadata, and are a rich and growing resource for synthetic and large-scale research. However, metabarcoding data are archived in their raw format, and the metadata needed for bioinformatics processing is often unavailable or not curated, creating barriers to data reuse [3,4].

Technical metadata are central to sequence data harmonization and reuse as they provide context for the data [5] and directly inform bioinformatics processing. Technical choices preceding sequencing, most notably the DNA extraction kit [6], sequencing platform [7], and target amplicon [8,9] have been shown to affect microbial diversity assessments [10]; however the integration of these data *in light* of Big Data processing and synthesis has received less attention. For example, Abdill and colleagues [11] restricted their synthesis to sequences obtained from Illumina technologies to use a unified processing pipeline, but did not enforce consistent amplicon sequence lengths, even though detected bacterial diversity increases linearly with amplicon length [12].

The 16S rRNA gene contains both highly conserved regions that are essential for primer design, and hypervariable regions that allow for the phylogenetic identification of microorganisms [13]. Full-length 16S rRNA gene sequences (~1500 bp) comprise nine hypervariable regions interspersed with nine highly conserved regions [14,15]. Identifying which 16S rRNA gene segment was targeted for amplification is crucial to fully leverage sequence length and coverage, which ultimately determines the efficiency and accuracy of downstream processing and taxonomic classification pipelines. In the case of paired-end reads (e.g., Illumina technologies), the length of the target region further informs the minimum read length needed to achieve a successful merger of the pair [16].

At the same time, the choice of the sequenced region can significantly affect the relative abundances of detected organisms. For example, Wasimuddin et al. [8] found that compared to three other primer sets targeting different regions, the primer pair targeting the V4 hypervariable region of the 16S rRNA gene produced the highest estimates of Amplicon Sequence Variants (ASVs) richness and diversity across various sample types (including soil, maize roots, cattle rumen, and cattle and human feces). However, different

primers targeting the same variable region can still generate different numbers of ASVs [17].

Crucially, as novel sequencing technologies and platforms have emerged, the length of the target amplicons has also varied extensively, ranging from 150 base pair segments (e.g., Illumina HiSeq single-end sequencing) to whole genes (e.g., nanopore sequencing), resulting in massive heterogeneity in the length and location of the sequenced regions. Variation in amplicon length complicates the reuse of 16S rRNA metabarcoding data, since longer sequences tend to detect greater bacterial diversity and taxonomic resolution [12].

Considering technical information about the sequenced region during downstream bioinformatics processing is essential to metabarcoding data reuse, but this information is often missing from the metadata, vague, or incorrect [18]. Standardized metadata is structured information that follows agreed-upon standards, ensuring consistency and comparability across studies and databases through defined fields, controlled vocabularies or ontologies, and standardized formats. The lack of standardized metadata significantly slows down the compilation of large datasets, making it difficult to reprocess metabarcoding sequences collectively. Here, we present HVRLocator, a computational tool designed to efficiently identify hypervariable regions of the 16S rRNA gene sequenced for a set of metabarcoding samples. By optimizing computational resources, our approach enables rapid and accurate screening of large datasets, facilitating more comprehensive and scalable microbial diversity analyses.

Materials and methods

Design

HVRLocator identifies which segment of the 16S rRNA gene was sequenced for a given set of metabarcoding samples. The full pipeline uses Python programming language, and users can access it either via a singularity container or by installing it locally on their computer. For further details on installation and usage please see: HVRLocator GitHub web page [19].

As input, HVRLocator accepts text file (.txt) lists of accession numbers compliant with International Nucleotide Sequence Collaboration (INSDC) databases, including the European Nucleotide Archive (ENA) at the European Bioinformatics Institute, the Sequence Read Archive (SRA) at the National Center for Biotechnology Information, and the DNA Databank of Japan (DDBJ) Sequence Read Archives at the National Institute of Genetics [20], bypassing the need to download the data a priori. HVRLocator also accepts resolved amplicons (e.g., Amplicon Sequence Variants (ASV)) or raw sequencing data in. FASTA or. FASTQ file formats of the 16S rRNA gene.

For INSDC data, the tool employs a multi-step process. First, it retrieves 1000 reads using fastq-dump from the SRA Toolkit [21] and performs quality control and trimming with fastp [22]. Then, for each sample, the HVRLocator identifies the data type (single- or paired-end DNA). For paired-end reads, it merges the processed reads using VSEARCH [23], while for single-end reads, it directly converts the trimmed FASTQ to FASTA format. The tool then aligns the processed sequences to a reference 16S rRNA gene sequence from an *Escherichia coli* reference genome (J01859.1) using MAFFT [24]. HVRLocator subsequently analyzes the aligned sequences to determine hypervariable regions by identifying the median start and end alignment positions relative to the *E. coli* 16S rRNA gene reference sequence, using established coverage thresholds (default = 0.6) for the conserved and hypervariable regions of the 16S rRNA gene [13,25]. To note, the thresholds can be adjusted by adding the "-t" flag. The output is a tab-separated values (TSV) file containing alignment start and end positions, as well as the boundaries (median and average start and end positions, and minimum start and maximum end positions) of the identified hypervariable regions. HVRLocator is currently limited to bacterial 16S rRNA sequences, as it aligns them to the E. coli 16S rRNA reference.

Finally, a Random Forest (RF) model was designed to predict the presence of a primer in a given SRA sequencing dataset by analyzing the quality score distribution of the initial subset of reads. RF performs well under moderate class imbalances when class-aware evaluation and sampling is applied [26,27]. Additionally, distinct quality score patterns are known to occur in the first few cycles of Illumina sequencing when base diversity is low [28] as is in the case of untrimmed primers at the start of reads. Quality score patterns have also been used to detect sequencing bias and artifacts by tools such as DADA2 [29]

and Mapinsights [30]. Thus, this metric can serve as a proxy for detecting primer presence. To this end, we selected a curated collection of SRA samples with (882 samples) and without primers (8940 samples, **Supplementary Tables S1 and S2**). For each sample, the first 1,000 reads from each sample were extracted using fastq-dump (NCBI SRA Toolkit, 3.2.1) and two quality score segments from positions 1-5 and 6-10 were calculated. Eight statistical features were obtained: count, mean, median, standard deviation, minimum, maximum, an estimate of skewness (approximated by the 25th percentile), and kurtosis (approximated by the 75th percentile), resulting in 16 features per sample. The model was trained using scikit-learn's RandomForestClassifier (v1.2.1) with 100 estimators and a fixed random seed (random_state=42), using an 80/20 stratified train-test split. The Random Forest model yielded a precision of 99.96% for the dataset without primers and 100% for the dataset with primers. Recall of the model using the "no-primer" and "primer" dataset was 100% and 99.55%, respectively. Full details on the model generation including the algorithm, versions and packages are available in the **Supplementary Table S3.**

HVRLocator is available as a singularity container [31], and can be executed on High Performance Computing (HPC) clusters or cloud computing platforms. Further, Singularity enables the seamless execution of containers without requiring root privileges, maintaining security and reproducibility. HVRLocator will be actively maintained with updates for compatibility and user feedback. Issues or feature requests can be sent to the corresponding author. The HVRLocator output is a text file (.txt) containing the following columns:

1.Sample_ID: Identifier of the processed sample (Run Accession number).
2.Primer Presence: Presence or absence of a primer (TRUE/FALSE)
3.Score Primer Presence: The associated probability value ranging from 0 to 1.
4.Min/Max Alignment Start/End: Minimum (0) and maximum (1540) possible alignment positions along the 16S rRNA gene.
5.Average Alignment Start/End: The mean position where reads align to the 16S rRNA gene. The *start* indicates the average starting position, and the *end* indicates the average ending position across all reads in the sample.

6.Median Alignment Start/End: The median position where reads align to the 16S rRNA gene. The start indicates the median starting position, and the end indicates the median ending position across all reads in the sample.

7.Predicted HV region Start/End: Predicted hypervariable (HV) region based on the median alignment start and end positions across all reads, inferred from literature on conserved and hypervariable regions of the 16S rRNA gene (Brosius et al., 1978; Yang et al., 2016).

8.Coverage based HV region Start/End: Predicted hypervariable region based on coverage at the start and end positions across all reads.

9.Coverage HV region Start/End: Coverage values (0-1) for the "*Coverage based HV region*" start or end position across all reads. 0 = no reads cover that position; 1 = all reads cover that position

10.Warnings: Alerts about low coverage regions. See possible errors and troubleshooting.

11-19. Cov_V1 to Cov_V9: Coverage values (0-1) for each HV region.

Importantly, validation showed that the median alignment position is a more reliable indicator of the sequenced region than the average alignment position, as low-quality sequences within a sample can skew the mean and lead to an incorrect identification of the hypervariable region. For this reason, we report both metrics, but recommend prioritizing the median when deciding whether to retain or discard sequences during downstream processing. Additionally, HVRLocator provides the underlying probability scores for primer presence, allowing users to manually adjust filtering based on their own criteria instead of relying solely on the default TRUE/FALSE classification. The default 0.5 threshold was validated using the Random Forest model, and any alternative threshold chosen by the user is applied at their own discretion.

Finally, in addition to identifying the hypervariable region based on the average and median start and end positions of the amplicons within each sample, HVRLocator also reports coverage across all nine hypervariable regions for every individual sample. This allows users to assess the amplicon length, determine its exact start and end positions, and evaluate the coverage of specific hypervariable region(s) within each sample.

Validation

HVRLocator ‘s processing stability was calculated by measuring the sample time for 1, 10, 100, 1000 and 10000 samples using the same cluster resources: 8 GB of RAM and 4 CPU cores to emulate the standard capabilities of a personal computer.

HVRLocator was validated by analyzing four datasets which contained samples sequenced using a) same primer and same sequencing platform, b) different primers and the same platform, c) the same primer and different sequencing platforms, and d) different primers and sequencing platforms. Dataset *a* included 17,537 samples from the Earth Microbiome Project [32], in which all samples were sequenced on the Illumina Miseq platform using the 515F–806R primer set targeting the V4 hypervariable region of the 16S rRNA gene [32]. Dataset *b* included 242 samples from two studies that compared different primers but were sequenced on the same platform [8,33]. Dataset *c* included 18,426 samples from the MiCoDa database Version 1, which were compiled from available literature and data [34]**,** to select samples that were sequenced in the same 515-806 region of the 16S SSU rRNA (Small Subunit Ribosomal RNA) with various sequencing platforms. The primer information for each sample was obtained from the metadata archive in NCBI. For dataset *d,* we selected 5,308 samples compiled during the Datathon project in Latin America [35]**.** These samples employed various primer sets targeting different regions of the 16S rRNA gene and were sequenced on different platforms, and represent a realistic set of samples that might be encountered during data compilation efforts. The run accession numbers used for all datasets are listed in **Supplementary Table S4, 1-4**.

All graphics were carried out in R with RStudio interface [36], and all pipelines are available at GitHub [37].

Results

As the number of samples increased, the running time per sample and computational resource usage remained stable at an average rate of 6.5 samples per minute (**Figure 1 and Supplementary Table S5**), highlighting the tool’s processing stability and scalability for the analysis of large datasets. Failures were primarily due to samples with fewer than

500 reads (72%), alignment errors (14%), missing FASTQ files (13%), and NCBI portal-related issues (1%). (See *Possible Errors and Troubleshooting* at the HVRLocator GitHub web page [19]). Although HVRLocator incorporates an automatic retry mechanism to mitigate temporary NCBI portal interruptions, a small number of samples failed due to persistent external server-side issues beyond the control of the tool.

By analyzing the alignment positions across different 16S rRNA hypervariable regions and sequencing setups (**Figure 2**), we found that HVRLocator accurately predicted the alignment positions compared to what is expected from the literature associated with each database. For example, sequences in dataset *a,* consistently aligned with the V4 hypervariable region of the 16S rRNA gene and had a median sequence start of 532 bp, as expected from the standardized primer set (515F-806R) used in the Earth Microbiome Project [1]. Sequence lengths were highly homogeneous, also likely due to the use of the same primer set and sequencing machinery (**Figure 2a**). For a total of 17,537 samples, 16,059 samples were processed successfully without warnings; common issues included missing FASTQ files, low reads, alignment failures, and NCBI portal-related issues. Out of 16,059 samples processed, 9 did not yield the expected results based on the literature. Dataset *b* [8,33], which employed different primers but the same platform, confirmed that HVRLocator correctly matched the sequences to the corresponding, expected primer regions used during sample sequencing with 100% accuracy (**Figure 2b**). For dataset *c* HVRLocator also accurately indicated more heterogeneous alignment start positions and sequence lengths, consistent with our expectation and the compilation of the MiCoDa database from the literature (**Figure 2c)**. Most sequences covered the V4 region (89%), as indicated by both the median alignment start and the coverage-based HV region start (**Figure 2c and Figure 3**, respectively). As expected, the median alignment end varied across projects and sequencing platforms.

To check the reliability of HVRLocator relative to manual extraction of metadata from the literature, we manually extracted data related to the primers used and the 16S rRNA HV region targets from all samples in dataset *c.* For a total of 18,426 samples, 16,771 samples were processed successfully without warnings; common issues included missing FASTQ files, low reads, alignment failures, and NCBI portal-related issues. Of

the 16,771 samples processed, 1,712 (10%) did not produce results consistent with the literature (e.g. mismatches between the start region alignment and the reported primer, or incorrect HV region alignment compared with the reported HV region), underscoring the value of obtaining metadata from the sequence data directly, rather than from the literature. Finally, for the diverse data set that used both different 16S rRNA regions and sequencing setups (**Figure 2d**), HVRLocator accurately and rapidly assigned the alignment positions.

Importantly, validation highlighted HVRLocator tool's ability to identify problematic sequences. For example, we observed 932 samples with abnormally long average sequence lengths (i.e., >600 bp) that exceeded the expected output lengths with Illumina platforms. Upon manually reviewing these sequences, we found that either the sequencing platform was incorrectly annotated in the metadata (NCBI or the associated publication), or the sequences did not correspond to the 16S rRNA gene but rather to the Internal Transcribed Spacer (ITS) region or the nifH gene. This highlights the use of HVRLocator as a curation tool for large datasets, where human errors in annotation can significantly impact downstream analysis. For further details, see **Supplementary Table S6.**

Case study: 45,882 metabarcoding samples for the compilation of a large 16S rRNA gene database.

We present an example of how to use the tool to select correct and comparable sequences to construct a large bacterial database based on metabarcoding sequences targeting the V4 hypervariable region of the 16S rRNA gene. We included samples sequenced from start position 515 bp of the 16S rRNA gene, which is the same starting position as that used by the Earth Microbiome Project primers (515F–806R) [1],. Our input dataset included 45,882 samples spanning a wide variety of matrices (e.g., soil, host-associated, and water), that were sequenced with different primer sets and sequencing platforms. These data were collected through an extensive literature search prioritizing meta-analyses, large amplicon research consortia, and Datathon activities [35] (**Supplementary Table S7**)**.** Using SRA's sequence-associated metadata, we selected

only metabarcoding-derived sequences (i.e., excluding WGA, WGS, Tn-Seq, miRNA-Seq, POOLCLONE, RNA-Seq, etc.).

HVRLocator processed samples at an average rate of 6.5 samples per minute, using 8 GB of RAM and 4 CPU cores. A total of 42,166 samples were processed successfully, while 3,716 samples failed to be processed and generated warnings, mainly due to samples with fewer than 500 reads (66%), missing FASTQ files (21%), alignment errors (13%), and other issues related to the NCBI portal (1%). (See *Possible Errors and Troubleshooting* at the HVRLocator GitHub web page [19]). HVRLocator identified a diverse range of sequences with varying median hypervariable region start and end positions, coverage, and lengths **(Figure 4a)**. The output indicated that 1,532 samples had a true presence of primers, while 40,634 did not.

The detailed output for the average and median alignment columns, as well as the coverage-based start and end values, is provided in **Supplementary Table S8a–d,f .** Of the successfully processed samples, 85.9% (36,217) began in the V4 region of the 16S rRNA gene (**Figure 4b**), and the next most common starting regions were V3 (9.7%), V2 (2.3%), and V1 (1.5%). The predicted end of the hypervariable region for the majority of samples corresponded to the V4 region (85.3%), followed by V6 (6.5%), V8 (3.4%), V5 (1.6%), and V7 (1.4%). (**Supplementary Table S8e**). We retained the 36,217 samples that had a median starting point in the V4 region.

For all sequences, we cross-checked the reported primer information either from the metadata of the research articles or NCBI records against the region predicted by HVRLocator (**Figure 4d**). This allowed us to quantify the number of samples where the actual sequenced region was incorrectly assigned, despite being labeled as targeting the V4 hypervariable region in the metadata. Among the selected samples, 382 (1%) had incorrect primer annotations, either in the NCBI metadata or in the associated publications. These findings highlight that even when metadata is available, it may be inaccurate or misleading and underscores the importance of HVRLocator, which accurately and efficiently identify the sequenced region.

Discussion

INSDC databases currently host over 32 million next-generation sequencing samples [38], and represent a growing resource for large-scale analyses to address global questions through the synthesis and reuse of sequence data. However, efforts of sequence data archiving are undermined by the lack of available metadata [21], especially, as these metadata are crucial to data processing. High quality data are relatively sparse [3], which makes the process of data identification intensive, inefficient, and error-prone. To facilitate the reuse of bacterial metabarcoding data, we developed HVRLocator, a publicly available tool which efficiently identifies the exact region sequenced by a set of 16S rRNA sequences, and can therefore greatly accelerate the identification of candidate datasets for reanalysis. The extensive validation of HVRLocator also highlights its potential for application towards data reuse.

Given the ubiquity of bacteria and their relevance to their environments, a wide range of disciplines employ 16S rRNA gene metabarcoding sequencing, and contribute data to INSDC archives in the process [38]. Indeed, according to available ENA metadata, metabarcoding datasets still dwarf metagenomic datasets by a factor of ten. Due to the lack of curation of INSDC metadata, information derived from peer-reviewed literature has been proposed as a central source of technical metadata that can enrich existing datasets [39], but the diversity of disciplines which employ metabarcoding also results in different degrees of resolution in the technical metadata provided for the sequence data. These metadata may lack the resolution necessary for an improved bioinformatics process or even introduce errors. Here, HVRLocator serves to bypass the need to return to the original literature to obtain the necessary processing metadata and the higher resolution information, such as the exact start and end sequence positions instead of the general region sequenced, and to correct potential errors that might be present in the literature-derived metadata.

To our knowledge, no tool has been designed with the specific purpose of automated metadata extraction from archived metabarcoding datasets to facilitate bioinformatics processing. Based on complete or near-complete 16S rRNA gene sequences and for a given set of primers, HyperEx (HyperVariable Region Extractor) [40] evaluates the precision and accuracy of different primer pairs in retrieving microbial diversity, and was

developed as a tool for primer selection. Similarly, Qscore [9] evaluates the performance of 16S rRNA amplicons, assessing metrics including amplification rate, multitier taxonomic annotation, sequence type, and length. In contrast, HVRLocator does not rely on simulations or *a priori* primer information, and is designed for its application to existing datasets. HVRLocator operates directly on large, INSDC-archived metabarcoding datasets to identify the start and end positions of sequenced 16S rRNA amplicons, determine their corresponding hypervariable regions, and detect the presence primer sequences, generating the technical metadata that is needed for bioinformatics processing of the raw sequences. Additionally, some QIIME 2 plugins provide functionality that overlaps with certain steps of the HVRLocator workflow. For example, q2-cutadapt [41] uses cutadapt to remove adapter sequences, primers, and other unwanted sequences from high-throughput sequencing reads, thereby ensuring clean data for downstream analysis, but it requires prior knowledge of the exact primer or unwanted sequences used in the samples in order to accurately detect and remove them. Similarly, the quality-control filter-reads plugin [41] filters demultiplexed single- or paired-end sequences based on their alignment to a reference database using Bowtie 2 [42] and SAMtools [43] to remove contaminants (e.g., human DNA) or to retain only sequences that align to a specified reference, but also relies on alignment to external reference sequences and does not report hypervariable regions or primer information. USEARCH provides functions related to primer matching (e.g., search_oligodb, search_pcr, and search_pcr2) [44]. However, as with QIIME 2, users must supply the primer sequences in advance to perform database matching. In contrast, HVRLocator eliminates the need for prior knowledge of the exact primer sequences by automatically inferring this information.

Technical metadata is crucial, as species identification depends heavily on the targeted region and its length [12]. In the case of 16S rRNA gene metabarcoding, information about the gene region sequenced is essential for the bioinformatics processing of the sequence data, and for the statistical analyses (i.e., as a random effect in a hierarchical model) and downstream data interpretation. From an ecological perspective, the ability to consistently target the same genetic region across different studies brings us closer to achieving a macroecological understanding of microbial communities [8,12,45]. In this vein,

HVRLocator can support and accelerate the bioinformatics processing of 16S rRNA metabarcoding data, enhancing comparability and improving short-read training sets for future predictive microbiome studies. Although the present work focuses on bacteria, we acknowledge that other domains of life, including Archaea and Eukaryotes, are relevant for future meta-analyses and synthesis studies. Consequently, future versions of the program aim to incorporate alternative model sequences representing Archaea and Eukaryote.

In the future, HVRLocator may support decision-making in the creation of large databases, improving the robustness and resolution of microbiome studies [13,46,47]. As long-read sequencing technologies gain relevance, HVRLocator could serve as a foundation for developing procedures to integrate multiple sequences with different coverage, ultimately enhancing our ability to capture microbial diversity more comprehensively.

Availability of supporting source code and requirements

Project name: HVRLocator

Project home page: https://github.com/fbcorrea/HVRLocator

Operating system(s): Linux OS

Programming language: Python 3.9

Other requirements: Singularity container platform https://cloud.sylabs.io/library/jsaraiva/repo/hvrlocator

License: MIT license

RRID: SCR_027407

biotools: hvrlocator

Additional files

Supplementary Table S1: Run accession list with primers used for training and testing the Random Forest (RF) model.

Supplementary Table S2: Run accession list without primers used for training and testing the Random Forest (RF) model.

Supplementary Table S3: Random Forest Model for Primer Presence Prediction.

Supplementary Table S4: Datasets (1-4) included in the validation process.

Supplementary Table S5: Number of samples per dataset and run time (in minutes) using 8 GB of RAM and 4 CPU cores.

Supplementary Table S6: Evaluation of tool performance on Archaeal and Eukaryotic sequences.

Supplementary Table S7: Run accession list for case study

Supplementary Table S8: Case study output.

Supplementary Table S9: Number of samples in which primer assignment matched the metadata (Figure S1.b)

Supplementary Figure S1: Variation in gene coverage across sequences and number of samples in which primer assignment matched the metadata.

Data Availability

The public datasets used in this paper can be found in **Supplementary Table S1, S2, S4** and **S7**. Machine learning annotations have been deposited in the DOME registry [48] .

Abbreviations

ASV: Amplicon Sequence Variants; DDBJ: DNA Databank of Japan; DRA: Sequence Read Archives; EBI: European Bioinformatics Institute ; ENA: European Nucleotide Agency; HPC: High Performance Computing; INSDC: International Nucleotide Sequence Database Collaboration; ITS: Internal Transcribed Spacer; NCBI: National Center for Biotechnology Information; NIG: National Institute of Genetics; SRA: Sequence Read Archive; SSU rRNA: Small Subunit Ribosomal RNA; TSV: Tab-separated values; UFZ: Helmholtz Center for Environmental Research.

Acknowledgments

The results were computed at the High-Performance Computing (HPC) Cluster EVE, a joint effort of both the Helmholtz Centre for Environmental Research - UFZ and the German Centre for Integrative Biodiversity Research (iDiv) Halle-Jena-Leipzig. We would like to thank the administration and support staff of EVE who keep the system running and support us with our scientific computing needs: Toni Harzendorf, Mark Fliak and Conrad Ostertag from UFZ, and Christian Krause from iDiv. Also, we would like to thank Marten Winter at the Synthesis Centre for Biodiversity Sciences (sDiv) at the German Centre for Integrative Biodiversity Research (iDiv).

Author Contributions

Conceptualization: SJ, FBC, CAB; funding acquisition: SJ; methodology: SJ, FBC, CAB, JPS, SC; software: FBC, JPS, JCK; writing—original draft: SJ, FBC, CAB, JPS; writing—review and editing: all authors. All authors read and approved the final manuscript.

Funding

This work was supported by the Microbiome Machine for Synthesis: Domain-Specific Application of FAIR Data Assessments for Automated Metadata project (funding code ZT-I-PF-3-100) from the Helmholtz Metadata Collaboration (HMC). Additional support was provided by the sIBTEDS project (Illuminating Blindspots Through Equitable Data Reuse Practices in the Global South), a project by sDiv, the synthesis centre of the German Centre for Integrative Biodiversity Research (iDiv).

Competing Interests

The authors declare that they have no competing interests

References

1. Thompson LR, Sanders JG, McDonald D, Amir A, Ladau J, Locey KJ, et al.. A communal catalogue reveals Earth's multiscale microbial diversity. *Nature*. 2017; doi: 10.1038/nature24621.

2. Jurburg SD, Eisenhauer N, Buscot F, Chatzinotas A, Chaudhari NM, Heintz-Buschart A, et al.. Potential of microbiome-based solutions for agrifood systems. *Nat Food*. 2022; doi: 10.1038/s43016-022-00576-x.

3. Jurburg SD, Konzack M, Eisenhauer N, Heintz-Buschart A. The archives are half-empty: an assessment of the availability of microbial community sequencing data. *Commun Biol*. 2020; doi: 10.1038/s42003-020-01204-9.

4. Hassenrück C, Poprick T, Helfer V, Molari M, Meyer R, Kostadinov I. FAIR enough? A perspective on the status of nucleotide sequence data and metadata on public archives. *bioRxiv*. Cold Spring Harbor Laboratory; 2021; doi: 10.1101/2021.09.23.461561.

5. Leigh DM, Vandergast AG, Hunter ME, Crandall ED, Funk WC, Garroway CJ, et al.. Best practices for genetic and genomic data archiving. *Nat Ecol Evol*. Springer Science and Business Media LLC; 2024; doi: 10.1038/s41559-024-02423-7.

6. Tremblay J, Singh K, Fern A, Kirton ES, He S, Woyke T, et al.. Primer and platform effects on 16S rRNA tag sequencing. *Front Microbiol*. Frontiers Media SA; 2015; doi: 10.3389/fmicb.2015.00771.

7. Whon TW, Chung W-H, Lim MY, Song E-J, Kim PS, Hyun D-W, et al.. The effects of sequencing platforms on phylogenetic resolution in 16 S rRNA gene profiling of human feces. *Sci Data*. Springer Science and Business Media LLC; 2018; doi: 10.1038/sdata.2018.68.

8. Wasimuddin, Schlaeppi K, Ronchi F, Leib SL, Erb M, Ramette A. Evaluation of primer pairs for microbiome profiling from soils to humans within the One Health framework. *Molecular Ecology Resources*. 2020; doi: 10.1111/1755-0998.13215.

9. Zhang W, Fan X, Shi H, Li J, Zhang M, Zhao J, et al.. Comprehensive Assessment of 16S rRNA Gene Amplicon Sequencing for Microbiome Profiling across Multiple Habitats. Li D, editor. *Microbiol Spectr*. 2023; doi: 10.1128/spectrum.00563-23.

10. Brooks JP, Edwards DJ, Harwich MD, Rivera MC, Fettweis JM, Serrano MG, et al.. The truth about metagenomics: quantifying and counteracting bias in 16S rRNA studies. *BMC Microbiol*. Springer Science and Business Media LLC; 2015; doi: 10.1186/s12866-015-0351-6.

11. Abdill RJ, Graham SP, Rubinetti V, Ahmadian M, Hicks P, Chetty A, et al.. Integration of 168,000 samples reveals global patterns of the human gut microbiome. *Cell*. Elsevier BV; 2025; doi: 10.1016/j.cell.2024.12.017.

12. Jurburg SD. Short Read Lengths Recover Ecological Patterns in 16S rRNA Gene Amplicon Data. *Molecular Ecology Resources*. 2025; doi: 10.1111/1755-0998.14102.

13. Yang B, Wang Y, Qian P-Y. Sensitivity and correlation of hypervariable regions in 16S rRNA genes in phylogenetic analysis. *BMC Bioinformatics*. 2016; doi: 10.1186/s12859-016-0992-y.

14. Baker GC, Smith JJ, Cowan DA. Review and re-analysis of domain-specific 16S primers. *Journal of Microbiological Methods*. 2003; doi: 10.1016/j.mimet.2003.08.009.

15. Wang Y, Qian P-Y. Conservative Fragments in Bacterial 16S rRNA Genes and Primer Design for 16S Ribosomal DNA Amplicons in Metagenomic Studies. Field D, editor. *PLoS ONE*. 2009; doi: 10.1371/journal.pone.0007401.

16. Callahan BJ, McMurdie PJ, Holmes SP. Exact sequence variants should replace operational taxonomic units in marker-gene data analysis. *The ISME Journal*. 2017; doi: 10.1038/ismej.2017.119.

17. Parada AE, Needham DM, Fuhrman JA. Every base matters: assessing small subunit rRNA primers for marine microbiomes with mock communities, time series and global field samples: Primers for marine microbiome studies. *Environ Microbiol*. 2016; doi: 10.1111/1462-2920.13023.

18. Gonçalves RS, Musen MA. The variable quality of metadata about biological samples used in biomedical experiments. *Sci Data*. 2019; doi: 10.1038/sdata.2019.21.

19. HVRLocator (2026). HVRLocator (Version 1.0) https://github.com/fbcorrea/hvrlocator/.

20. Karsch-Mizrachi I, Nakamura Y, Cochrane G, on behalf of the International Nucleotide Sequence Database Collaboration. The International Nucleotide Sequence Database Collaboration. *Nucleic Acids Research*. 2012; doi: 10.1093/nar/gkr1006.

21. Leinonen R, Sugawara H, Shumway M, on behalf of the International Nucleotide Sequence Database Collaboration. The Sequence Read Archive. *Nucleic Acids Research*. 2011; doi: 10.1093/nar/gkq1019.

22. Chen S, Zhou Y, Chen Y, Gu J. fastp: an ultra-fast all-in-one FASTQ preprocessor. *Bioinformatics*. 2018; doi: 10.1093/bioinformatics/bty560.

23. Rognes T, Flouri T, Nichols B, Quince C, Mahé F. VSEARCH: a versatile open source tool for metagenomics. *PeerJ*. 2016; doi: 10.7717/peerj.2584.

24. Katoh K. MAFFT: a novel method for rapid multiple sequence alignment based on fast Fourier transform. *Nucleic Acids Research*. 2002; doi: 10.1093/nar/gkf436.

25. Brosius J, Palmer ML, Kennedy PJ, Noller HF. Complete nucleotide sequence of a 16S ribosomal RNA gene from Escherichia coli. *Proc Natl Acad Sci USA*. 1978; doi: 10.1073/pnas.75.10.4801.

26. Chen C, Liaw A, Breiman L. Using random forest to learn imbalanced data. *University of California, Berkeley*. 110:242004;

27. Saito T, Rehmsmeier M. The Precision-Recall Plot Is More Informative than the ROC Plot When Evaluating Binary Classifiers on Imbalanced Datasets. Brock G, editor. *PLoS ONE*. 2015; doi: 10.1371/journal.pone.0118432.

28. Mitra A, Skrzypczak M, Ginalski K, Rowicka M. Strategies for Achieving High Sequencing Accuracy for Low Diversity Samples and Avoiding Sample Bleeding Using Illumina Platform. Oudejans C, editor. *PLoS ONE*. 2015; doi: 10.1371/journal.pone.0120520.

29. Callahan BJ, McMurdie PJ, Rosen MJ, Han AW, Johnson AJA, Holmes SP. DADA2: High-resolution sample inference from Illumina amplicon data. *Nat Methods*. 2016; doi: 10.1038/nmeth.3869.

30. Das S, Biswas NK, Basu A. Mapinsights: deep exploration of quality issues and error profiles in high-throughput sequence data. *Nucleic Acids Research*. 2023; doi: 10.1093/nar/gkad539.

31. HVRLocator singularity container (2025). HVRLocator singularity container (Version 1.0) https://cloud.sylabs.io/library/jsaraiva/repo/hvrlocator.

32. Gilbert JA, Jansson JK, Knight R. The Earth Microbiome project: successes and aspirations. *BMC Biol*. 2014; doi: 10.1186/s12915-014-0069-1.

33. Varliero G, Lebre PH, Stevens MI, Czechowski P, Makhalanyane T, Cowan DA. The use of different 16S rRNA gene variable regions in biogeographical studies. *Environ Microbiol Rep*. 2023; doi: 10.1111/1758-2229.13145.

34. Jurburg S, et al.,. Microbial Community Database (MiCoDa). v1. https://micoda.idiv.de/. 2023; doi: 10.25829/na76.

35. Jurburg SD, Álvarez Blanco MJ, Chatzinotas A, Kazem A, König-Ries B, Babin D, et al.. Datathons: fostering equitability in data reuse in ecology. *Trends in Microbiology*. 2024; doi: 10.1016/j.tim.2024.02.010.

36. R Core Team. R: A language and environment for statistical computing. R Foundation for Statistical Computing, Vienna, Austria. *http://www R-project org/*. 2016;

37. GitHub Figures Scripts (2026). GitHub Figures Scripts (Version 1.0) https://github.com/ClarArboledaBaena/HVRLocator-Figures.

38. Karsch-Mizrachi I, Arita M, Burdett T, Cochrane G, Nakamura Y, Pruitt KD, et al.. The international nucleotide sequence database collaboration (INSDC): enhancing global participation. *Nucleic Acids Research*. Oxford University Press; 2025; doi: 10.1093/nar/gkae1058.

39. Crandall ED, Toczydlowski RH, Liggins L, Holmes AE, Ghoojaei M, Gaither MR, et al.. Metadata preservation and stewardship for genomic data is possible, but must happen now. *bioRxiv*. Cold Spring Harbor Laboratory; 2022; doi: 10.1101/2022.09.12.507034.

40. Ebou A, Koua D, Zeze A. HyperEx: A Tool to Extract Hypervariable Regions from 16S rRNA Sequencing Data. *bioRxiv*. 2021; doi: 10.1101/2021.09.03.455391.

41. Bolyen E, Rideout JR, Dillon MR, Bokulich NA, Abnet CC, Al-Ghalith GA, et al.. Reproducible, interactive, scalable and extensible microbiome data science using QIIME 2. *Nature biotechnology*. Nature Publishing Group US New York; 37:852–72019;

42. Langmead B, Salzberg SL. Fast gapped-read alignment with Bowtie 2. *Nature methods*. Nature Publishing Group; 9:357–92012;

43. Li H, Handsaker B, Wysoker A, Fennell T, Ruan J, Homer N, et al.. The sequence alignment/map format and SAMtools. *bioinformatics*. Oxford University Press; 25:2078–92009;

44. Edgar RC. Search and clustering orders of magnitude faster than BLAST. *Bioinformatics*. Oxford University Press; 26:2460–12010;

45. Shade A, Dunn RR, Blowes SA, Keil P, Bohannan BJM, Herrmann M, et al.. Macroecology to Unite All Life, Large and Small. *Trends in Ecology & Evolution*. 2018; doi: 10.1016/j.tree.2018.08.005.

46. Jones CB, White JR, Ernst SE, Sfanos KS, Peiffer LB. Incorporation of Data From Multiple Hypervariable Regions when Analyzing Bacterial 16S rRNA Gene Sequencing Data. *Front Genet*. 2022; doi: 10.3389/fgene.2022.799615.

47. Graham AS, Patel F, Little F, Van Der Kouwe A, Kaba M, Holmes MJ. Using short-read 16S rRNA sequencing of multiple variable regions to generate high-quality results to a species level. *Front Bioinform*. 2025; doi: 10.3389/fbinf.2025.1484113.

48. Arboleda-Baena C, Borim Correa F, Saraiva JP, Castillo-Rivadeneira S, Coelho Kasmanas J, Chatzinotas A, Jurburg SD. (2026) HVRLocator: A Computationally Efficient Tool for Identifying Hypervariable Regions in Large 16S rRNA Datasets. [DOME-ML Annotations]. DOME-ML Registry, https://registry.dome-ml.org/review/8g5af1ipg7

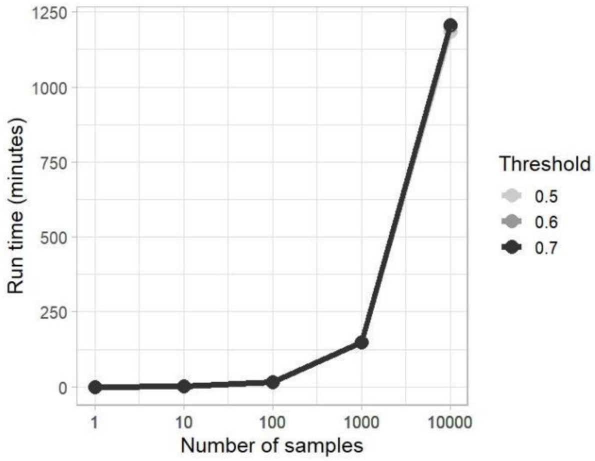

Figure 1. Relationship between the number of samples and run time (in minutes) using 8 GB of RAM and 4 CPU cores. We randomly selected sample numbers from the Earth Microbiome Project (Dataset 1), MiCoDa V1 (Dataset 2), and Datathon activities (Dataset 4). All samples were downloaded from the NCBI.

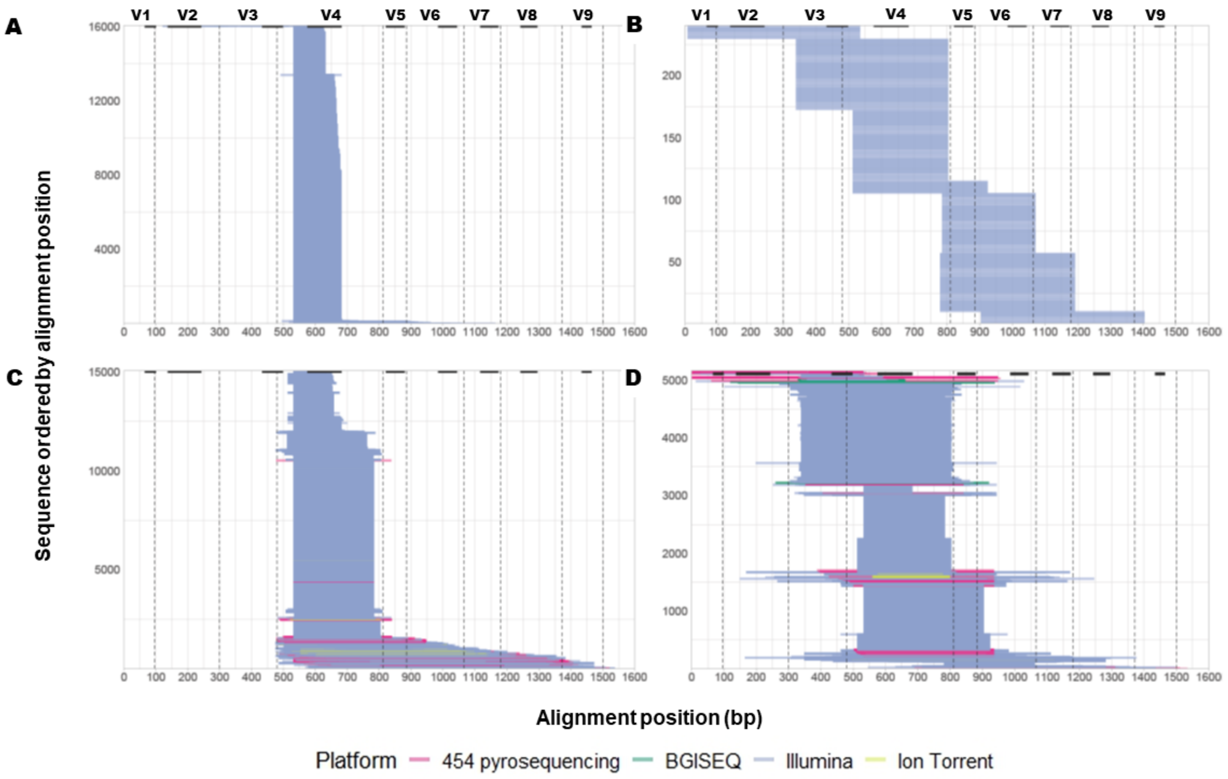

Figure 2. Alignment positions across 16S rRNA regions and sequencing setups. a) Same 16S rRNA region and sequencing setup (N = 16059 samples); b) Different 16S rRNA regions, same sequencing setup (N = 239 samples); c) Same 16S rRNA region, different sequencing setups (N = 15049 samples); d) Different 16S rRNA regions and sequencing setups (N = 5113 samples).The upper part of the figure, along with the dashed lines, indicates the start and end positions of the sequencing setups used to assign specific regions of the 16S rRNA gene (modified from Yang *et al.,* 2016). The hypervariable regions corresponding to each setup are highlighted with bold black bars (Brosius *et al.,* 1978).

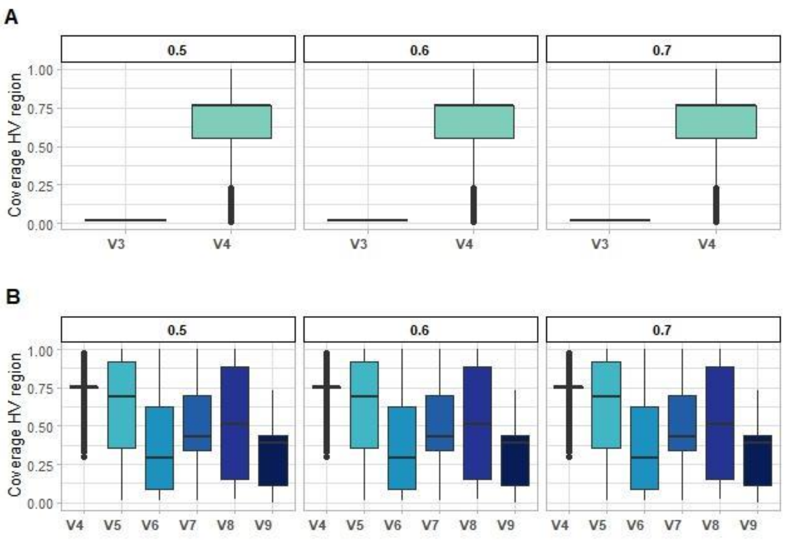

Figure 3. Differences in 16S rRNA gene coverage using the same primer set (Primer 515R-806R for V4 region) but different sequencing setups. A) Predicted 16S rRNA region coverage start, B) Predicted 16s rRNA region coverage end.

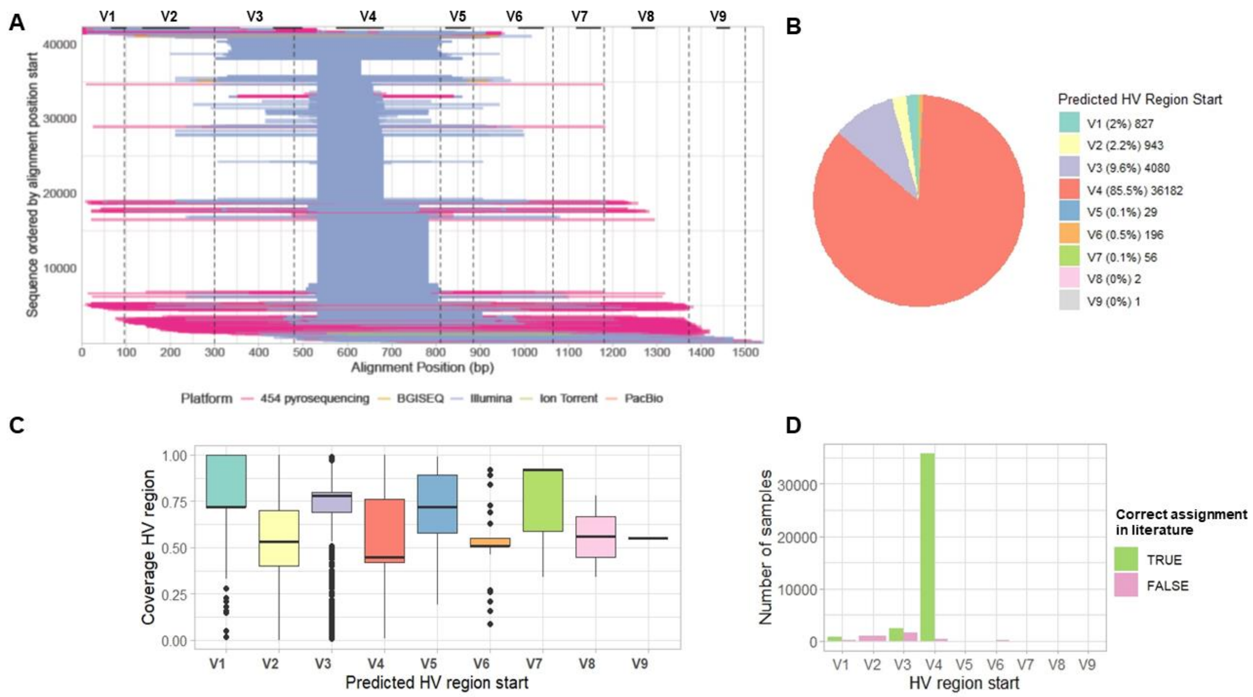

Figure 4. Application of HVRLocator for the selection of V4 16S rRNA gene metabarcoding samples from MiCoDa V2. A) Alignment start positions across the 16S rRNA gene for the 42,316 samples analyzed, B) Percentage of samples retained for downstream analyses after applying the HVRLocator tool, C) Variation in gene coverage across sequences, D) Number of samples in which primer assignment matched the metadata.

Figure1

Click here to access/download;Figure;Figure1_550_450.jpeg

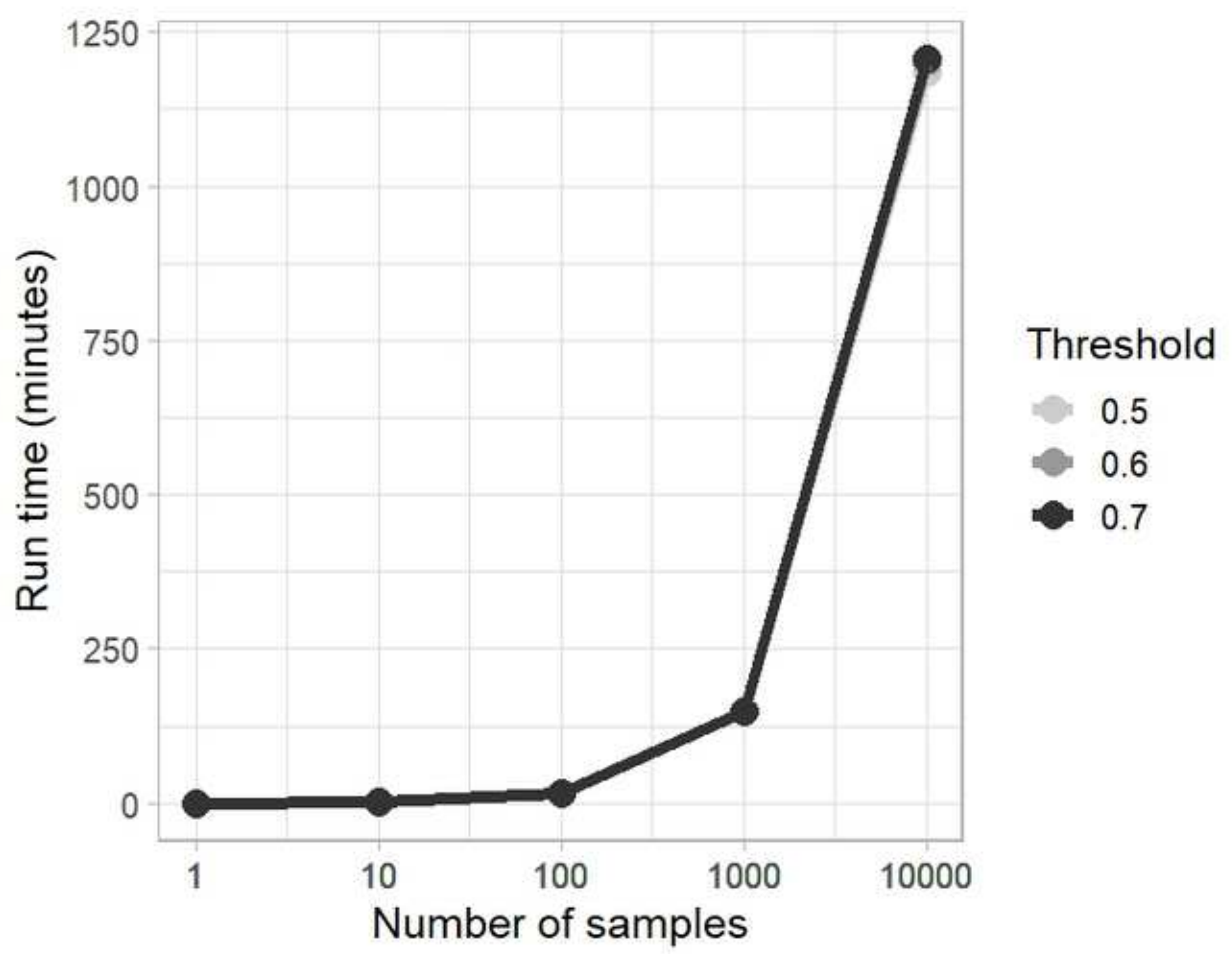

Figure2

Click here to access/download;Figure;Figure2.png

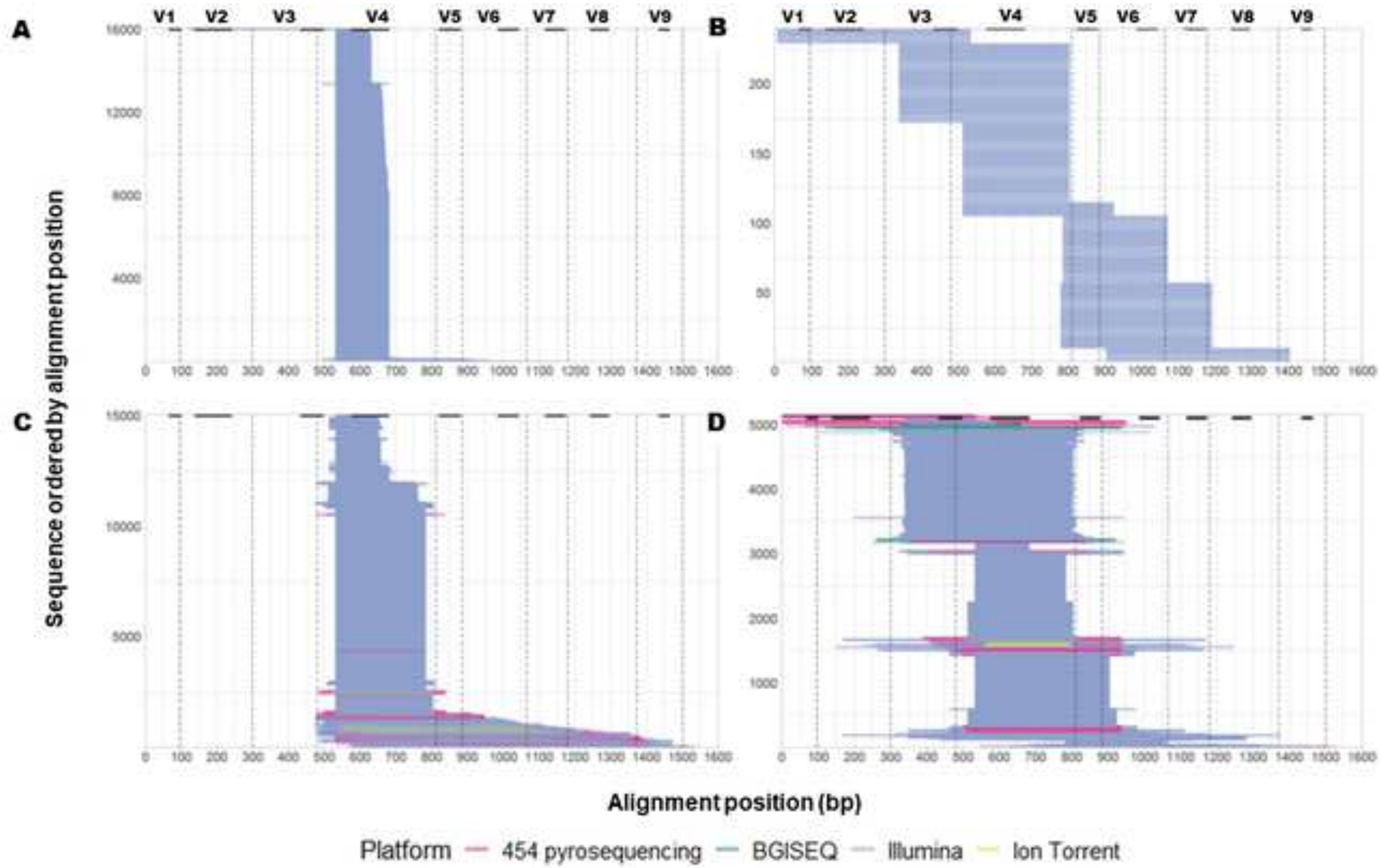

Figure3AB

Click here to access/download;Figure;Figure3AB_7_5.tif

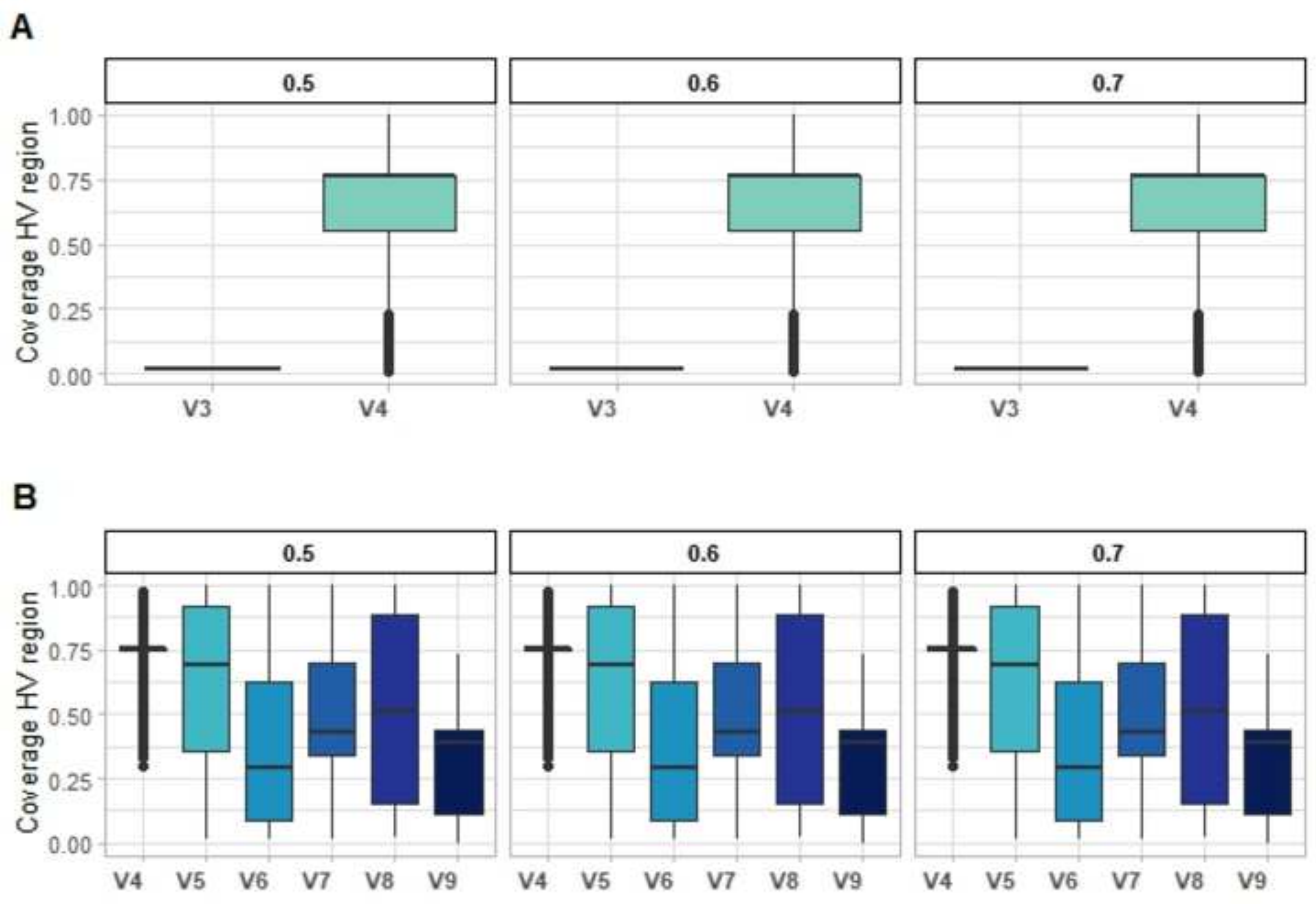

Figure4

Click here to access/download;Figure;Figure4_Final.JPG

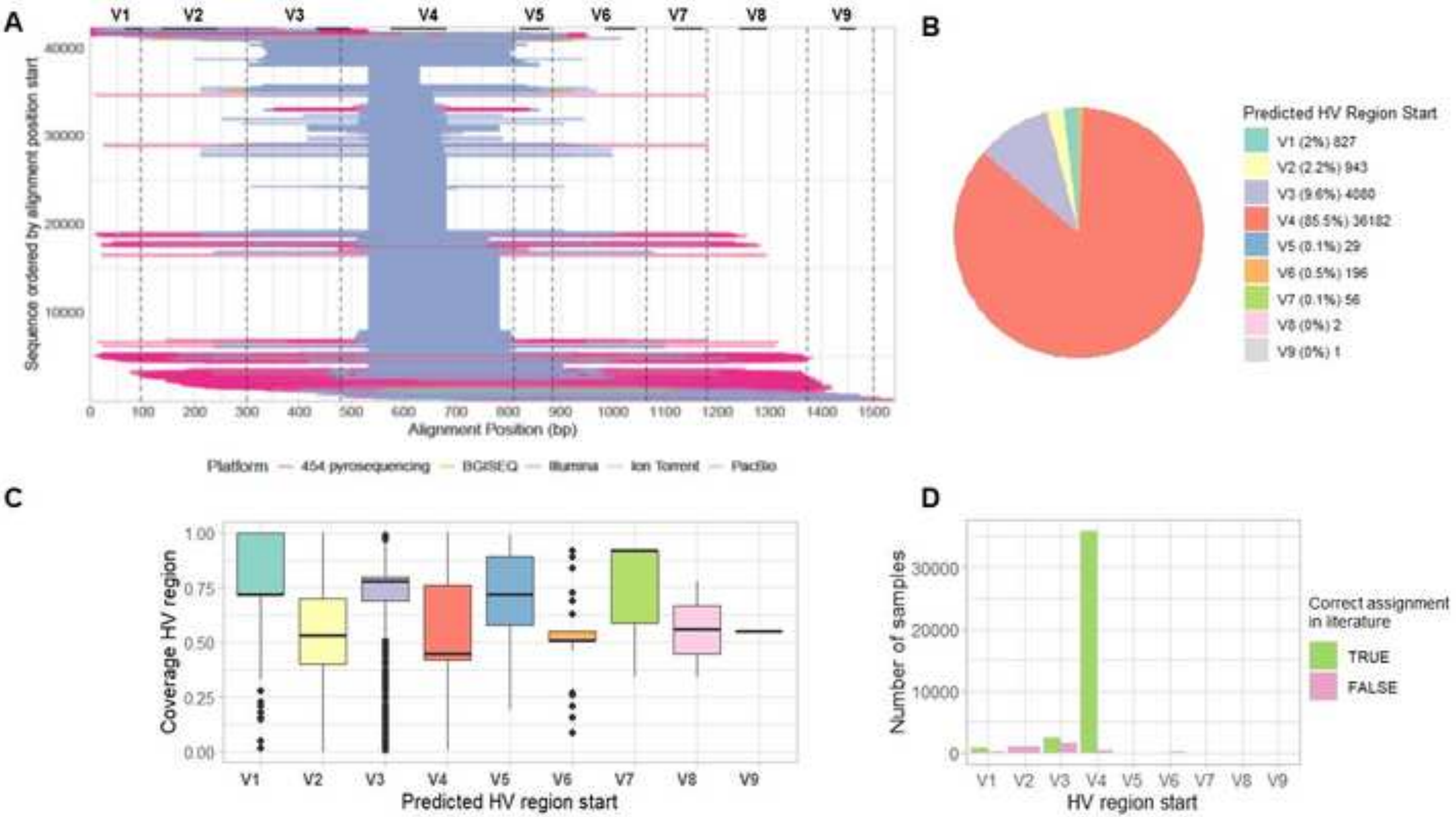

Click here to access/download
Supplementary Material
TableS6_Tool_performance_on_Archaeal_Eukaryotic_sequences.pdf

Click here to access/download
Supplementary Material
TableS7_Run_accession_list_for_case_study.csv

TableS8_CaseStudyOutput

Click here to access/download
Supplementary Material
TableS8_CaseStudyOutput.pdf

Click here to access/download
Supplementary Material
TableS9_CorrectAssignmentInLiterature.csv

Figure S1

Click here to access/download
Supplementary Material
Figure_S1.pdf

TableS1

Click here to access/download
Supplementary Material
TableS1.csv

TableS2

Click here to access/download
Supplementary Material
TableS2.csv

Click here to access/download
Supplementary Material
TableS3_Random_Forest_Model_for_Primer_Presence_
Prediction.pdf

Click here to access/download
Supplementary Material
TableS4_Dataset1_EarthMicrobiomeProject.csv

TableS4_Dataset2

Click here to access/download
Supplementary Material
TableS4_Dataset2_MiCoDaVersion1.csv

TableS4_Dataset3

Click here to access/download

Supplementary Material

TableS4_Dataset3_WasimuddinVarliero.csv

Click here to access/download

Supplementary Material

TableS4_Dataset4_Datathons.csv

Click here to access/download
Supplementary Material
TableS5_RunTime.pdf

Personal Cover

Click here to access/download;Personal Cover;PersonalCover_GIGA-D-25-00344R1.pdf

GIGA-D-25-00344R1
HVRLocator: A Computationally Efficient Tool for Identifying Hypervariable Regions in Large 16S rRNA Datasets.
Clara Arboleda-Baena; Felipe Borim Correa; Joao Pedro Saraiva; Santiago Castillo-Rivadeneira; Jonas Coelho Kasmanas; Antonis Chatzinotas; Stephanie D. Jurburg
GigaScience

Leipzig, 16 March 2026

Dear Dongni Ma,

I hope this message finds you well.

Please find attached our response to the reviewers regarding manuscript GIGA-D-25-00344R1: HVRLocator: A Computationally Efficient Tool for Identifying Hypervariable Regions in Large 16S rRNA Datasets.

We have addressed the final comment. It was not necessary to register a new software application in the bio.tools or SciCrunch.org databases to obtain RRID (Research Resource Identification Initiative ID) or biotoolsID identifiers.

We would like to thank you and the reviewers for the constructive feedback. We hope this version satisfactorily address the minor revision requests.

We look forward to your response.

Best wishes,

Dr. Clara Arboleda
On behalf of all co-authors

GIGA-D-25-00344R1
HVRLocator: A Computationally Efficient Tool for Identifying Hypervariable Regions in Large 16S rRNA Datasets
Clara Arboleda-Baena; Felipe Borim Correa; Joao Pedro Saraiva; Santiago Castillo-Rivadeneira; Jonas Coelho Kasmanas; Antonis Chatzinotas; Stephanie D. Jurburg
GigaScience

Dear Dr Arboleda-Baena,

Your manuscript "HVRLocator: A Computationally Efficient Tool for Identifying Hypervariable Regions in Large 16S rRNA Datasets" (GIGA-D-25-00344R1) has been assessed by our reviewers. Based on these reports, and my own assessment as Editor, I am pleased to inform you that it is potentially acceptable for publication in GigaScience, once you have carried out some essential revisions suggested by our reviewers.

Their reports, together with any other comments, are below. Please also take a moment to check our website at https://www.editorialmanager.com/giga/ for any additional comments that were saved as attachments.

In addition, please register any new software application in the bio.tools and SciCrunch.org databases to receive RRID (Research Resource Identification Initiative ID) and biotoolsID identifiers, and include these in your manuscript. Computational workflows should be registered in workflowhub.eu and the DOIs cited in the relevant places in the manuscript. These will facilitate tracking, reproducibility and re-use of your tool.

Once you have made the necessary corrections, please submit a revised manuscript online at:

https://www.editorialmanager.com/giga/

If you have forgotten your username or password please use the "Send Login Details" link to get your login information. For security reasons, your password will be reset.

Please include a point-by-point within the 'Response to Reviewers' box in the submission system. Please ensure you describe additional experiments that were carried out and include a detailed rebuttal of any criticisms or requested revisions that you disagreed with. Please also ensure that your revised manuscript conforms to the journal style, which can be found in the Instructions for Authors on the journal homepage. If the data and code has been modified in the revision process please be sure to update the public versions of this too.

The due date for submitting the revised version of your article is 27 Mar 2026.

We look forward to receiving your revised manuscript soon.

Best wishes,

Dongni Ma, Ph.D.
GigaScience
www.gigasciencejournal.com

R/ Thank you very much, attached you can find the Response to Reviewers.

Reviewer reports:
Reviewer #1: I believe that the authors have addressed the concerns that I raised - in particular the question about whether using a single E. coli sequence would detect divergent bacteria which was raised by the other referee too. If I have understood their methodology correctly they are characterising a sample at a time and while there may be some individual sequences that do not "match" [sensu lato] E. coli there will almost always be sequences that are sufficiently similar to E. coli for their method to work. In their response to referees, the authors say:

"Even for bacterial taxa that are highly divergent from E. coli, the 16S rRNA gene remains highly conserved across Bacteria, ensuring sufficient sequence similarity for reliable alignment to the appropriate reference region. Although 16S rRNA sequences vary in length among taxa, our validation showed 99% agreement between the predicted hypervariable regions and those reported in the original publications (metadata or NCBI records). Together, the conserved structure of the 16S rRNA gene and the strong empirical validation support the conclusion that the current alignment strategy provides reliable estimates of start and end positions for hypervariable regions in Bacteria and adequately addresses the objectives of this study."

I would reccommend that the sense of this response is included more explicitly into the final manuscript, perhaps with a cross reference to supplementary material, and that the authors check through their other responses to the referees to make sure that the arguments that they provided there are included within the main part of the manuscript.

R/ We reviewed the responses in the previous file "Response to Reviewers_GIGA-D-25-00344" and found that most of the reviewers' concerns corresponded to comments that had already been addressed in the main manuscript. However, we have taken the reviewer's final suggestion into account and added the following text to the main manuscript:

Lines 275-283: "Importantly, validation highlighted HVRLocator tool's ability to identify problematic sequences. For example, we observed 932 samples with abnormally long average sequence lengths (i.e., >600 bp) that exceeded the expected output lengths with Illumina platforms. Upon manually reviewing these sequences, we found that either the sequencing platform was incorrectly annotated in the metadata (NCBI or the associated publication), or the sequences did not correspond to the 16S rRNA gene but rather to the Internal Transcribed Spacer (ITS) region or the nifH gene. This highlights the use of HVRLocator as a curation tool for large datasets, where human errors in annotation can significantly impact downstream analysis. For further details, see **Supplementary Table S6**."

We also added a new supplementary table (**Supplementary Table S6**), which contains the following:

Even for bacterial taxa that are highly divergent from E. coli, the 16S rRNA gene remains highly conserved across Bacteria, ensuring sufficient sequence similarity for reliable alignment to the appropriate reference region. Although 16S rRNA sequences vary in length among taxa, our validation showed 99% agreement between the predicted hypervariable regions and those reported in the original publications (metadata or NCBI records). Together, the conserved structure of the 16S rRNA gene and the strong empirical validation support the conclusion that the current alignment strategy provides reliable estimates of start and end positions for hypervariable regions in Bacteria and adequately addresses the objectives of this study.

Regarding Archaea and Eukaryotes, we performed several tests and identified potential warning signs that may alert users to review the metadata of sequences exhibiting the following patterns:

1. Putative ITS region: Sequences showing coverage across more than four or five hypervariable (HV) regions. In such cases, users should verify the following information in the metadata: the sequencing platform. If the platform used for these sequences is not PacBio or Nanopore, the primers may be targeting regions that are not specific to bacteria. If the mentioned platforms were used, users should also verify that the primers are not targeting Archaea or Eukaryotes.

The following table presents examples of ITS regions analyzed using HVRLocator:

Sample_ID	Primer Presence	Score Primer Presence	Min Alignment start	Max Alignment end	Average Alignment start	Average Alignment end	Median Alignment start	Median Alignment end	Predicted HV region start	Predicted HV region end	Coverage based HV region start	Coverage based HV region end	Coverage HV region start	Coverage HV region end	Warnings
SRR24285816	FALSE	0.17	0	1540	374	1068	399	1148	V3	V7	V4	V7	0.45	0.72	V3 below threshold of 0.5
SRR24285817	FALSE	0.17	0	1540	398	992	399	1110	V3	V7	V4	V6	0.45	0.39	V3 and V7 below threshold of 0.5
SRR24285818	FALSE	0.17	0	1540	400	1018	399	1110	V3	V7	V4	V6	0.45	0.39	V3 and V7 below threshold of 0.5
SRR24285819	FALSE	0.17	0	1540	402	994	399	1110	V3	V7	V4	V6	0.45	0.39	V3 and V7 below threshold of 0.5
SRR24285820	FALSE	0.17	0	1540	446	1017	399	1110	V3	V7	V4	V6	0.45	0.39	V3 and V7 below threshold of 0.5
SRR24285821	FALSE	0.16	0	1540	431	1000	399	1110	V3	V7	V4	V6	0.45	0.39	V3 and V7 below threshold of 0.5
SRR24285822	FALSE	0.17	0	1540	374	953	399	1110	V3	V7	V4	V6	0.45	0.39	V3 and V7 below threshold of 0.5
SRR24285823	FALSE	0.17	0	1540	392	961	399	1010	V3	V6	V4	V6	0.45	0.69	V3 below threshold of 0.5
SRR24285824	FALSE	0.17	0	1540	392	968	399	953	V3	V6	V4	V5	0.45	0.38	V3 and V6 below threshold of 0.5
SRR24285825	FALSE	0.17	0	1540	401	963	399	953	V3	V6	V4	V5	0.45	0.38	V3 and V6 below threshold of 0.5
SRR25665816	FALSE	0.17	0	1540	357	872	186	826	V2	V5	V2	V4	0.56	0.2	V5 below threshold of 0.5
SRR25665819	FALSE	0.2	0	1540	690	1172	903	1450	V6	V9	V6	V8	0.91	0.34	V9 below threshold of 0.5
SRR25665821	FALSE	0.22	0	1540	425	956	186	826	V2	V5	V2	V4	0.56	0.2	V5 below threshold of 0.5
SRR25665824	FALSE	0.2	0	1540	627	1210	685	1210	V4	V8	V5	V7	0.38	0.16	V4 and V8 below threshold of 0.5
SRR25665826	FALSE	0.22	0	1540	602	1118	574	1048	V4	V6	V4	V6	0.72	0.91	NA
SRR25665828	FALSE	0.2	0	1540	303	1164	177	1096	V2	V7	V2	V6	0.61	0.27	V7 below threshold of 0.5
SRR25665830	FALSE	0.17	0	1540	600	1149	529	1048	V4	V6	V4	V6	0.85	0.91	NA
SRR25665832	FALSE	0.21	0	1540	449	970	186	826	V2	V5	V2	V4	0.56	0.2	V5 below threshold of 0.5

**ERR5924913**	**FALSE**	**0.17**	**0**	**1540**	**482**	**1055**	**548**	**1111**	**V4**	**V7**	**V4**	**V6**	**0.8**	**0.4**	**V7 below threshold of 0.5**
**ERR5924916**	**FALSE**	**0.17**	**0**	**1540**	**463**	**1076**	**595**	**1177**	**V4**	**V7**	**V4**	**V7**	**0.65**	**0.97**	**NA**

2. Putative Archaea or 18S hypervariable region: As clarified in the manuscript, users must ensure that the target domain is Bacteria. Due to similarities in gene length and conserved regions, sequences targeting archaeal 16S rRNA genes or eukaryotic 18S rRNA genes may still produce a prediction when analyzed with HVRLocator. However, the tool is specifically designed and validated for bacterial 16S rRNA sequences. Therefore, verification of the sample metadata is essential to confirm that the predicted hypervariable region corresponds to bacterial sequences.

Reviewer #2: The authors have made proactive improvements in both the functional flexibility of the program (supporting custom reference sequences) and the expansion of the taxonomic scope (mentioning future support for ITS, etc.); therefore, this manuscript would be of significant value for publication as a Technical Note in GigaScience.

R/ We thank the reviewer for their positive feedback.

--

Please also take a moment to check our website at https://www.editorialmanager.com/giga/l.asp?i=242341&l=0KJITSI3 for any additional comments that were saved as attachments. Please note that as GigaScience has a policy of open peer review, you will be able to see the names of the reviewers.

R/ Done, thank you.
