# Supplementary material for: HVRLocator: a computationally efficient tool for identifying hypervariable regions in large 16S rRNA datasets
Source: Gigascience. 2026 Apr 8;15:giag040. doi: 10.1093/gigascience/giag040 (PMC13188219; doi:10.1093/gigascience/giag040)
Supplement: giag040_Authors_Response_To_Reviewer_Comments_original_submission [file giag040_authors_response_to_reviewer_comments_original_submission.pdf]

Author's Response To Reviewer Comments

GIGA-D-25-00344
HVRLocator: A Computationally Efficient Tool for Identifying Hypervariable Regions in Large 16S rRNA Datasets
Clara Arboleda-Baena; Felipe Borim Correa; Joao Pedro Saraiva; Santiago Castillo-Rivadeneira; Jonas Coelho Kasmanas; Antonis Chatzinotas; Stephanie D. Jurburg
GigaScience

Leipzig, 17 February 2026

Dear Dongni Ma,

I hope this message finds you well.

Please find attached our response to the reviewers regarding manuscript GIGA-D-25-00344: HVRLocator: A Computationally Efficient Tool for Identifying Hypervariable Regions in Large 16S rRNA Datasets. We have addressed all comments. We would like to thank you and the reviewers for the constructive feedback, which has helped us improve the manuscript and clarify our message. We hope that the revisions satisfactorily address the minor revision requests.

As outlined in the response document, it was not necessary to register a new software application in the bio.tools or SciCrunch.org databases to obtain RRID (Research Resource Identification Initiative ID) or biotoolsID identifiers.

We have now also uploaded the scripts used in our analyses to GitHub, as suggested by one of the reviewers.

We look forward to your response.

Best wishes,

Dr. Clara Arboleda
On behalf of all co-authors

Reviewer reports:

Reviewer #1: Metabarcoding data are accumulating rapidly. This paper makes a very valuable contribution to the automated extraction and curation of metabarcoding data and should be of great value in facilitating the re-use of existing data and the construction of custom databases based on these.
I have not tested or tried to install the software myself, as the manuscript provided sufficient detail to enable me to assess the tool

R/ We thank the reviewer for their positive feedback.

General comments:

The manuscript is written entirely in terms of "bacteria" and aligns amplicons to an E. coli model sequence. This is reasonable, but there should certainly be some acknowledgement of Archaea and ideally some mention of Eukaryotes too. These are probably things for the discussion section of this manuscript, but the authors may wish to consider whether a future version of the program could contain options to use model Archaea and Eukaryote sequences as alternatives to the E. coli model.

R/ Thank you for the comment. We acknowledge that this software may be useful for researchers working with metabarcoding data from other realms of life. This is foreseen by the software, which allows the user to input their own reference sequence and map against it, offering that flexibility. We now refer to this in Lines 389-393. While this manuscript focuses on the evaluation of E. coli for usage against 16S rRNA gene data, we are currently benchmarking its use for fungi (ITS region) for future versions, although this is beyond the scope of the present work. For clarity, we have added the following text to the manuscript:

Lines 389-393: “Although the present work focuses on bacteria, we acknowledge that other domains of life, including Archaea and Eukaryotes, are relevant for future meta-analyses and synthesis studies. Consequently, future versions of the program aim to incorporate alternative model sequences representing Archaea and Eukaryota.”

It would also be helpful to assess how the program with its E. coli model deals with sequence data from Archaea, Eukaryotes (including mitochondria) and bacteria that are very divergent from E. coli.

R/ Thank you for the comment. Even for bacterial taxa that are highly divergent from E. coli, the 16S rRNA gene remains highly conserved across Bacteria, ensuring sufficient sequence similarity for reliable alignment to the appropriate reference region. Although 16S rRNA sequences vary in length among taxa, our validation showed 99% agreement between the predicted hypervariable regions and those reported in the original publications (metadata or NCBI records). Together, the conserved structure of the 16S rRNA gene and the strong empirical validation support the conclusion that the current alignment strategy provides reliable estimates of start and end positions for hypervariable regions in Bacteria and adequately addresses the objectives of this study.

Regarding Archaea and Eukaryotes, we performed several tests and identified potential warning signs that may alert users to review the metadata of sequences exhibiting the following patterns:

1. Putative ITS region: Sequences showing coverage across more than four or five hypervariable (HV) regions. In such cases, users should verify the following information in the metadata: the sequencing platform. If the platform used for these sequences is not PacBio or Nanopore, the primers may be targeting regions that are not specific to bacteria. If the mentioned platforms were used, users should also verify that the primers are not targeting Archaea or Eukaryotes.

The following table presents examples of ITS regions analyzed using HVRLocator:

2. Putative Archaea or 18S hypervariable region: As clarified in the manuscript, users must ensure that the target domain is Bacteria. Due to similarities in gene length and conserved regions, sequences targeting archaeal 16S rRNA genes or eukaryotic 18S rRNA genes may still produce a prediction when analyzed with HVRLocator. However, the tool is specifically designed and validated for bacterial 16S rRNA sequences. Therefore, verification of the sample metadata is essential to confirm that the predicted hypervariable region corresponds to bacterial sequences.

Finally, we have included the following statement in the main text to clarify this point for users:

Lines 275-283: “Importantly, validation highlighted HVRLocator tool’s ability to identify problematic sequences. For example, we observed 932 samples with abnormally long average sequence lengths (i.e., >600 bp) that exceeded the expected output lengths with Illumina platforms. Upon manually reviewing these sequences, we found that either the sequencing platform was incorrectly annotated in the metadata

(NCBI or the associated publication), or the sequences did not correspond to the 16S rRNA gene but rather to the Internal Transcribed Spacer (ITS) region or the nifH gene. This highlights the use of HVRLocator as a curation tool for large datasets, where human errors in annotation can significantly impact downstream analysis."

Lines 389-393: "Although the present work focuses on bacteria, we acknowledge that other domains of life, including Archaea and Eukaryotes, are relevant for future meta-analyses and synthesis studies. Consequently, future versions of the program aim to incorporate alternative model sequences representing Archaea and Eukaryota"

The methods section does not contain details of software used to generate the figures, or whether these figures are produced by "the pipeline" or by separate analysis of the .txt file that the pipeline produces. I suspect that it is that latter, in which case making the authors should make the scripts used available - as well as providing complete documentation of what has been done, this is likely to increase use made of the tool.
And it would be helpful to include an output file in the supplementary materials.

R/ Thank you for the recommendation. We have now made all scripts and tables available on GitHub. The following text has been included in the Methods section:

"All graphics were carried out in R with RStudio interface (R Core Team, 2016), and all pipelines are available at: https://github.com/ClarArboledaBaena/HVRLocator-Figures."

Specific comments

Line 64 "however the integration of these data in light of processing metadata" - not clear

R/ Thank you for noting this. The correct phrase is: "however, the integration of these data in the context of Big Data processing and synthesis".

Line 67-8 "though bacterial diversity increases linearly with amplicon length".
Needs re-wording. The number of ASVs will increase with amplicon length, but the actual bacterial diversity in a sample is constant.

R/ Thank you for your comment. We have modified it to "though detected bacterial diversity increases linearly with amplicon length".

Line 79 "Wasimuddin and colleagues" should be "Wasimuddin et al". More generally, check that citations conform with journal house style

R/ Done

Line 79-82 "For example, Wasimuddin and colleagues [8] found that compared to three other primer sets targeting different regions, the primer pair targeting the V4 hypervariable region of the 16S rRNA gene produced the highest estimates of species richness and diversity across various sample types"
There are three issues here:
1) different primer pairs vary in their coverage and bias, so different primers targeting the same variable region will produce different numbers of ASVs
2) Even with complete coverage and the absence of bias, different variable regions will generate different numbers of ASVs as a result of differences in length and rate of evolution between variable regions (and differences in the number of ASVs that are clustered into OTUs at a particular sequence similarity threshold
3) The relationship between ASVs or OTUs and "species" is not straightforward (Edgar, 2018).
At minimum "species" should be replace with ASV or OTU (whichever Wasimuddin et al used)
Edgar, R. C. (2018). Updating the 97% identity threshold for 16S ribosomal RNA OTUs. Bioinformatics, 34(14), 2371-2375. doi:10.1093/bioinformatics/bty113

R/ Yes, the reviewer is correct regarding all three points. Accordingly, we have replaced the term "species"

with “Amplicon Sequence Variants (ASVs)” and added the following sentence:

Lines 83-85: “However, different primers targeting the same variable region can still generate different numbers of ASVs (Parada et al., 2016)”

Line 89-90 "as bacterial diversity and taxonomic resolution linearly increase with target sequence length [12]." Overlaps with statements made in line 67-8, and the same issue applies here.

R/ Thank you for pointing this out. We agree that this statement overlaps with the one in lines 67–68. To avoid redundancy, we have rephrased lines 89–90 to better differentiate the concepts and improve clarity:

“Variation in amplicon length complicates the reuse of 16S rRNA metabarcoding data, since longer sequences tend to detect greater bacterial diversity and taxonomic resolution”

Lines 167-170. The output file contains (amongst other things) "Predicted HV region Start/End: Predicted hypervariable (HV) region based on the median alignment start and end positions across all reads, inferred from literature on conserved and hypervariable regions of the 16S rRNA gene (Brosius et al., 1978; Yang et al., 2016)". This implies that the program predicts a single variable region for each study. I am not clear what this column will contain for amplicons that contain more than one variable region, although columns 11-19 indicate that the program identifies the presence/absence of each of the 9 HV regions.

R/ Thank you very much for this helpful comment. For clarification, HVRLocator predicts a single hypervariable region for each sample, rather than for each study, as mentioned by the reviewer. As correctly pointed out, some studies (BioProjects) include samples spanning multiple hypervariable regions, a situation that has become increasingly common with the advent of long-read sequencing technologies.

In addition to identifying the hypervariable region corresponding to the average and median start and end positions of the amplicons within each sample, HVRLocator also reports the coverage across all nine hypervariable regions for every individual sample. In this way, users can evaluate the amplicon length, its exact start and end positions, and the coverage of specific hypervariable region(s) within each sample.

We have added the following sentence in the main manuscript to clarify this point for users (Lines 201-205): ”Finally, in addition to identifying the hypervariable region based on the average and median start and end positions of the amplicons within each sample, HVRLocator also reports coverage across all nine hypervariable regions for every individual sample. This allows users to assess the amplicon length, determine its exact start and end positions, and evaluate the coverage of specific hypervariable region(s) within each sample.”

Regarding studies that include samples with different hypervariable region coverage, we aimed to illustrate this scenario using Dataset B, which comprises 242 samples from two studies that compared different primer sets but were sequenced on the same platform (Wasimuddin et al., 2020; Varliero et al., 2023). As shown in Figure 2B, HVRLocator successfully predicts different variable regions within the same study.

My guess is that the authors are using "HV region" in two different sense:
1) Its usual meaning of one region out of V1 to V9
2) The sequence from the beginning of the first of the nine variable regions the amplicon includes to the end of the last.
It would also be helpful to indicate whether the sequence positions here are relative to the E coli model or refer to sequence positions in the amplicon

R/ Thank you for the comment. For clarification, we use the term “HV region” according to option (1), that is, its standard meaning as one of the regions V1–V9. To address the reviewer’s concern, we will provide an example illustrating the HVRLocator output and demonstrate how the information produced by the tool resolves the points raised:

The following table shows the HVRLocator output for five samples from the same study (Varliero et al., 2023). As shown, even within the same study, the tool predicts different HV regions for different samples.

Output (only 8 columns shown):

We are going to explain the first sample:

For sample ERR10042760, the average and median alignment start positions are 8 and 7, respectively. These values represent the sequence start positions relative to the E. coli reference model, as mentioned in your comment. As you can see the predicted HV region where this amplicon starts for this sample is V1. The average and median alignment end positions are 537 and 534, respectively. These values indicate the sequence end positions relative to the E. coli reference model. The predicted hypervariable (HV) region at which this amplicon ends is V4.

This indicates that the samples cover HV regions V1, V2, V3, and V4. This can be verified by examining the remaining columns of the output.

Output (only 14 columns shown):

The tool further shows that the amplicon provides full coverage of HV regions V1 to V3. This is reflected in the columns “Cov_V1” to “Cov_V9,” which report coverage values (ranging from 0 to 1) for each HV region. Coverage values close to or equal to 1 are observed for HV regions V1–V3, whereas a lower value is observed for V4.

This indicates that sample ERR10042760 starts in HV region V1 and ends in HV region V4, fully covering HV regions V1–V3, while HV region V4 is only partially covered. This information allows users to interpret the output and make informed decisions in downstream analysis pipelines.

Reviewer #2: General Comments
This manuscript introduces a tool named HVRLocator, designed to address the issue of missing or non-

standard metadata in 16S rRNA sequencing data found in public databases such as the SRA. The tool identifies amplicon regions by aligning sequences to a reference genome and attempts to detect the presence of primers using a machine learning model. This is a subject with significant practical value, particularly for conducting large-scale meta-analyses. However, there are still many issues regarding methodological rigor, the depth of validation, and comparisons with existing tools that require further clarification by the authors.

R/ We address the reviewer's concerns regarding methodological rigor, the depth of validation, and comparisons with existing tools in the following comments.

Major Comments

1. Concerns regarding the singularity of the reference sequence
A.The authors mention aligning sequences to a single Escherichia coli (J01859.1) reference genome to determine start and end positions. Is a single E. coli reference sufficient to cover Archaea or bacterial phyla that are distantly related to Proteobacteria, which may be present in environmental samples (e.g., soil, ocean)?

R/ HVRLocator aims to identify primers designed to target Bacteria only, not Archaea, although We acknowledge that bacterial primers amplify non-target 16S rRNA from Archaea. Crucially, this software is designed to work with metabarcoding data, where the composition of the sequenced community is usually unknown a priori, so it is possible and expected that when distantly-related taxa are present in the samples, some reads will not map to E. coli as well as others. This is why we designed HVRLocator to systematically download 10,000 reads per sample to assess the mapping of these reads against our model E. coli on average. We see no indication of the presence of distantly related organisms in a sample affecting our ability to infer the target region sequenced, as confirmed by our assessment of the region sequenced across the methods stated in the literature for a subset of our samples.
We acknowledge that the ability to change the mapping sequence is useful, and would greatly expand the functionality of HVRLocator, and have built the tool foreseeing this expansion in the future, and we know acknowledge this in Line 389-393, although the accuracy against other mapping sequences is beyond the scope of this paper. Indeed, we are currently testing it for fungal studies targeting the ITS region, and hope to release this new version in the coming years, after rigorous testing.

Lines 389-393: "Although the present work focuses on bacteria, we acknowledge that other domains of life, including Archaea and Eukaryotes, are relevant for future meta-analyses and synthesis studies. Consequently, future versions of the program aim to incorporate alternative model sequences representing Archaea and Eukaryota."

B. For taxa with significant length variations or insertions/deletions (Indels), could forced alignment to the E. coli reference lead to misjudgment of start/end positions?

R/ We do not consider length variation to be a major issue, as HVRLocator aligns relatively short amplicons to the full-length E. coli 16S rRNA reference sequence. Although some taxa exhibit insertions/deletions (indels) or length variation, the 16S rRNA gene is highly conserved across Bacteria, ensuring sufficient sequence similarity for reliable alignment to the appropriate reference region.
While 16S rRNA sequences vary in length across taxa, our validation showed 99% agreement between predicted hypervariable regions and those reported in the original publications (metadata or NCBI records). Together, the conserved structure of the 16S rRNA gene and the strong empirical validation support that the current alignment strategy provides reliable start and end position estimates for hypervariable regions and adequately addresses the objectives of this study.

C. Have the authors evaluated the impact on accuracy if a more universal reference database (such as representative sequences from SILVA or Greengenes) were used?

R/ We did not evaluate the impact of using a more universal reference database, as the current approach yielded accurate, consistent, and importantly, efficient results for the purposes of this study. However, we agree that exploring the use of representative sequences from broader reference databases such as SILVA

or Greengenes could be valuable, and we will consider this for future versions of the tool.

2. Rationality of the primer detection model (Random Forest based on Quality Scores)
2.1 The authors developed a Random Forest model to predict primer presence by analyzing the quality score distribution of the first 1,000 reads. Primer detection is typically based on the sequence itself rather than quality scores. Can the authors explain why quality scores were chosen as features?

R/ We appreciate the reviewer's point and welcome the opportunity to clarify our rationale. Illumina sequencing is known to generate distinct quality score patterns in the first few cycles when base diversity is low, as occurs when untrimmed primers are present at the start of all reads (Mitra et al., 2015). Thus, we chose to use quality scores as features because they serve as a practical proxy for detecting primer presence. This approach allows for accurate, sequence-agnostic primer detection, especially when the actual primer sequences used are unknown or highly variable across datasets, which is the case in synthesis work, and a key reason for our development of HVRLocator. We have added text in lines 139-145 in the manuscript indicating our rationale:

Lines 139-145: "RF performs well under moderate class imbalances when class-aware evaluation and sampling is applied [25,26]. Additionally, distinct quality score patterns are known to occur in the first few cycles of Illumina sequencing when base diversity is low [27] as is in the case of untrimmed primers at the start of reads. Quality score patterns have also been used to detect sequencing bias and artifacts by tools such as DADA2 [28] and Mapinsights [29]. Thus, this metric can serve as a proxy for detecting primer presence."

Mitra A. et al. (2015). "Strategies for Achieving High Sequencing Accuracy for Low Diversity Samples…"

2.2 Sequencing quality scores are influenced by technical factors such as sequencer status, reagent batches, and run cycles, which have no direct biological correlation with the presence of primers. Is there a risk that this model is "overfitting" specific sequencing platforms or datasets?

R/ This is a valid concern, and we took several steps to mitigate the risk of platform-specific overfitting. First, our training dataset included thousands of SRA runs generated from a range of sequencing platforms and protocols. Second, during validation, we tested the model across multiple independent datasets, including those with differing primer sets and sequencing technologies, and achieved consistently high performance (precision and recall >99%) regardless of platform. Third, we ensured that the features used generalize well across datasets by using stratified sampling and fixed model parameters. Similar uses of quality score patterns to model sequencing bias and detect artifacts have been validated in tools like DADA2 (Callahan et al., 2016) and Mapinsights (Das et al., 2023), supporting the robustness of such approaches. Our observed performance across diverse conditions suggests the model is not overfitting. We have now added text in the manuscript (lines 139-145) highlighting the use of quality score patterns by publicly available tools:

Lines 139-145: "RF performs well under moderate class imbalances when class-aware evaluation and sampling is applied [25,26]. Additionally, distinct quality score patterns are known to occur in the first few cycles of Illumina sequencing when base diversity is low [27] as is in the case of untrimmed primers at the start of reads. Quality score patterns have also been used to detect sequencing bias and artifacts by tools such as DADA2 [28] and Mapinsights [29]. Thus, this metric can serve as a proxy for detecting primer presence."

Das S. et al. (2023). "Mapinsights: deep exploration of quality issues and error profiles in high-throughput sequence data."

Callahan B. et al. (2016). "DADA2: High resolution sample inference from Illumina amplicon data"

Since the reads are already downloaded, why not directly use degenerate primer sequence matching (e.g., using Cutadapt or SeqKit logic) to determine primer presence? This seems to be a more direct and accurate method.

R/ Degenerate sequence matching (e.g., via Cutadapt or SeqKit) requires prior knowledge of all potential primer sequences used across datasets. This poses a challenge for large-scale curation of public datasets, which often lack standardized or complete primer metadata, especially when INSDC databases often lack a link between the publication and the data for a substantial portion of the datasets archived. Our method provides a generalizable and lightweight solution that does not rely on prior knowledge of primer sequences (mentioned in lines 358-364 of the manuscript). By bypassing primer-specific matching, we reduce computational cost and avoid the risk of missing primer variants due to sequence mismatches or degeneracy. We view our workflow as a complementary tool, particularly useful in high-throughput or poorly annotated datasets.

Line 358-364: "In contrast, HVRLocator does not rely on simulations or a priori primer information, and is designed for its application to existing datasets. HVRLocator operates directly on large, INSDC-archived metabarcoding datasets to identify the start and end positions of sequenced 16S rRNA amplicons, determine their corresponding hypervariable regions, and detect the presence primer sequences, generating the technical metadata that is needed for bioinformatics processing of the raw sequences."

3. Verification of accuracy claims
In the validation section, the authors claim to achieve 100% accuracy on certain datasets. In bioinformatics tool development, a claim of 100% accuracy is often a red flag. Have the authors manually checked those samples marked as "correct" by the model that might suffer from edge effects or borderline cases?

R/ We fully agree that claims of 100% accuracy require scrutiny. This value refers to specific controlled datasets where the sequenced region and primer pairs were known from curated sources (e.g., MiCoDa). Regardless, we manually reviewed edge cases and anomalies flagged by our tool such as sequences with unusual read lengths or misannotated primer regions and confirmed that HVRLocator correctly identified true mismatches with the reported metadata. In several such cases, we found that the discrepancies arose from metadata errors rather than model misclassification. These actions are stated in lines 263-283 of the manuscript:

Lines 263-283: "To check the reliability of HVRLocator relative to manual extraction of metadata from the literature, we manually extracted data related to the primers used and the 16S rRNA HV region targets from all samples in dataset c. For a total of 18,426 samples, 16,771 samples were processed successfully without warnings; common issues included missing FASTQ files, low reads, alignment failures, and NCBI portal-related issues. Of the 16,771 samples processed, 1,712 (10%) did not produce results consistent with the literature (e.g. mismatches between the start region alignment and the reported primer, or incorrect HV region alignment compared with the reported HV region), underscoring the value of obtaining metadata from the sequence data directly, rather than from the literature. Finally, for the diverse data set that used both different 16S rRNA regions and sequencing setups (Figure 2d), HVRLocator accurately and rapidly assigned the alignment positions.
Importantly, validation highlighted HVRLocator tool's ability to identify problematic sequences. For example, we observed 932 samples with abnormally long average sequence lengths (i.e., >600 bp) that exceeded the expected output lengths with Illumina platforms. Upon manually reviewing these sequences, we found that either the sequencing platform was incorrectly annotated in the metadata (NCBI or the associated publication), or the sequences did not correspond to the 16S rRNA gene but rather to the Internal Transcribed Spacer (ITS) region or the nifH gene. This highlights the use of HVRLocator as a curation tool for large datasets, where human errors in annotation can significantly impact downstream analysis."

4. Dataset imbalance in the Random Forest model
For the Random Forest model, the authors used 882 samples with primers and 8,940 samples without primers for training. Such an extremely imbalanced dataset, even with stratified sampling, may cause the model to be biased towards the majority class.

R/ We thank the reviewer for this important point. We evaluated performance using class-specific metrics achieving a precision of 100% and recall of 99.55% for the "primer-present" class on the held-out test set. These figures demonstrate that the model retained strong sensitivity and specificity for the minority class

despite the imbalance. Random Forests are known to perform robustly under moderate imbalance when class-aware evaluation and sampling are applied (Chen et al., 2004; Saito & Rehmsmeier, 2015). We have added text to manuscript in lines 139-145 that highlights this aspect of RF models. We also acknowledge that future versions could incorporate additional balancing techniques (e.g., class weighting), but the current results give confidence that no skew bias is affecting performance.

To confirm the robustness of the RF model, we applied the same stratified sampling during the 80/20 train-test split and ensured that both classes were adequately represented in each fold (lines 152-158 of the manuscript). This "balanced" model (which was trained using 881 no-primer samples and 881 primer samples) yielded virtually identical performance compared to the original: primer recall remained at 0.994, primer precision at 1.000, and overall accuracy exceeded 99.7%. This confirms that the initial model was not biased by the class imbalance and that the features used provide strong separation between classes:

Lines 152-158: "The model was trained using scikit-learn's RandomForestClassifier (v1.2.1) with 100 estimators and a fixed random seed (random_state=42), using an 80/20 stratified train-test split. The Random Forest model yielded a precision of 99.96% for the dataset without primers and 100% for the dataset with primers. Recall of the model using the "no-primer" and "primer" dataset was 100% and 99.55%, respectively. Full details on the model generation including the algorithm, versions and packages are available in the Supplementary Table S3."

Lines 139-145: "RF performs well under moderate class imbalances when class-aware evaluation and sampling is applied [25,26]. Additionally, distinct quality score patterns are known to occur in the first few cycles of Illumina sequencing when base diversity is low [27] as is in the case of untrimmed primers at the start of reads. Quality score patterns have also been used to detect sequencing bias and artifacts by tools such as DADA2 [28] and Mapinsights [29]. Thus, this metric can serve as a proxy for detecting primer presence."

Chen, C., Liaw, A., & Breiman, L. (2004). Using random forest to learn imbalanced data. University of California, Berkeley, 110(1-12), 24.

Saito, T., & Rehmsmeier, M. (2015). The precision-recall plot is more informative than the ROC plot when evaluating binary classifiers on imbalanced datasets. PloS one, 10(3), e0118432.

5. Comparison with existing tools
The manuscript mentions that no tool has been designed for this specific purpose, but this may overlook some existing general-purpose tools or scripts. Many pipelines (such as certain plugins in QIIME 2, USEARCH, etc.) possess functionalities to identify primers or evaluate amplicon regions. The authors should discuss how their tool compares to these existing workflows.

R/ Thank you for the comments. We have added the following text in lines 364-379:

Lines 364-379: "Additionally, some QIIME 2 plugins provide functionality that overlaps with certain steps of the HVRLocator workflow. For example, q2-cutadapt [38] uses cutadapt to remove adapter sequences, primers, and other unwanted sequences from high-throughput sequencing reads, thereby ensuring clean data for downstream analysis, but it requires prior knowledge of the exact primer or unwanted sequences used in the samples in order to accurately detect and remove them. Similarly, the quality-control filter-reads plugin [38] filters demultiplexed single- or paired-end sequences based on their alignment to a reference database using Bowtie 2 [39] and SAMtools [40] to remove contaminants (e.g., human DNA) or to retain only sequences that align to a specified reference, but also relies on alignment to external reference sequences and does not report hypervariable regions or primer information. USEARCH provides functions related to primer matching (e.g., search_oligodb, search_pcr, and search_pcr2) [41]. However, as with QIIME 2, users must supply the primer sequences in advance to perform database matching. In contrast, HVRLocator eliminates the need for prior knowledge of the exact primer sequences by automatically inferring this information."

Minor Comments
1. Confusion regarding processing speed metrics
The abstract mentions a processing speed of "0.147 samples per minute", but later the text mentions "6.5 samples per minute" and "one sample every 0.147 minutes". There is confusion regarding units and values in these three descriptions (is it samples per minute or minutes per sample?). Please unify and correct these data to ensure consistency.

R/ Thank you. The reviewer is correct. There is no confusion regarding the units or values; however, we agree that the message was not clear or fully consistent. The information was derived from Table S5.

The value reported in the phrase "...at an average rate of 6.5 samples per minute" corresponds to the mean of the values in the last column ("Samples processed per minute").

The value reported in the statement "one sample every 0.147 minutes" corresponds to the mean of the column labeled "Time to process 1 sample."

For greater clarity, we have revised the text as follows:

1.Abstract: "HVRLocator can process archived 16S rRNA sequences from NCBI SRA at 6.5 samples per minute."
We modified the sentence as follows: "HVRLocator can process archived 16S rRNA sequences from NCBI SRA at an average rate of 6.5 samples per minute."

3.Case Study: "HVRLocator processed approximately one sample every 0.147 minutes, using 8 GB of RAM and 4 CPU cores."
We modified the sentence as follows: "HVRLocator processed samples at an average rate of 6.5 samples per minute, using 8 GB of RAM and 4 CPU cores."

2. Usage of fastq-dump
The use of fastq-dump is mentioned. The SRA Toolkit's fastq-dump is relatively slow and has largely been superseded by fasterq-dump for efficiency. Why did the authors not use the more efficient fasterq-dump?

R/ We thank the reviewer for pointing this out. We agree that fasterq-dump is generally more efficient and represents the recommended approach for large-scale data retrieval. In the current implementation, however, we download only the first 10,000 reads from each sample for primer inference. Under these conditions, the overall runtime is dominated by the time required to establish connections to the server rather than by the data transfer itself, and the use of a multi-threaded tool such as fasterq-dump does not result in a substantial performance improvement. Nevertheless, we acknowledge the reviewer's suggestion and will consider adopting fasterq-dump in future versions of the tool to improve efficiency.

3. Definition of "Standardized metadata"
The term "standardized metadata" is used frequently. Please explicitly define what constitutes "standard" metadata in the context of this tool within the text.

R/ Thank you for the observation. We have added the following text in lines 95-98:

Lines 95-98: "Standardized metadata is structured information that follows agreed-upon standards, ensuring consistency and comparability across studies and databases through defined fields, controlled vocabularies or ontologies, and standardized formats."

4. Robustness and error handling
The results section mentions that some samples failed due to "NCBI portal-related issues". Does this imply the tool lacks breakpoint resumption or retry mechanisms?

R/ We apologize for the confusion. The tool includes a retry mechanism to handle temporary NCBI portal

issues. The reported failures were due to persistent external server problems rather than a lack of retry mechanisms in the tool. We have added this clarification to the results section:

Lines 239-241: “Although HVRLocator incorporates an automatic retry mechanism to mitigate temporary NCBI portal interruptions, a small number of samples failed due to persistent external server-side issues beyond the control of the tool.”

Given that network fluctuations are common during large-scale downloads, how is the tool's robustness demonstrated?

R/ For any error in the download of a dataset using “fastq-dump”, we have implemented a retry function that tries to retrieve the data three times before moving to the next entry. This way we assure to bypass any temporary issues at the servers level.
Lastly, at the end of a whole run, the user can select the IDs of the failed entries and submit again to a new run. We opted to not automatically try the “failed” downloads again at the end and let the user decide what to do with those. From our experience, these runs are usually simply not available in the Sequence Read Archive (SRA) (Jurburg et al, 2020).

Jurburg, S.D., Konzack, M., Eisenhauer, N. and Heintz-Buschart, A. (2020) The archives are half-empty: an assessment of the availability of microbial community sequencing data. Communications Biology; 3 (474)

5. Output confidence intervals
The output file contains "TRUE/FALSE" and a probability score. For samples where the probability score is at a critical threshold (e.g., around 0.5), does the tool provide an "uncertain" tag, or does it force a classification? It is suggested to add an indicator for ambiguous ranges.

R/ The cutoff value used (0.5) represents a validated and balanced threshold that is not overly conservative, thereby avoiding an excessive number of false positives. However, we provide users with the underlying probability scores values, which can be manually adjusted directly within the results table to apply custom filtering according to their preferences. This allows users to disregard the binary TRUE/FALSE classification and instead filter results based solely on the probability score.
HVRLocator uses the 0.5 threshold by default because it was validated using the Random Forest model. Any alternative threshold selected by the user is applied at their own judgment and responsibility.

We add the following text in the main manuscript in order to clarify this:

Line 196-200:“Additionally, HVRLocator provides the underlying probability scores for primer presence, allowing users to manually adjust filtering based on their own criteria instead of relying solely on the default TRUE/FALSE classification. The default 0.5 threshold was validated using the Random Forest model, and any alternative threshold chosen by the user is applied at their own discretion.”

--
6. Please also take a moment to check our website at https://www.editorialmanager.com/giga/l.asp?i=238196&l=G6VFB430 for any additional comments that were saved as attachments. Please note that as GigaScience has a policy of open peer review, you will be able to see the names of the reviewers.

R/ Done.

In compliance with data protection regulations, you may request that we remove your personal registration details at any time. (Use the following URL: https://www.editorialmanager.com/giga/login.asp?a=r). Please contact the publication office if you have any questions.

Editorial Manager®

Cookie Preference Center

We use cookies which are necessary to make our site work. We may also use additional cookies to analyze, improve and personalize our content and your digital experience. For more information, see our Cookie Policy.

You may choose not to allow some types of cookies. However, blocking some types may impact your experience of our site and the services we are able to offer. See the different category headings below to find out more or change your settings.

Allow all

Manage Consent Preferences

Strictly Necessary Cookies

Always active

These cookies are necessary for the website to function and cannot be switched off in our systems. They are usually only set in response to actions made by you which amount to a request for services, such as setting your privacy preferences, logging in or filling in forms. You can set your browser to block or alert you about these cookies, but some parts of the site will not then work.

Functional Cookies

These cookies enable the website to provide enhanced functionality and personalisation. They may be set by us or by third party providers whose services we have added to our pages. If you do not allow these cookies then some or all of these services may not function properly.

Performance Cookies

These cookies allow us to count visits and traffic sources so we can measure and improve the performance of our site. They help us to know which pages are the most and least popular and see how visitors move around the site.

Targeting Cookies

These cookies may be set through our site by our advertising partners. They may be used by those companies to build a profile of your interests and show you relevant adverts on other sites. If you do not allow these cookies, you will experience less targeted advertising.

Cookie List

Clear

Apply Cancel
Consent Leg.Interest

Confirm my choices

Power
