# Supplementary material for: HVRLocator: a computationally efficient tool for identifying hypervariable regions in large 16S rRNA datasets
Source: Gigascience. 2026 Apr 8;15:giag040. doi: 10.1093/gigascience/giag040 (PMC13188219; doi:10.1093/gigascience/giag040)
Supplement: giag040_Authors_Response_To_Reviewer_Comments_revision_1 [file giag040_authors_response_to_reviewer_comments_revision_1.pdf]

Author's Response To Reviewer Comments

GIGA-D-25-00344R1
HVRLocator: A Computationally Efficient Tool for Identifying Hypervariable Regions in Large 16S rRNA Datasets
Clara Arboleda-Baena; Felipe Borim Correa; Joao Pedro Saraiva; Santiago Castillo-Rivadeneira; Jonas Coelho Kasmanas; Antonis Chatzinotas; Stephanie D. Jurburg
GigaScience

Dear Dr Arboleda-Baena,

Your manuscript "HVRLocator: A Computationally Efficient Tool for Identifying Hypervariable Regions in Large 16S rRNA Datasets" (GIGA-D-25-00344R1) has been assessed by our reviewers. Based on these reports, and my own assessment as Editor, I am pleased to inform you that it is potentially acceptable for publication in GigaScience, once you have carried out some essential revisions suggested by our reviewers.

Their reports, together with any other comments, are below. Please also take a moment to check our website at https://www.editorialmanager.com/giga/ for any additional comments that were saved as attachments.

In addition, please register any new software application in the bio.tools and SciCrunch.org databases to receive RRID (Research Resource Identification Initiative ID) and biotoolsID identifiers, and include these in your manuscript. Computational workflows should be registered in workflowhub.eu and the DOIs cited in the relevant places in the manuscript. These will facilitate tracking, reproducibility and re-use of your tool.

Once you have made the necessary corrections, please submit a revised manuscript online at:

https://www.editorialmanager.com/giga/

If you have forgotten your username or password please use the "Send Login Details" link to get your login information. For security reasons, your password will be reset.

Please include a point-by-point within the 'Response to Reviewers' box in the submission system. Please ensure you describe additional experiments that were carried out and include a detailed rebuttal of any criticisms or requested revisions that you disagreed with. Please also ensure that your revised manuscript conforms to the journal style, which can be found in the Instructions for Authors on the journal homepage. If the data and code has been modified in the revision process please be sure to update the public versions of this too.

The due date for submitting the revised version of your article is 27 Mar 2026.

We look forward to receiving your revised manuscript soon.

Best wishes,

Dongni Ma, Ph.D.
GigaScience
www.gigasciencejournal.com

R/ Thank you very much, attached you can find the Response to Reviewers.

Reviewer reports:
Reviewer #1: I believe that the authors have addressed the concerns that I raised - in particular the question about whether using a single E. coli sequence would detect divergent bacteria which was raised by the other referee too. If I have understood their methodology correctly they are characterising a sample at

a time and while there may be some individual sequences that do not "match" [sensu lato] E. coli there will almost always be sequences that are sufficiently similar to E. coli for their method to work. In their response to referees, the authors say:

"Even for bacterial taxa that are highly divergent from E. coli, the 16S rRNA gene remains highly conserved across Bacteria, ensuring sufficient sequence similarity for reliable alignment to the appropriate reference region. Although 16S rRNA sequences vary in length among taxa, our validation showed 99% agreement between the predicted hypervariable regions and those reported in the original publications (metadata or NCBI records). Together, the conserved structure of the 16S rRNA gene and the strong empirical validation support the conclusion that the current alignment strategy provides reliable estimates of start and end positions for hypervariable regions in Bacteria and adequately addresses the objectives of this study."

I would reccommend that the sense of this response is included more explicitly into the final manuscript, perhaps with a cross reference to supplementary material, and that the authors check through their other responses to the referees to make sure that the arguments that they provided there are included within the main part of the manuscript.

R/ We reviewed the responses in the previous file “Response to Reviewers_GIGA-D-25-00344” and found that most of the reviewers’ concerns corresponded to comments that had already been addressed in the main manuscript. However, we have taken the reviewer’s final suggestion into account and added the following text to the main manuscript:

Lines 275-283: “Importantly, validation highlighted HVRLocator tool’s ability to identify problematic sequences. For example, we observed 932 samples with abnormally long average sequence lengths (i.e., >600 bp) that exceeded the expected output lengths with Illumina platforms. Upon manually reviewing these sequences, we found that either the sequencing platform was incorrectly annotated in the metadata (NCBI or the associated publication), or the sequences did not correspond to the 16S rRNA gene but rather to the Internal Transcribed Spacer (ITS) region or the nifH gene. This highlights the use of HVRLocator as a curation tool for large datasets, where human errors in annotation can significantly impact downstream analysis. For further details, see Supplementary Table S6.”

We also added a new supplementary table (Supplementary Table S6), which contains the following:

Even for bacterial taxa that are highly divergent from E. coli, the 16S rRNA gene remains highly conserved across Bacteria, ensuring sufficient sequence similarity for reliable alignment to the appropriate reference region. Although 16S rRNA sequences vary in length among taxa, our validation showed 99% agreement between the predicted hypervariable regions and those reported in the original publications (metadata or NCBI records). Together, the conserved structure of the 16S rRNA gene and the strong empirical validation support the conclusion that the current alignment strategy provides reliable estimates of start and end positions for hypervariable regions in Bacteria and adequately addresses the objectives of this study.

Regarding Archaea and Eukaryotes, we performed several tests and identified potential warning signs that may alert users to review the metadata of sequences exhibiting the following patterns:

1. Putative ITS region: Sequences showing coverage across more than four or five hypervariable (HV) regions. In such cases, users should verify the following information in the metadata: the sequencing platform. If the platform used for these sequences is not PacBio or Nanopore, the primers may be targeting regions that are not specific to bacteria. If the mentioned platforms were used, users should also verify that the primers are not targeting Archaea or Eukaryotes.

The following table presents examples of ITS regions analyzed using HVRLocator:
Sample_ID Primer Presence Score Primer Presence Min Alignment start Max Alignment end Average Alignment start Average Alignment end Median Alignment start Median Alignment end Predicted HV region start Predicted HV region end Coverage based HV region start Coverage based HV region end Coverage HV region start Coverage HV region end Warnings
SRR24285816 FALSE 0.17 0 1540 374 1068 399 1148 V3 V7 V4 V7 0.45 0.72 V3 below threshold of 0.5
SRR24285817 FALSE 0.17 0 1540 398 992 399 1110 V3 V7 V4 V6 0.45 0.39 V3 and V7 below threshold of

0.5
SRR24285818 FALSE 0.17 0 1540 400 1018 399 1110 V3 V7 V4 V6 0.45 0.39 V3 and V7 below threshold of 0.5
SRR24285819 FALSE 0.17 0 1540 402 994 399 1110 V3 V7 V4 V6 0.45 0.39 V3 and V7 below threshold of 0.5
SRR24285820 FALSE 0.17 0 1540 446 1017 399 1110 V3 V7 V4 V6 0.45 0.39 V3 and V7 below threshold of 0.5
SRR24285821 FALSE 0.16 0 1540 431 1000 399 1110 V3 V7 V4 V6 0.45 0.39 V3 and V7 below threshold of 0.5
SRR24285822 FALSE 0.17 0 1540 374 953 399 1110 V3 V7 V4 V6 0.45 0.39 V3 and V7 below threshold of 0.5
SRR24285823 FALSE 0.17 0 1540 392 961 399 1010 V3 V6 V4 V6 0.45 0.69 V3 below threshold of 0.5
SRR24285824 FALSE 0.17 0 1540 392 968 399 953 V3 V6 V4 V5 0.45 0.38 V3 and V6 below threshold of 0.5
SRR24285825 FALSE 0.17 0 1540 401 963 399 953 V3 V6 V4 V5 0.45 0.38 V3 and V6 below threshold of 0.5
SRR25665816 FALSE 0.17 0 1540 357 872 186 826 V2 V5 V2 V4 0.56 0.2 V5 below threshold of 0.5
SRR25665819 FALSE 0.2 0 1540 690 1172 903 1450 V6 V9 V6 V8 0.91 0.34 V9 below threshold of 0.5
SRR25665821 FALSE 0.22 0 1540 425 956 186 826 V2 V5 V2 V4 0.56 0.2 V5 below threshold of 0.5
SRR25665824 FALSE 0.2 0 1540 627 1210 685 1210 V4 V8 V5 V7 0.38 0.16 V4 and V8 below threshold of 0.5
SRR25665826 FALSE 0.22 0 1540 602 1118 574 1048 V4 V6 V4 V6 0.72 0.91 NA
SRR25665828 FALSE 0.2 0 1540 303 1164 177 1096 V2 V7 V2 V6 0.61 0.27 V7 below threshold of 0.5
SRR25665830 FALSE 0.17 0 1540 600 1149 529 1048 V4 V6 V4 V6 0.85 0.91 NA
SRR25665832 FALSE 0.21 0 1540 449 970 186 826 V2 V5 V2 V4 0.56 0.2 V5 below threshold of 0.5
ERR5924913 FALSE 0.17 0 1540 482 1055 548 1111 V4 V7 V4 V6 0.8 0.4 V7 below threshold of 0.5
ERR5924916 FALSE 0.17 0 1540 463 1076 595 1177 V4 V7 V4 V7 0.65 0.97 NA

2. Putative Archaea or 18S hypervariable region: As clarified in the manuscript, users must ensure that the target domain is Bacteria. Due to similarities in gene length and conserved regions, sequences targeting archaeal 16S rRNA genes or eukaryotic 18S rRNA genes may still produce a prediction when analyzed with HVRLocator. However, the tool is specifically designed and validated for bacterial 16S rRNA sequences. Therefore, verification of the sample metadata is essential to confirm that the predicted hypervariable region corresponds to bacterial sequences.

Reviewer #2: The authors have made proactive improvements in both the functional flexibility of the program (supporting custom reference sequences) and the expansion of the taxonomic scope (mentioning future support for ITS, etc.); therefore, this manuscript would be of significant value for publication as a Technical Note in GigaScience.

R/ We thank the reviewer for their positive feedback.

--
Please also take a moment to check our website at https://www.editorialmanager.com/giga/l.asp?i=242341&l=0KJITSI3 for any additional comments that were saved as attachments. Please note that as GigaScience has a policy of open peer review, you will be able to see the names of the reviewers.

R/ Done, thank you.

Cookie Preference Center

We use cookies which are necessary to make our site work. We may also use additional cookies to analyze, improve and personalize our content and your digital experience. For more information, see our Cookie Policy.

You may choose not to allow some types of cookies. However, blocking some types may impact your experience of our site and the services we are able to offer. See the different category headings below to find out more or change your settings.

Allow all

Manage Consent Preferences

Strictly Necessary Cookies

Always active

These cookies are necessary for the website to function and cannot be switched off in our systems. They are usually only set in response to actions made by you which amount to a request for services, such as setting your privacy preferences, logging in or filling in forms. You can set your browser to block or alert you about these cookies, but some parts of the site will not then work.

Functional Cookies

These cookies enable the website to provide enhanced functionality and personalisation. They may be set by us or by third party providers whose services we have added to our pages. If you do not allow these cookies then some or all of these services may not function properly.

Performance Cookies

These cookies allow us to count visits and traffic sources so we can measure and improve the performance of our site. They help us to know which pages are the most and least popular and see how visitors move around the site.

Targeting Cookies

These cookies may be set through our site by our advertising partners. They may be used by those companies to build a profile of your interests and show you relevant adverts on other sites. If you do not allow these cookies, you will experience less targeted advertising.

Cookie List

Clear

Apply Cancel

Consent Leg.Interest

Confirm my choices

Power
