# Supplementary material for: HVRLocator: a computationally efficient tool for identifying hypervariable regions in large 16S rRNA datasets
Source: Gigascience. 2026 Apr 8;15:giag040. doi: 10.1093/gigascience/giag040 (PMC13188219; doi:10.1093/gigascience/giag040)
Supplement: giag040_Reviewer_1_Report_original_submission — Reviewer 1 -- 10/13/2025 [file giag040_reviewer_1_report_original_submission.pdf]

Reviewer Report

Title: HVRLocator: A Computationally Efficient Tool for Identifying Hypervariable Regions in Large 16S rRNA Datasets

Version: Original Submission **Date:** 10/13/2025

Reviewer name: Reviewer 1

Reviewer Comments to Author:

Metabarcoding data are accumulating rapidly. This paper makes a very valuable contribution to the automated extraction and curation of metabarcoding data and should be of great value in facilitating the re-use of existing data and the construction of custom databases based on these.
I have not tested or tried to install the software myself, as the manuscript provided sufficient detail to enable me to assess the tool
General comments
The manuscript is written entirely in terms of "bacteria" and aligns amplicons to an E. coli model sequence. This is reasonable, but there should certainly be some acknowledgement of Archaea and ideally some mention of Eukaryotes too. These are probably things for the discussion section of this manuscript, but the authors may wish to consider whether a future version of the program could contain options to use model Archaea and Eukaryote sequences as alternatives to the E. coli model.
It would also be helpful to assess how the program with its E. coli model deals with sequence data from Archaea, Eukaryotes (including mitochondria) and bacteria that are very divergent from E. coli.
The methods section does not contain details of software used to generate the figures, or whether these figures are produced by "the pipeline" or by separate analysis of the .txt file that the pipeline produces. I suspect that it is that latter, in which case making the authors should make the scripts used available - as well as providing complete documentation of what has been done, this is likely to increase use made of the tool.
And it would be helpful to include an output file in the supplementary materials
Specific comments
Line 64 "however the integration of these data in light of processing metadata" - not clear
Line 67-8 "though bacterial diversity increases linearly with amplicon length".
Needs re-wording. The number of ASVs will increase with amplicon length, but the actual bacterial diversity in a sample is constant.
Line 79 "Wasimuddin and colleagues" should be "Wasimuddin et al". More generally, check that citations conform with journal house style
Line 79-82 "For example, Wasimuddin and colleagues [8] found that compared to three other primer sets targeting different regions, the primer pair targeting the V4 hypervariable region of the 16S rRNA gene produced the highest estimates of species richness and diversity across various sample types"
There are three issues here:
1) different primer pairs vary in their coverage and bias, so different primers targeting the same variable region will produce different numbers of ASVs

2) Even with complete coverage and the absence of bias, different variable regions will generate different numbers of ASVs as a result of differences in length and rate of evolution between variable regions (and differences in the number of ASVs that are clustered into OTUs at a particular sequence similarity threshold

3) The relationship between ASVs or OTUs and "species" is not straightforward (Edgar, 2018).

At minimum "species" should be replace with ASV or OTU (whichever Wasimuddin et al used)

Line 89-90 "as bacterial diversity and taxonomic resolution linearly increase with target sequence length [12]." Overlaps with statement made in line 67-8, and the same issue applies here.

Lines 167-170. The output file contains (amongst other things) "Predicted HV region Start/End: Predicted hypervariable (HV) region based on the median alignment start and end positions across all reads, inferred from literature on conserved and hypervariable regions of the 16S rRNA gene (Brosius et al., 1978; Yang et al., 2016)". This implies that the program predicts a single variable region for each study - I am not clear what this column will contain for amplicons that contain more than one variable region, although columns 11-19 indicate that the program identifies the presence/absence of each of the 9 HV regions. My guess is that the authors are using "HV region" in two different sense:

1) Its usual meaning of one region out of V1 to V9

2) The sequence from the beginning of the first of the nine variable regions the amplicon includes to the end of the last.

It would also be helpful to indicate whether the sequence positions here are relative to the E coli model or refer to sequence positions in the amplicon

Edgar, R. C. (2018). Updating the 97% identity threshold for 16S ribosomal RNA OTUs. Bioinformatics, 34(14), 2371-2375. doi:10.1093/bioinformatics/bty113

Methods

Are the methods appropriate to the aims of the study, are they well described, and are necessary controls included? Choose an item.

Conclusions

Are the conclusions adequately supported by the data shown? Choose an item.

Reporting Standards

Does the manuscript adhere to the journal's guidelines on [minimum standards of reporting?]() Choose an item.

Choose an item.

Statistics

Are you able to assess all statistics in the manuscript, including the appropriateness of statistical tests used? Choose an item.

Quality of Written English

Please indicate the quality of language in the manuscript: Choose an item.

Declaration of Competing Interests

Please complete a declaration of competing interests, considering the following questions:

- Have you in the past five years received reimbursements, fees, funding, or salary from an organisation that may in any way gain or lose financially from the publication of this manuscript, either now or in the future?
- Do you hold any stocks or shares in an organisation that may in any way gain or lose financially from the publication of this manuscript, either now or in the future?
- Do you hold or are you currently applying for any patents relating to the content of the manuscript?
- Have you received reimbursements, fees, funding, or salary from an organization that holds or has applied for patents relating to the content of the manuscript?
- Do you have any other financial competing interests?
- Do you have any non-financial competing interests in relation to this paper?

If you can answer no to all of the above, write 'I declare that I have no competing interests' below. If your reply is yes to any, please give details below.

I declare that I have no competing interests

I agree to the open peer review policy of the journal. I understand that my name will be included on my report to the authors and, if the manuscript is accepted for publication, my named report including any attachments I upload will be posted on the website along with the authors' responses. I agree for my report to be made available under an Open Access Creative Commons CC-BY license (http://creativecommons.org/licenses/by/4.0/). I understand that any comments which I do not wish to be included in my named report can be included as confidential comments to the editors, which will not be published.

Choose an item.

To further support our reviewers, we have joined with Publons, where you can gain additional credit to further highlight your hard work (see: https://publons.com/journal/530/gigascience). On publication of this paper, your review will be automatically added to Publons, you can then choose whether or not to claim your Publons credit. I understand this statement.

Yes Choose an item.
