# Supplementary material for: HVRLocator: a computationally efficient tool for identifying hypervariable regions in large 16S rRNA datasets
Source: Gigascience. 2026 Apr 8;15:giag040. doi: 10.1093/gigascience/giag040 (PMC13188219; doi:10.1093/gigascience/giag040)
Supplement: giag040_Reviewer_2_Report_original_submission — Reviewer 2 -- 12/6/2025 [file giag040_reviewer_2_report_original_submission.pdf]

**Reviewer Report**

**Title: HVRLocator: A Computationally Efficient Tool for Identifying Hypervariable Regions in Large 16S rRNA Datasets**

**Version: Original Submission** **Date:** 12/6/2025

**Reviewer name: Reviewer 2**

**Reviewer Comments to Author:**

General Comments

This manuscript introduces a tool named HVRLocator, designed to address the issue of missing or non-standard metadata in 16S rRNA sequencing data found in public databases such as the SRA. The tool identifies amplicon regions by aligning sequences to a reference genome and attempts to detect the presence of primers using a machine learning model. This is a subject with significant practical value, particularly for conducting large-scale meta-analyses. However, there are still many issues regarding methodological rigor, the depth of validation, and comparisons with existing tools that require further clarification by the authors.

Major Comments

1. Concerns regarding the singularity of the reference sequence

The authors mention aligning sequences to a single Escherichia coli (J01859.1) reference genome to determine start and end positions. Is a single E. coli reference sufficient to cover Archaea or bacterial phyla that are distantly related to Proteobacteria, which may be present in environmental samples (e.g., soil, ocean)? For taxa with significant length variations or insertions/deletions (Indels), could forced alignment to the E. coli reference lead to misjudgment of start/end positions? Have the authors evaluated the impact on accuracy if a more universal reference database (such as representative sequences from SILVA or Greengenes) were used?

2. Rationality of the primer detection model (Random Forest based on Quality Scores)

The authors developed a Random Forest model to predict primer presence by analyzing the quality score distribution of the first 1,000 reads. Primer detection is typically based on the sequence itself rather than quality scores. Can the authors explain why quality scores were chosen as features? Sequencing quality scores are influenced by technical factors such as sequencer status, reagent batches, and run cycles, which have no direct biological correlation with the presence of primers. Is there a risk that this model is "overfitting" specific sequencing platforms or datasets? Since the reads are already downloaded, why not directly use degenerate primer sequence matching (e.g., using Cutadapt or SeqKit logic) to determine primer presence? This seems to be a more direct and accurate method.

3. Verification of accuracy claims

In the validation section, the authors claim to achieve 100% accuracy on certain datasets. In bioinformatics tool development, a claim of 100% accuracy is often a red flag. Have the authors manually checked those samples marked as "correct" by the model that might suffer from edge effects or borderline cases?

4. Dataset imbalance in the Random Forest model

For the Random Forest model, the authors used 882 samples with primers and 8,940 samples without primers for training. Such an extremely imbalanced dataset, even with stratified sampling, may cause the model to be biased towards the majority class.

5. Comparison with existing tools

The manuscript mentions that no tool has been designed for this specific purpose, but this may overlook some existing general-purpose tools or scripts. Many pipelines (such as certain plugins in QIIME 2, USEARCH, etc.) possess functionalities to identify primers or evaluate amplicon regions. The authors should discuss how their tool compares to these existing workflows.

Minor Comments

1. Confusion regarding processing speed metrics

The abstract mentions a processing speed of "0.147 samples per minute", but later the text mentions "6.5 samples per minute" and "one sample every 0.147 minutes". There is confusion regarding units and values in these three descriptions (is it samples per minute or minutes per sample?). Please unify and correct these data to ensure consistency.

2. Usage of fastq-dump

The use of fastq-dump is mentioned. The SRA Toolkit's fastq-dump is relatively slow and has largely been superseded by fasterq-dump for efficiency. Why did the authors not use the more efficient fasterq-dump?

3. Definition of "Standardized metadata"

The term "standardized metadata" is used frequently. Please explicitly define what constitutes "standard" metadata in the context of this tool within the text.

4. Robustness and error handling

The results section mentions that some samples failed due to "NCBI portal-related issues". Does this imply the tool lacks breakpoint resumption or retry mechanisms? Given that network fluctuations are common during large-scale downloads, how is the tool's robustness demonstrated?

5. Output confidence intervals

The output file contains "TRUE/FALSE" and a probability score. For samples where the probability score is at a critical threshold (e.g., around 0.5), does the tool provide an "uncertain" tag, or does it force a classification? It is suggested to add an indicator for ambiguous ranges.

**Methods**

Are the methods appropriate to the aims of the study, are they well described, and are necessary controls included? Choose an item.

**Conclusions**

Are the conclusions adequately supported by the data shown? Choose an item.

**Reporting Standards**

Does the manuscript adhere to the journal's guidelines on [minimum standards of reporting?]() Choose an item.

Choose an item.

**Statistics**

Are you able to assess all statistics in the manuscript, including the appropriateness of statistical tests used? Choose an item.

**Quality of Written English**

Please indicate the quality of language in the manuscript: Choose an item.

**Declaration of Competing Interests**

Please complete a declaration of competing interests, considering the following questions:

- Have you in the past five years received reimbursements, fees, funding, or salary from an organisation that may in any way gain or lose financially from the publication of this manuscript, either now or in the future?
- Do you hold any stocks or shares in an organisation that may in any way gain or lose financially from the publication of this manuscript, either now or in the future?
- Do you hold or are you currently applying for any patents relating to the content of the manuscript?
- Have you received reimbursements, fees, funding, or salary from an organization that holds or has applied for patents relating to the content of the manuscript?
- Do you have any other financial competing interests?
- Do you have any non-financial competing interests in relation to this paper?

If you can answer no to all of the above, write 'I declare that I have no competing interests' below. If your reply is yes to any, please give details below.

I declare that I have no competing interests

I agree to the open peer review policy of the journal. I understand that my name will be included on my report to the authors and, if the manuscript is accepted for publication, my named report including any attachments I upload will be posted on the website along with the authors' responses. I agree for my report to be made available under an Open Access Creative Commons CC-BY license (http://creativecommons.org/licenses/by/4.0/). I understand that any comments which I do not wish to be included in my named report can be included as confidential comments to the editors, which will not be published.

Choose an item.

To further support our reviewers, we have joined with Publons, where you can gain additional credit to further highlight your hard work (see: https://publons.com/journal/530/gigascience). On publication of this paper, your review will be automatically added to Publons, you can then choose whether or not to claim your Publons credit. I understand this statement.

Yes Choose an item.
